# Isolation and In Silico Inhibitory Potential against SARS-CoV-2 RNA Polymerase of the Rare Kaempferol 3-*O*-(6″-*O*-acetyl)-Glucoside from *Calligonum tetrapterum*

**DOI:** 10.3390/plants11152072

**Published:** 2022-08-08

**Authors:** Yerlan M. Suleimen, Rani A. Jose, Gulnur K. Mamytbekova, Raigul N. Suleimen, Margarita Y. Ishmuratova, Wim Dehaen, Bshra A. Alsfouk, Eslam B. Elkaeed, Ibrahim H. Eissa, Ahmed M. Metwaly

**Affiliations:** 1 Technopark Department, Kazakh University of Technology and Business, Nur-Sultan 010000, Kazakhstan; 2 The Laboratory of Engineering Profile of NMR Spectroscopy, Sh. Ualikhanov Kokshetau University, Kokshetau 020000, Kazakhstan; 3 Molecular Design & Synthesis KU Leuven, Department of Chemistry, Celestijnenlaan 200F, B-3001 Leuven, Belgium; 4 Department of Chemistry, St. Dominic’s College, Mahatma Gandhi University, Kanjirappally 686512, India; 5 Department of Technical Physics, Faculty of Physics and Technology, L.N. Gumilyov Eurasian National University, Nur-Sultan 010010, Kazakhstan; 6 Department of Botany, E.A. Buketov Karaganda University, Karaganda 100024, Kazakhstan; 7 Department of Pharmaceutical Sciences, College of Pharmacy, Princess Nourah bint Abdulrahman University, P.O. Box 84428, Riyadh 11671, Saudi Arabia; 8 Department of Pharmaceutical Sciences, College of Pharmacy, AlMaarefa University, Riyadh 13713, Saudi Arabia; 9 Pharmaceutical Medicinal Chemistry & Drug Design Department, Faculty of Pharmacy (Boys), Al-Azhar University, Cairo 11884, Egypt; 10 Pharmacognosy and Medicinal Plants Department, Faculty of Pharmacy (Boys), Al-Azhar University, Cairo 11884, Egypt; 11 Biopharmaceutical Product Research Department, Genetic Engineering and Biotechnology Research Institute, City of Scientific Research and Technological Applications (SRTA-City), Alexandria 21934, Egypt

**Keywords:** *Calligonum tetrapterum*, SARS-CoV-2 RNA-dependent RNA polymerase, structural similarity, DFT, molecular docking, ADMET, MD simulations

## Abstract

The phytochemical constituents of *Calligonum tetrapterum* Jaub. & Spach (Family Polygonaceae) were studied for the first time. The study resulted in the isolation of the rare flavonol glycoside, kaempferol 3-*O*-(6″-*O*-acetyl)-glucoside,(K3G-A). The potential inhibitive activity of K3G-A toward SARS-CoV-2 was investigated utilizing several in silico approaches. First, molecular fingerprints and structural similarity experiments were carried out for K3G-A against nine co-crystallized ligands of nine proteins of SARS-CoV-2 to reveal if there is a structural similarity with any of them. The conducted studies showed the high similarity of K3G-A and remdesivir, the co-crystallized ligand of SARS-CoV-2 RNA-dependent RNA polymerase (PDB ID: 7BV2), RdRp. To validate these findings, a DFT study was conducted and confirmed the proposed similarity on the electronic and orbital levels. The binding of K3G-A against RdRp was confirmed through molecular docking studies exhibiting a binding energy of −27.43 kcal/mol, which was higher than that of remdesivir. Moreover, the RdRp-K3G-A complex was subjected to several MD studies at 100 ns that authenticated the accurate mode of binding and the correct dynamic behavior. Finally, in silico ADMET and toxicity evaluation of K3G-A was conducted and denoted the safety and the drug-likeness of K3G-A. In addition to K3G-A, two other metabolites were isolated and identified to be kaempferol (K) and β-sitosterol (β-S).

## 1. Introduction

Since the first historical records, natural products have provided humans with their principal needs in terms of food, treatment, or even cosmetics [1,2]. The phytochemical and biological properties of various plants in Kazakhstan, such as *Pulicaria vulgaris* [3,4], *Ferula* spp. [5,6], and *Cousinia alata* [7,8], have been previously studied. In addition, the essential oils of various plants have been investigated [9,10]. In continuation of this work, this study outlines the composition of *Calligonum tetrapterum* Jaub. & Spach. (Family Polygonaceae). *Calligonum tetrapterum* grows from Middle Asia to the Arabian Peninsula. In Kazakhstan, this species is found in Turgai, Aral region, Northern and Southern Balkhash, and Kyzyl-Kum desert [11].

*Calligonum tetrapterum* Jaub. & Spach (Family Polygonaceae) is a shrub up to 1–2 m tall, with dark-grey bark, without leaves. There are five perianth lobes, which are positioned downwards on the fruits. The fruit is a dry nut, with film edges; ovoid, 14–17 mm long, and 12–15 mm wide [12]. It is worth mentioning that this is the first phytochemical study of *Calligonum tetrapterum*.

The computational (in silico) chemistry approach is an effective tool in virtual biological screening and has been widely employed in the processes of drug design and drug discovery. This technique has been utilized to assess the biological activities of natural products, synthesized compounds, and semi-synthesized molecules. Advanced software has enabled scientists to utilize the principles of the structure–activity relationship as a tool to accurately predict the bioactivity of new and rare molecules depending on their chemical and physical properties. Several recent applications of computational chemistry have contributed to a better understanding of the nature of SARS-CoV-2 [13,14,15,16] and suggested various compounds as potential inhibitors [17,18,19].

Computer-based chemistry strategies have been employed to disclose the potential inhibitive effects of several secondary metabolites against SARS-CoV-2. The examined metabolites were isolated from *Asteriscus hierochunticus* [20], *Monanchora* sp. [21], *Artemisia sublessingiana* [22], *Artemisia commutata* [23], *Artemisia glauca* [24], *Chondrilla brevirostris* [25], and *Artemisia* spp. [26], in addition to 69 isoflavonoids [27]. The consumption of food and dietary supplements that are rich in phenolic content was found to be effective in the prevention of SARS-CoV-2 infection [28]. Moreover, flavonoids have exhibited promising activities against SARS-CoV-2 M^pro^ [29], viral replication [30], and infection severity [31], in addition to various other targets in SARS-CoV-2 [32].

Plants that belong to the genus *Calligonum* have exhibited cytotoxic [33], anti-inflammatory [34], antifungal [35], and antioxidant [36] activities.

This study isolated the rare flavonoid acetylated glucoside, K3G-A, from the aerial parts of *Calligonum tetrapterum*. Because K3G-A is a rare metabolite, its potential effect as a treatment for COVID-19 was examined. In addition, ADMET and toxicity descriptors of K3G-A were investigated to examine the drug likeness. Finally, several MD simulation studies were conducted and confirmed the predicted binding of K3G-A against RdRp. In addition to K3G-A, for the first time, two compounds were identified from *Calligonum tetrapterum* to be kaempferol (K) and β-sitosterol (β-S).

## 2. Results and Discussion

### 2.1. Isolation and Characterization 

**Figure 1:**
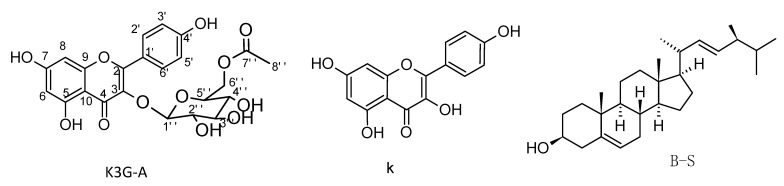
The isolated compounds.

The aerial parts of *Calligonum tetrapterum* were collected from Sarkand, Almaty region, Kazakhstan during the fruiting phase. The specimen was identified by Ishmuratova M.Yu. An herbarium sample was located in the herbarium fund of Zhezkazgan botanical garden (N2007.09.12.03.01). Fine raw material of *C. tetrapterum* (1.1 kg) was extracted three times with 70% ethanol by keeping it for 3 days at room temperature. The filtrates were evaporated on a rotary evaporator, and the resulting extract was subjected to several chromatographic isolation techniques. Firstly, chromatographic separation on silica gel was carried out using heptane-ethyl acetate by raising polarity to yield 62 fractions of 350 mL. Using TLC, similar fractions were collected together. During the elution with a concentration of heptane-ethyl acetate (1:100) solvent system, a white solid was isolated and monitored as a single spot on the TLC. The isolated compound was further purified by Sephadex LH-20 to yield 70 mg of a white amorphous solid with a melting point (m.p.) of 271–275 °C. In accordance with the spectral data, the structure of the kaempferol acetylated glycoside was proposed (Figure 1). Spectral data are presented in Table 1.

**Figure 2:**
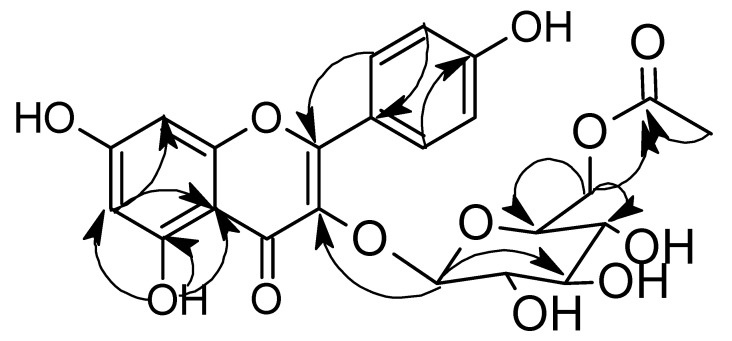
Main HMBC relations of K3G-A.

The structure of K3G-A was identified by ^1^H, ^13^C NMR (Table 1), and 2D NMR stereoscopy (Figure 2) The ^1^H and ^13^C spectral data (Table 1) indicated the presence of the kaempferol flavonoid through the identification of 15 carbon signals containing the characteristic di para-substituted benzene ring (AA′-BB′ pattern) for (C-2′, C-6′, C-3′, C-5′), the conjugated upfield ester carbonyl of C-2. The ^1^H confirmed the characteristic di para-substituted benzene ring and declared the meta conjugated protons of C-6 and C-8. The ^1^H of the OH at C-5 appeared clearly as a sharp singlet signal at δ12.56 because of the incorporation in intramolecular hydrogen bonding with the carbonyl of C-4. The presence of the glucose unit and acetate moiety was also clear according to their characteristic signals. The unity of the isolated compound was confirmed through the essential HMBC correlation (Figure 2) between the C-3 in the kaempferol and the anomeric proton of the glucose sugar, in addition to the vital HMBC correlation between the oxygenated methylene of glucose and the carbonyl of the acetate moiety. Finally, the spectral data were compared to the published data [37] and the isolated compound was identified to be kaempferol 3-*O*-(6″-*O*-acetyl)-glucoside.

Compound K was isolated with a solvent mixture of heptane-ethyl acetate (1:7). Its structure was determined by ^1^H and ^13^C NMR spectroscopy. Compound K was obtained as yellow crystals with an m.p. of 277–278 °C. Its molecular formula, C_15_H_10_O_6_, was confirmed by mass spectroscopy, which indicated the peak ion [M+H]^−^ *m/z* 287.

According to the ^1^H and ^13^C NMR data obtained, compound K was identified as kaempferol, which was previously isolated from some species of the *Artemisia* family and other plant species [38].

During the elution with a heptane-ethyl acetate (7:3) system, a compound was isolated, which, according to mass spectral data, was identified by ^1^H NMR as beta-sitosterol [39] (Figure 1). It is worth mentioning that compounds were reported for the first time from *Calligonum tetrapterum.*

### 2.2. Molecular Similarity 

**Figure 3:**
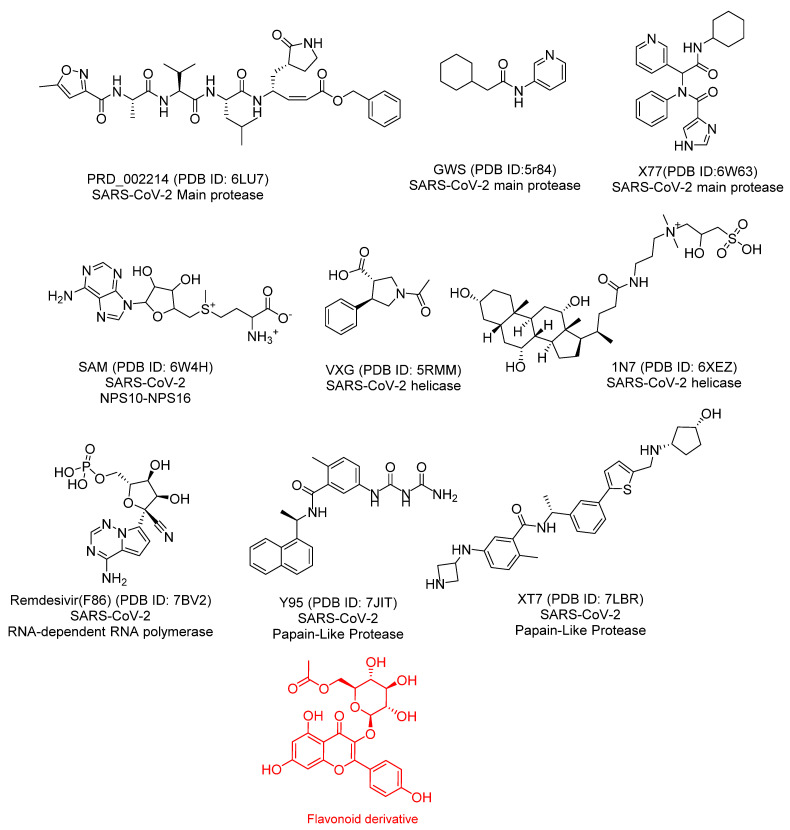
The chemical structures of the compared co-crystallized ligands of SARS-CoV-2 proteins and K3G-A (flavonoid derivative).

The co-crystallized ligand is a chemical compound that shows a great affinity to bind with a specific protein and crystallize [40]. According to the structure–activity relationship principles, if there is a compound that has a similar chemical structure to the ligand, it is expected to bind with that protein and inhibit its function [41]. Against this background, the chemical structure of K3G-A was compared with the structures of nine co-crystallized ligands of nine proteins of SARS-CoV-2 (Figure 3). The presented work aimed to examine the existence of a structural similarity that may be linked to a high degree of binding affinity.

**Figure 4:**
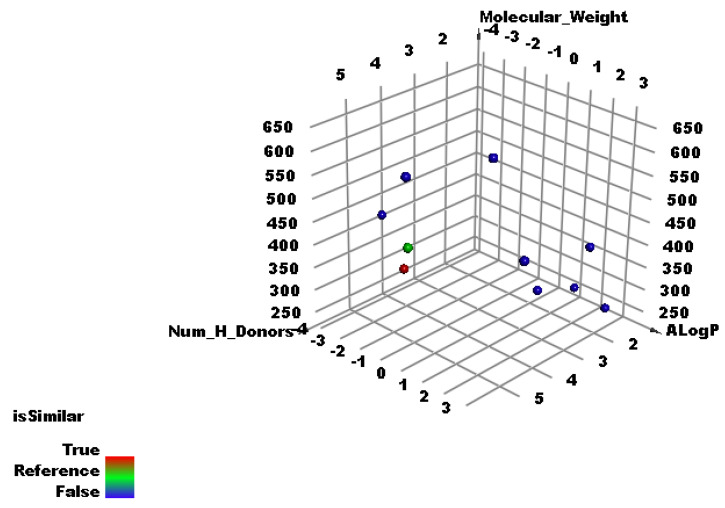
Similarity analysis results: K3G-A (green sphere) appears close to remdesivir (red sphere).

Discovery Studio software was applied to examine the subsequent molecular and structural features in K3G-A and the considered ligands. First, assessments were made of the partition coefficient, ALog p, which is the ratio of the concentration of the considered compound in the aqueous phase to its concentration in the organic phase [42], exact molecular weight (M. WT) [43], hydrogen bond acceptors (HB-A) [44], Hbond donors (HB-D) [45], rotatable bonds (R-B) [46], count of rings (R) and aromatic rings (A-R) [47], and molecular fractional polar surface area (MFPSA) [48]. The results indicated the high similarity level between K3G-A and remdesivir, F86, the co-crystallized ligand of RdRp (PDB ID: 7BV2). As shown in Figure 4, K3G-A (green sphere) appears close to remdesivir (red sphere), indicating the high similarity in the examined properties.

Table 2 demonstrates the values of the examined structural features in both K3G-A and remdesivir, showing a good minimum distance of 0.8.

### 2.3. DFT Studies

The DFT parameters were studied for K3G-A and remdesivir using Discovery Studio to investigate the similarity degree between the two molecules in terms of the levels of molecular orbitals and molecular electrostatic potential maps (MEPs) [49,50]. The similarity in the orbital and EPM levels indicates the resemblance in the activity of interaction against the same target.

#### 2.3.1. Molecular Orbital Analysis

**Figure 5:**
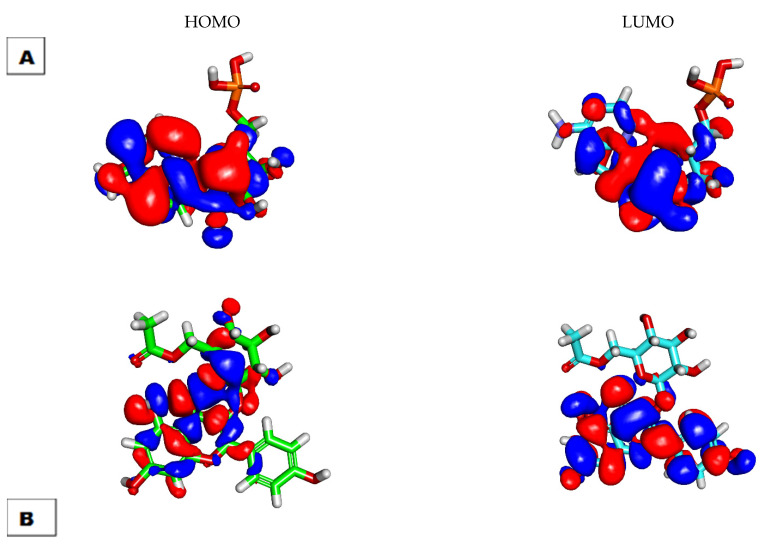
Spatial distribution remdesivir (**A**) and K3G-A (**B**).

K3G-A presented a total energy value of −1777.8125, which is higher than that of remdesivir (−1595.3914 kcal/mol). This indicates a higher reactivity of K3G-A against the prospective biological target (SARS-CoV-RNA-dependent RNA polymerase) (Figure 5). K3G-A demonstrated a dipole moment value of 2.0613, which is also higher than that of remdesivir (0.8313). It is likely that, as shown in Table 3, the gap energy of the K3G-A (0.0707 kcal/mol) was higher than that of remdesivir (0.0454 kcal/mol), indicating the higher stability of K3G-A.

#### 2.3.2. Molecular Electrostatic Potential Maps (MEPs)

MEPs present the electrostatic potential of the considered molecule in a 3D form depending on the partial charges, the electronegativity, and the chemical reactivity [51]. MEPs can be utilized to assess the binding and interaction of a compound to a specific protein [52]. In MEPs, the electronegative atoms, which are expected to be H-bond acceptors, are stained red, whereas atoms of poor electrons, which are expected to be H-bond donors, are stained blue. Finally, neutral atoms, which are expected to form hydrophobic interactions, are stained green to yellow [53].

**Figure 6:**
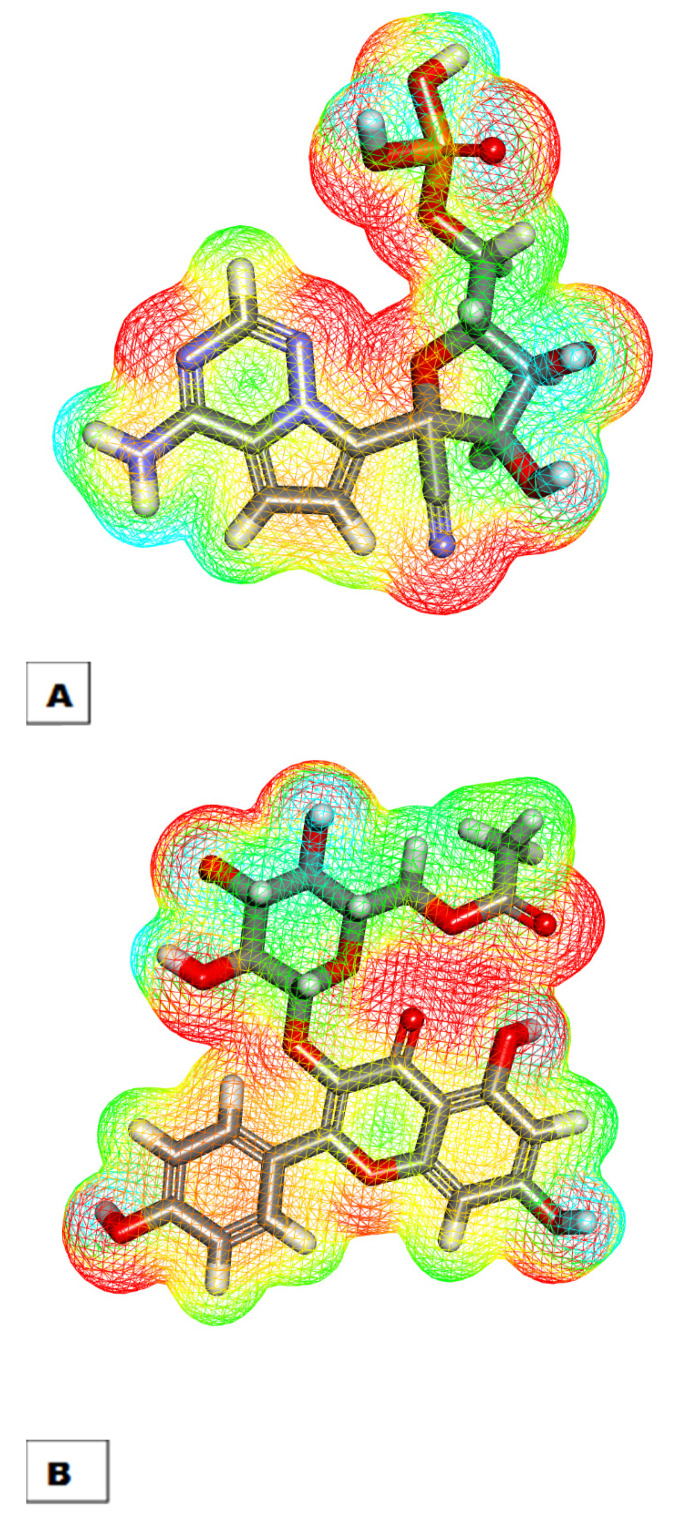
Molecular electrostatic potential maps of remdesivir (**A**) and K3G-A (**B**).

The MEPs of K3G-A and remdesivir are illustrated in Figure 6A,B, respectively. Investigating these figures indicates that remdesivir has ten red-colored patches and seven blue-colored patches. In addition, there is a yellow-colored patch on the aromatic moiety, indicating a high possibility of hydrophobic interaction. For kaempferol 3-*O*-(6″-*O*-acetyl)-glucoside, ten red-colored patches and six blue-colored patches can be observed. Furthermore, there is a yellow-colored patch on K3G-A moiety, indicating a high possibility of hydrophobic interaction. These findings indicate the high similarity of the electronic structure of K3G-A and remdesivir. In addition, it suggests the high possibility of K3G-A interacting with the target receptor.

### 2.4. Docking Studies

The presented results indicate a great degree of similarity between K3G-A and remdesivir, the ligand of RdRp (PDB ID: 7BV2). According to these outputs, K3G-A is expected to bind correctly to RdRp (PDB ID: 7BV2). To examine this claim, docking studies were conducted, in which the crystal structure of RdRp (PDB ID: 7BV2) was utilized. Remdesivir was used as a reference. The free energy of binding (∆G) on a site with the binding mode against RdRp was considered the main factor distinguishing between the docked poses.

Confirmation of the docking analysis was tested by carrying out the docking for remdesivir only in the active pocket of RdRp. The produced RMSD value between the docked pose and the apo ligand was 0.92 °A. This low RMSD value indicates the accuracy of the utilized docking protocol (Figure S1).

Remdesivir demonstrated a binding energy value of −23.57 Kcal/mol against the active site of RdRp. It showed five H-bonds, six hydrophobic interactions, and two electrostatic interactions. In detail, the heteroaromatic system (4-aminopyrrolo[2,1-f][1,2,4]triazin) formed two H-bonds with Urd10. Furthermore, it formed six hydrophobic interactions with Urd20, Ade11, and Ser682. Moreover, the aromatic system formed an electrostatic attraction with Arg555.

The sugar moiety was incorporated in two H-bonds with Asp623 and Ser759. The phosphate derivative moiety exhibited an H-bond Arg555, and two electrostatic interactions with Asp760 and Arg555 (Figure S2).

**Figure 7:**
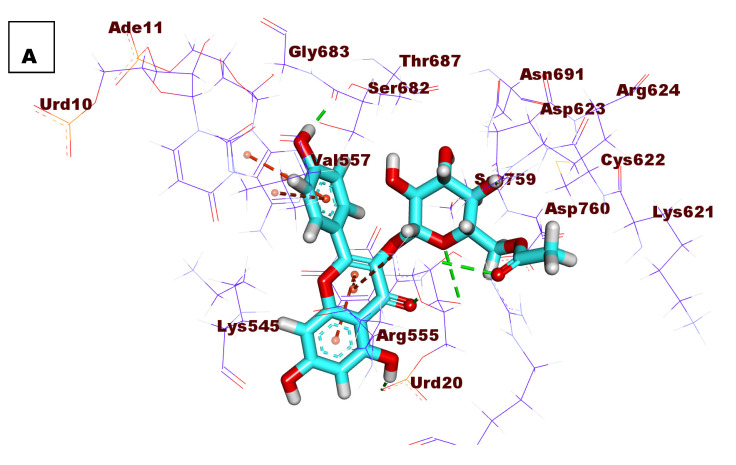

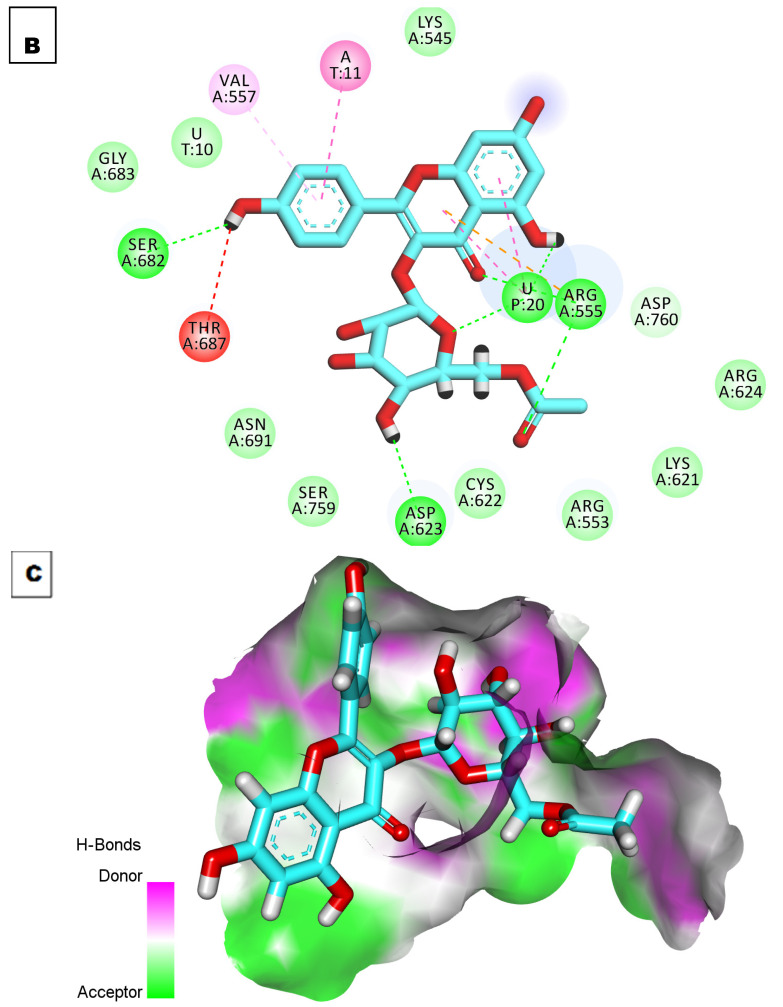
(**A**) 3D image (**B**) 2D image, and (**C**) surface mapping of K3G-A docked into the active site of RdRp.

K3G-A showed a binding free energy of −27.43 kcal/mol, which was higher than that of remdesivir. It exhibited six H-bonds, and three hydrophobic and two electrostatic interactions. In detail, the 5,7-dihydroxy-4*H*-chromen-4-one moiety was consolidated in two H-bonds with Urd20. Furthermore, two hydrophobic interactions with Urd20 and an electrostatic interaction with Arg555 were formed. The 4-hydroxyphenyl at the 2-position of chromene moiety was consolidated in two hydrophobic interactions with Ade11 and Val557, and an H-bond with Ser682 and an electrostatic interaction with Thr687. The acylated sugar moiety was incorporated in three H-bonds with Asp623, Arg555, and Urd20 (Figure 7).

### 2.5. In Silico ADMET Analysis

**Figure 8:**
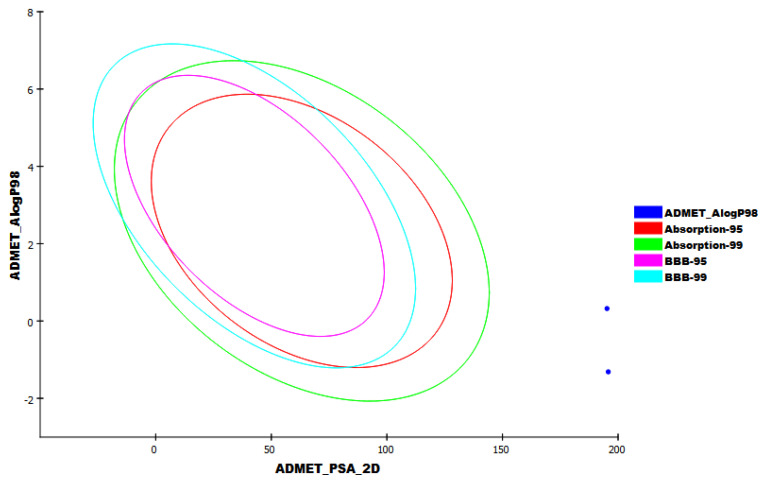
The ADMET study of K3G-A and remdesivir.

The in silico ADMET was computed for K3G-A to investigate its likeness to be used as a drug compared to remdesivir. The experiment was conducted using Discovery Studio software. The outputted data (Table 4 and Figure 8) indicate the general likeness of K3G-A to be administrated as a drug, having great similarity to remdesivir in the five examined parameters. The obtained results outline the expected safety of K3G-A in comparison to remdesivir regarding the CNS and liver toxicity.

### 2.6. In Silico Toxicity Studies

The expected toxicity of K3G-A against seven toxicity models was computed in Discovery Studio. The outputs are listed in Table 5 and indicate the general safety of K3G-A, which exhibited very safe results compared to remdesivir in the applied acute and chronic models. Consequently, few possible side effects are expected.

### 2.7. MD Simulations Studies

The main advantage of molecular dynamics (MD) simulation studies is the capability to compute the flexibility of any protein–compound complex. Thus, MD can accurately determine both thermodynamics and kinetics variations that occur through the protein–compound binding (52). To validate the binding and explore the thermodynamic properties of K3G-A against RdRp, MD simulations were conducted.

**Figure 9:**
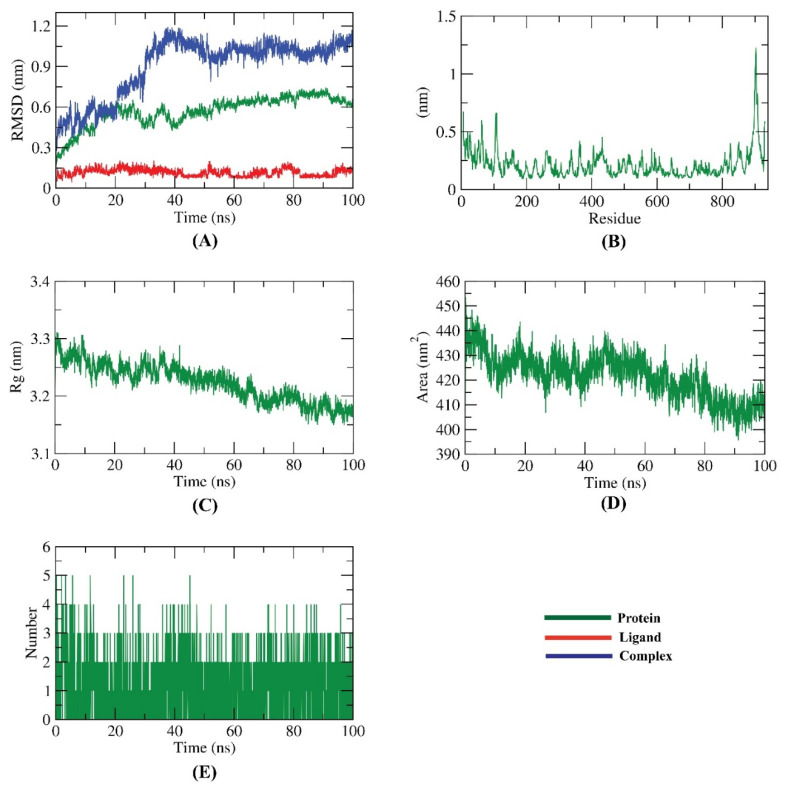
Molecular dynamics simulation results: (**A**) RMSD values, (**B**) RMSF, (**C**) R_g_, (**D**) SASA, (**E**) H-bonding of the RdRp-K3G-A complex.

The dynamic variations in atoms and conformational modifications of backbone atoms of the RdRp-K3G-A complex were calculated by RMSD to examine their stability after bonding. Although the complex fluctuated until 40 ns~, it stabilized later, at the end of the study (Figure 9A). The flexibility of each residue of the considered complex was predicted in terms of RMSF to explore the region of the RdRp that fluctuated through the simulations. The obtained results (Figure 9B) indicate that the binding of K3G-A makes the RdRp- slightly flexible in 840–860 residue areas. The compactness of the RdRp-K3G-A complex was predicted by the examination of the radius of gyration (R_g_). The R_g_ of the RdRp-K3G-A complex (Figure 9C) exhibited lower values than those at the starting time, which indicates the great stability of the complex. The interaction between the RdRp-K3G-A complex and the encompassing solvents was computed by the solvent accessible surface area (SASA) over the simulation period. SASA indicates the extent of the conformational changes that appeared during the bonding. Fortunately, the RdRp-K3G-A complex featured a decrease in the surface area, which was indicated by lower SASA values than those at the starting time of the study (Figure 9D). Hydrogen bonding among the RdRp-K3G-A complex was examined. As shown in Figure 9E, the highest number of conformations of RdRp- formed up to four H-bonds with kaempferol 3-*O*-(6″-*O*-acetyl)-glucoside.

## 3. Experimental

### 3.1. Isolation of Compounds

A total of 1.1 kg of *Calligonum tetrapterum* areal parts was collected and extracted. Successive several chromatographic techniques led to the isolation and identification of three compounds. Details are reported in the Supplementary Materials.

Kaempferol 3-*O*-(6″-*O*-acetyl)-glucoside **K3G-A**;

White amorphous crystals with m.p. 271-275 °C; UV max (ACN-H_2_O) 263, 295 (shoulder) and 350 nm; ^1^H and ^13^C NMR (DMSO-d_6_) (see Table 1). More details are reported in the Supplementary Materials.

Kaempferol, **K**:

Isolated by elution with a heptane-ethyl acetate system (1:7); Yellow crystals, m.p. 277–278 °C (lit. 272–275 °C); Mass spectra: [M+H]^−^ c *m/z* 287. UV max (ACN-H_2_O) 270, 294 (shoulder) and 360 nm;

β-Sitosterol β-S:

Isolated by elution with a heptane-ethyl acetate system (7:3). White crystals, m.p. 140–145 °C; Mass spectra: [M+H]^−^ c *m/z* 415

### 3.2. Molecular Similarity

Molecular similarity of K3G-A against nine co-crystallized ligands of SARS-CoV-2 proteins was determined by Discovery Studio 4.0 [54,55] (see Supplementary Materials).

### 3.3. DFT

The DFT parameters were computed for K3G-A and remdesivir using Discovery Studio 4.0 software [56] (see Supplementary Materials).

### 3.4. Docking Studies

A docking investigation was conducted for K3G-A and remdesivir using MOE-2014 software. The outputs of the docking were visualized by Discovery Studio 4.0 software [57,58,59] (see Supplementary Materials).

### 3.5. ADMET

ADMET descriptors of K3G-A were estimated using Discovery Studio 4.0. [60,61] (see Supplementary Materials).

### 3.6. Toxicity Studies

Seven toxicity parameters of K3G-A and remdesivir were estimated using Discovery Studio 4.0 [62,63,64] (see Supplementary Materials).

### 3.7. Molecular Dynamics Simulations

The K3G-A-RdRp complex was prepared by the web-based CHARMM-GUI [65,66,67] interface employing the CHARMM36 force field [68] and NAMD 2.13 [69] package. The TIP3P explicit solvation model was utilized (see Supplementary Materials).

## 4. Conclusions

The rare flavonol glycoside, kaempferol 3-*O*-(6″-*O*-acetyl)-glucoside, K3G-A, was isolated from the aerial parts of *Calligonum tetrapterum* for the first time. K3G-A exhibited promising in silico inhibitory potential of K3G-A against SARS-CoV-2 RdRp. The molecular fingerprints and structural similarity studies indicated the great similarity of K3G-A and remdesivir, the co-crystallized ligand of RdRp (PDB ID: 7BV2). A DFT study confirmed that similarity at the electronic and orbital levels. The binding of K3G-A against RdRp was confirmed by molecular docking studies, in addition to several MD studies at 100 ns. Additionally, in silico ADMET and toxicity revealed the safety and the drug-likeness of K3G-A. In addition to K3G-A, two other metabolites were isolated from the same plant and identified to be kaempferol **2** and β-sitosterol **3**. The obtained data represent encouraging primary results that may be very helpful in the fight against COVID-19.

## Figures and Tables

**Table 1:** ^1^H and ^13^C spectral data of K3G-A (DMSO).

Position	δ^1^H (*J* = Hz)	δ^13^C	Position	δ^1^H (*J* = Hz)	δ^13^C
2	-	156.4	1″	5.34 d (7.3)	101.2
3	-	133.12	2″	3.20	74.1
4	-	177.4	3″	3.24	76.2
5	-	161.2	4″	3.13 t	69.8
6	6.21 d (1.8)	98.7	5″	3.30	73.9
7	-	164.3	6″ a	4.09 d	62.8
8	6.45 d (1.8)	93.7	6″ b	3.94 dd	
9	-	156.6	7″	-	169.8
10	-	103.9	8″	1.73 s	20.2
1′	-	120.8	5-OH	12.56 s	-
2′, 6′	8.00 d (8.08)	130.9			
3′, 5′	6.87 d (8.08)	115.1			
4′	-	160.0			
5′	6.87 d (8.08)	115.1			
6′	8.00 d (8.08)	130.9			

**Table 2:** Structural properties of K3G-A and remdesivir, F86.

Compound	M. WT	HB-A	HB-D	R-B	R	A-R	MFPSA	Minimum Distance
Remdesivir	371.243	11	5	4	3	2	0.612	0.811009
K3G-A	490.414	12	6	6	4	2	0.429	-

**Table 3:** Molecular orbital spatial distribution of remdesivir and K3G-A.

	Total Energy ^a^	Binding Energy ^a^	HOMO Energy ^a^	LUMO Energy ^a^	Dipole Mag	Band Gap Energy ^a^
Remdesivir	−1595.39	−6.7804	−0.2001	−0.1547	0.8313	0.0454
K3G-A	−1777.81	−11.4455	−0.1548	−0.0841	2.0613	0.0707

^a^ Unite = Ha.

**Table 4:** Predicted ADMET for K3G-A and remdesivir.

Compound	BBB Level	Sol. Level	Abs. Level	CYP2D6 Inhibition	PPB Binding
Remdesivir	V. low	Low	V. poor	Not inhibitor	lower than 90%,
K3G-A	V. low	Low	V. poor	Not inhibitor	lower than 90%,

**Table 5:** Toxicity properties for K3G-A and remdesivir.

Comp.	FDA Rat Carcinogenic Potential (Female Mice)	Carcinogenic Potential TD_50_ (in Rats) ^a^	Maximum Tolerated Dose (in Rats) ^b^	Oral LD_50_^b^ (in Rats)	Chronic LOAEL ^b^ (in Rats)	Ocular Irritation	Skin Irritation
Remdesivir	Not carcinogen	1.012	0.235	0.309	0.004	Mild	Mild
K3G-A	Not carcinogen	0.544	0.718	1.041	0.080	Moderate	None

^a^ Unit: mg/kg /day ^b^ Unit: g/kg.

## Data Availability

Data are enclosed in the Supplementary Materials.

## Supplementary Materials

The following supporting information can be downloaded at: https://www.mdpi.com/article/10.3390/plants11152072/s1. Figure S1. Superimposition of the co-crystallized and the docked pose of remdesivir, Figure S2. (A) 3D (B) 2D and (C) Surface mapping of remdesivir docked into the active site of RdRp 1D and 2D spectral data of K3G-A, methodology, and toxicity report.
