# Supplementary material for: Isolation and In Silico Inhibitory Potential against SARS-CoV-2 RNA Polymerase of the Rare Kaempferol 3-O-(6″-O-acetyl)-Glucoside from Calligonum tetrapterum
Source: Plants (Basel). 2022 Aug 8;11(15):2072. doi: 10.3390/plants11152072 (PMC9370365; doi:10.3390/plants11152072)
Supplement: Supplementary file 1 [file plants-11-02072-s001.zip › plants-1845047-supplementary.pdf]

# Isolation and In Silico Inhibitory Potential against SARS-CoV-2 RNA Polymerase of the Rare Kaempferol 3-*O*-(6''-*O*-acetyl)-Glucoside from *Calligonum tetrapterum*

**Yerlan M. Suleimen[1], Rani A. Jose [2,3], Gulnur K. Mamytbekova [4], Raigul N. Suleimen [4*], Margarita Yu. Ishmuratova [5], Wim Dehaen [2], Bshra A. Alsfouk [6], Eslam B. Elkaeed [7] Ibrahim H. Eissa [8], Ahmed M. Metwaly [9,10*]**

[1] The laboratory of Engineering Profile of NMR Spectroscopy, Sh. Ualikhanov Kokshetau University, Kokshetau 020000, Republic of Kazakhstan

[2] Molecular Design & Synthesis, Department of Chemistry, B-3001 Leuven, Belgium;

[3] Department of Chemistry, St. Dominic's College, Kanjirappally Kottayam Kerala 686512, Mahatma Gandhi University, India

[4] Department of Technical Physics, Faculty of Physics and Technology, L.N. Gumilyov Eurasian National University, 010010, Nur-Sultan, Kazakstan

[5] Department of Botany, E.A. Buketov Karaganda University, Karaganda 100024, Republic of Kazakhstan

[6] Department of Pharmaceutical Sciences, College of Pharmacy, Princess Nourah bint Abdulrahman University, P.O. Box 84428, Riyadh 11671, Saudi Arabia

[7] Department of Pharmaceutical Sciences, College of Pharmacy, AlMaarefa University, Riyadh 13713, Saudi Arabia

[7] Pharmaceutical Medicinal Chemistry & Drug Design Department, Faculty of Pharmacy (Boys), Al-Azhar University, Cairo11884, Egypt

[9] Pharmacognosy and Medicinal Plants Department, Faculty of Pharmacy (Boys), Al-Azhar University, Cairo, Egypt

[10] Biopharmaceutical Product Research Department, Genetic Engineering and Biotechnology Research Institute, City of Scientific Research and Technological Applications, Alexandria, Egypt

**Content**


| | |
|---|---|
| Method | Fingerprint studies |
| | Molecular Similarity |
| | Docking studies |
| | ADMET studies |
| | Toxicity studies |
| | Molecular dynamics |
| Spectral data | |
| Toxicity report | |

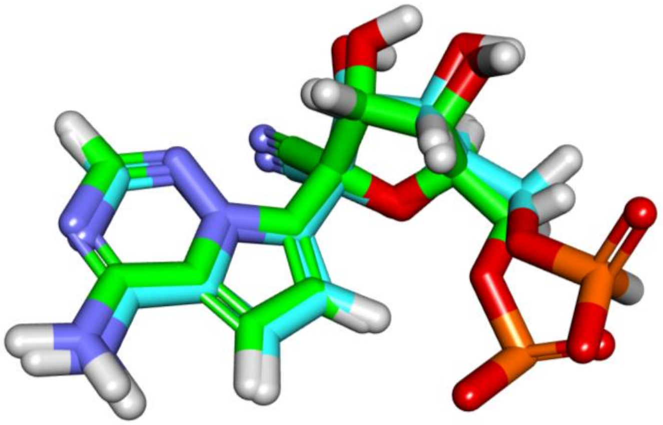

**Figure S1.** Superimposition of the co-crystallized and the docked pose of Remdesivir (pink and dark green, respectively).

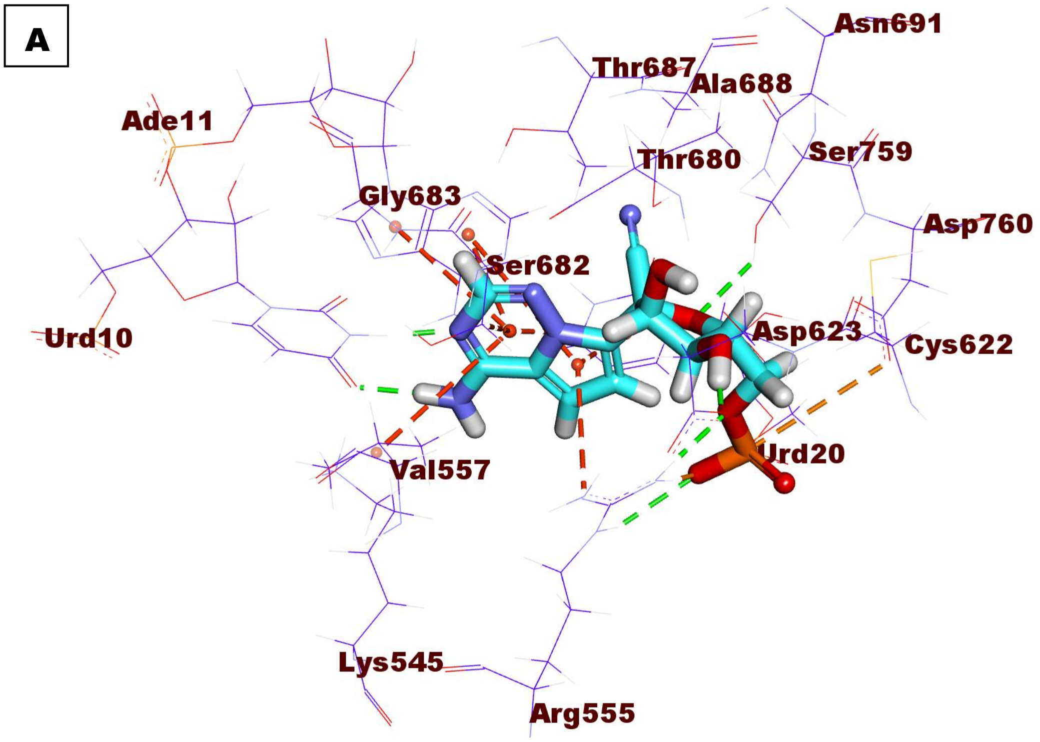

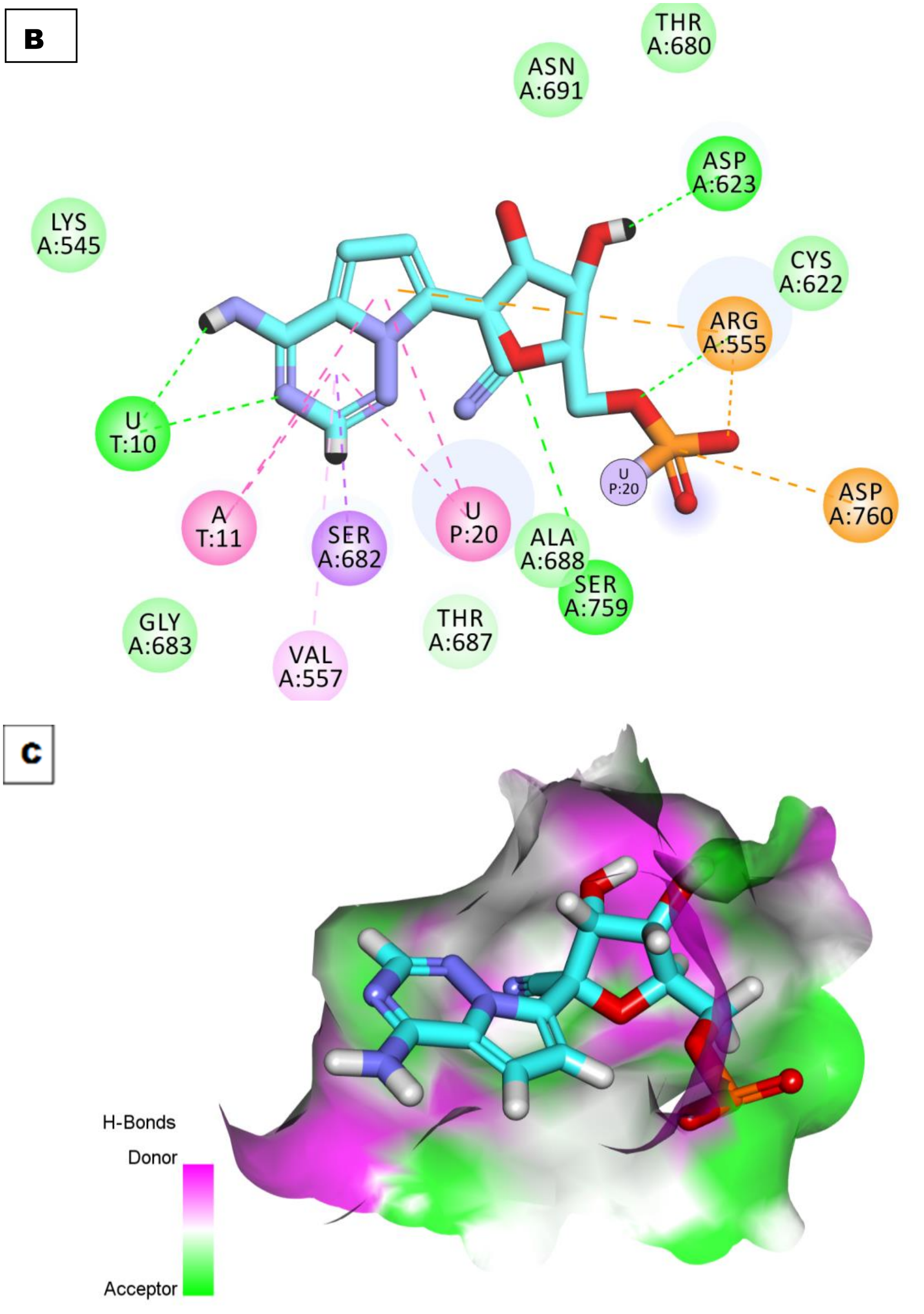


**Figure S2** : (**A**) 3D (**B**) 2D and (**C**) Surface mapping of the co-crystallized ligand docked into the active site of SARS-CoV-2 RNA-dependent RNA polymerase.

# Spectral data NMR of compound 1

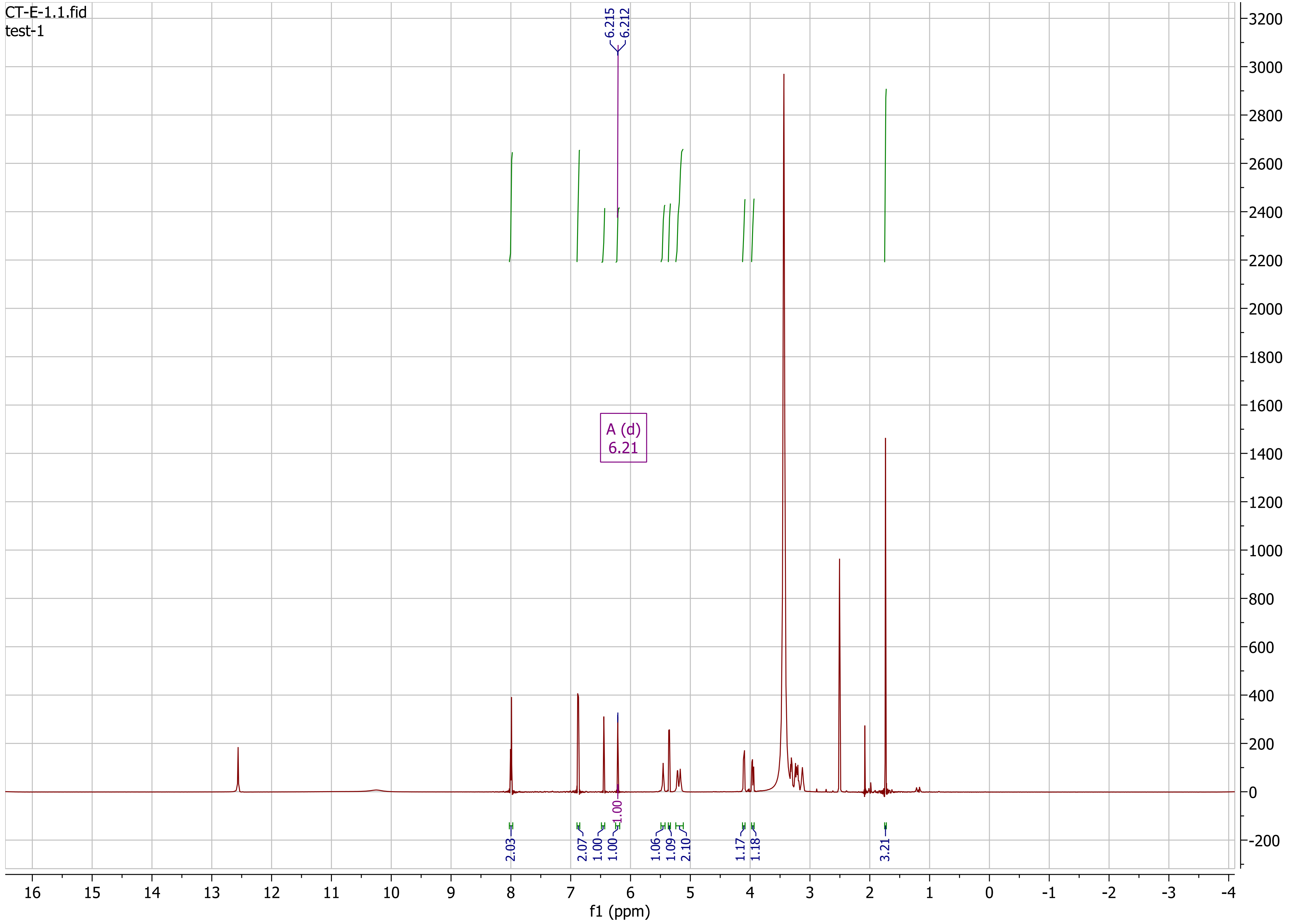

CT-E-1.1.fid
test-1
6.215
6.212
A (d)
6.21
2.03
2.07
1.00
1.00
1.06
1.09
2.10
1.17
1.18
3.21
f1 (ppm)

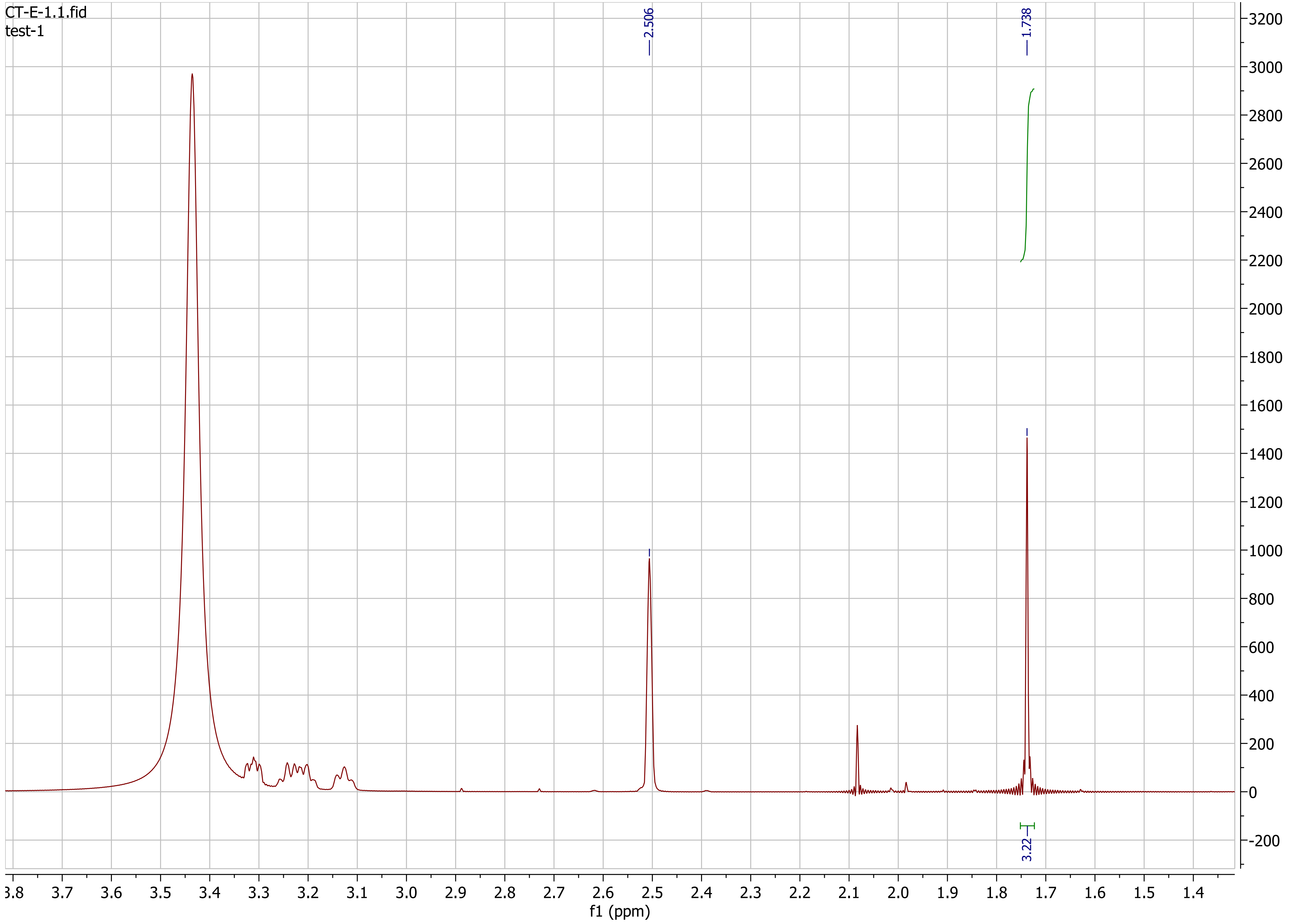

CT-E-1.1.fid
test-1
2.506
1.738
3.22
f1 (ppm)

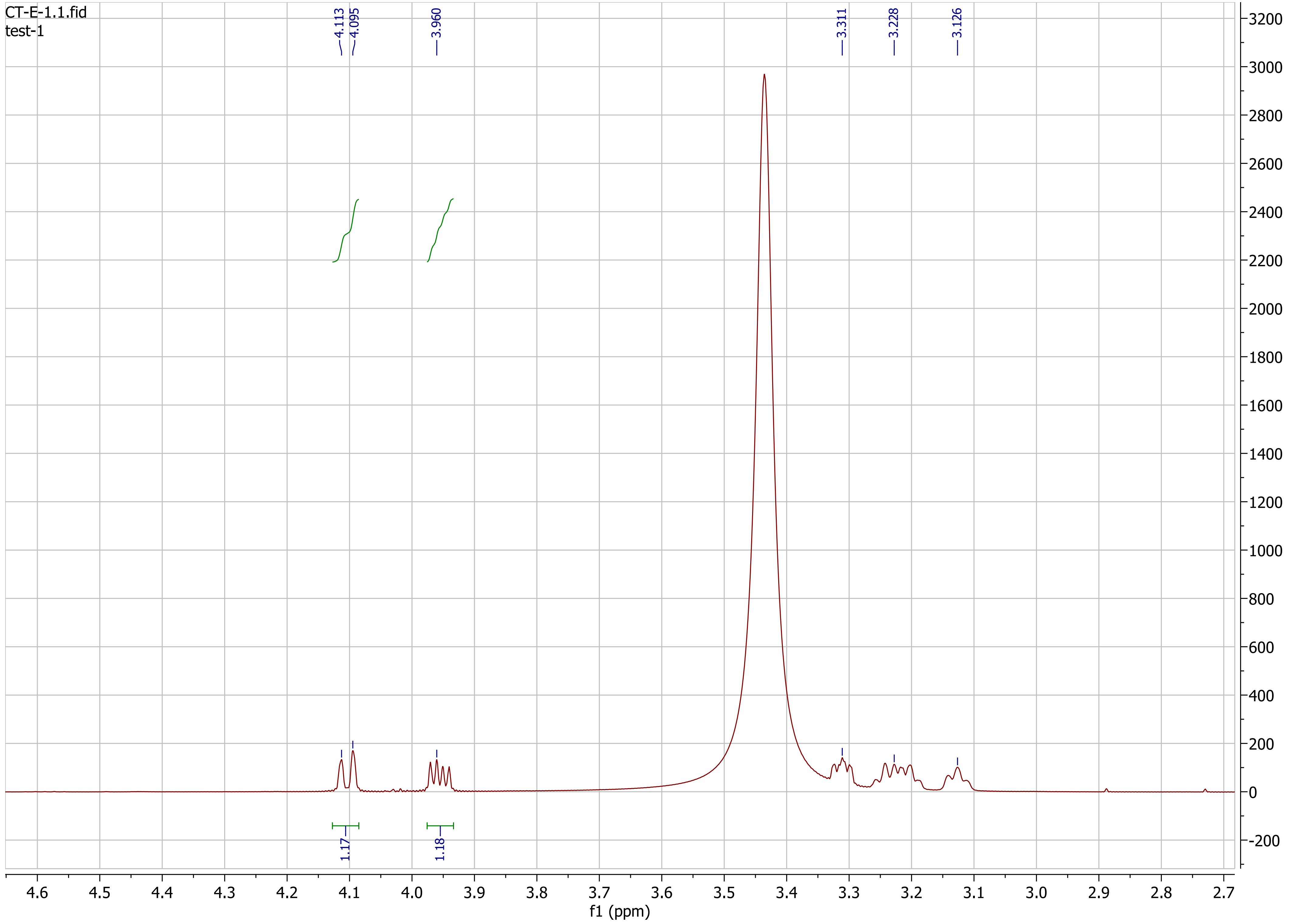
CT-E-1.1.fid
test-1
4.113
4.095
3.960
3.311
3.228
3.126
1.17
1.18
f1 (ppm)

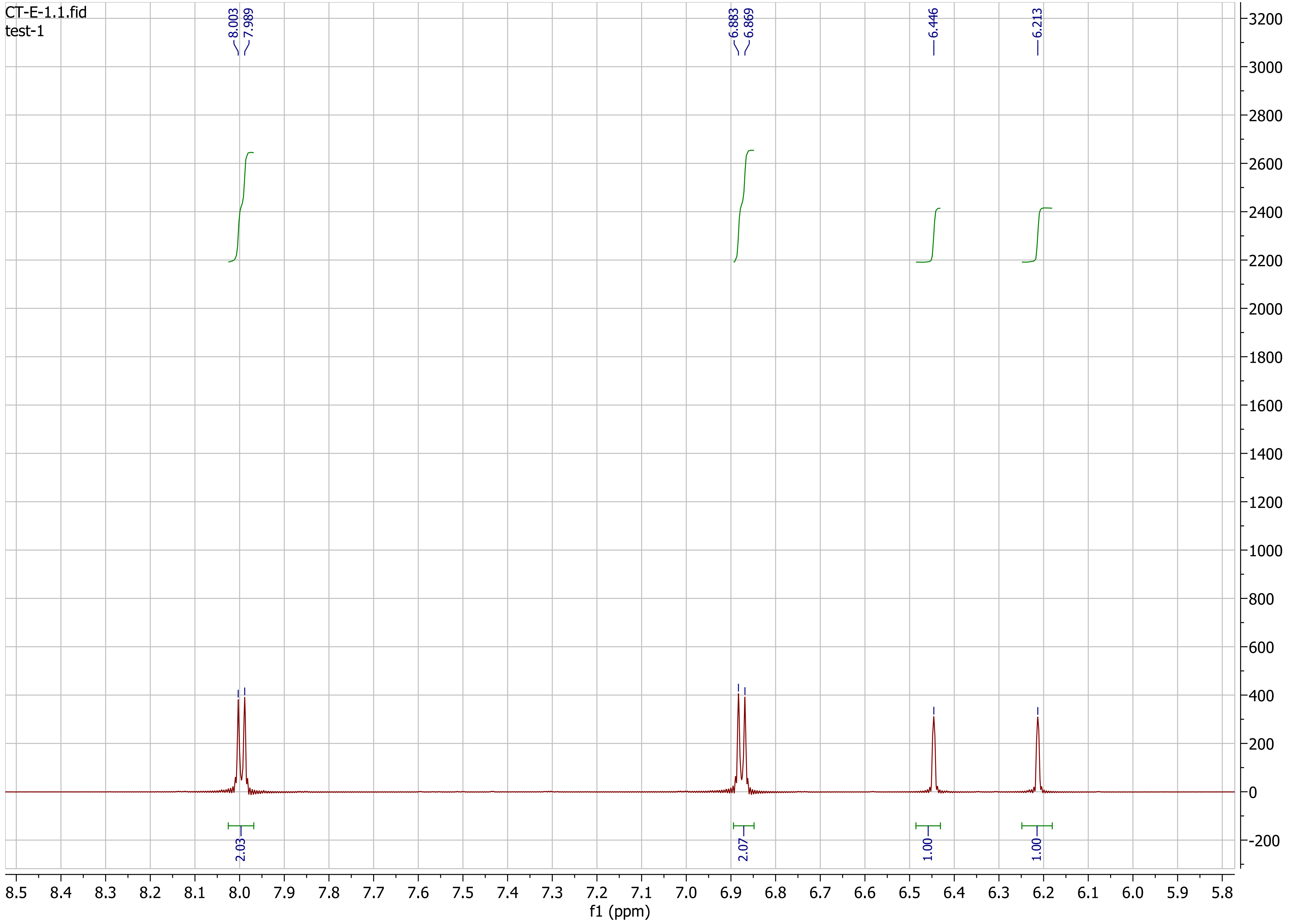

CT-E-1.1.fid
test-1
8.003
7.989
6.883
6.869
6.446
6.213
2.03
2.07
1.00
1.00
f1 (ppm)

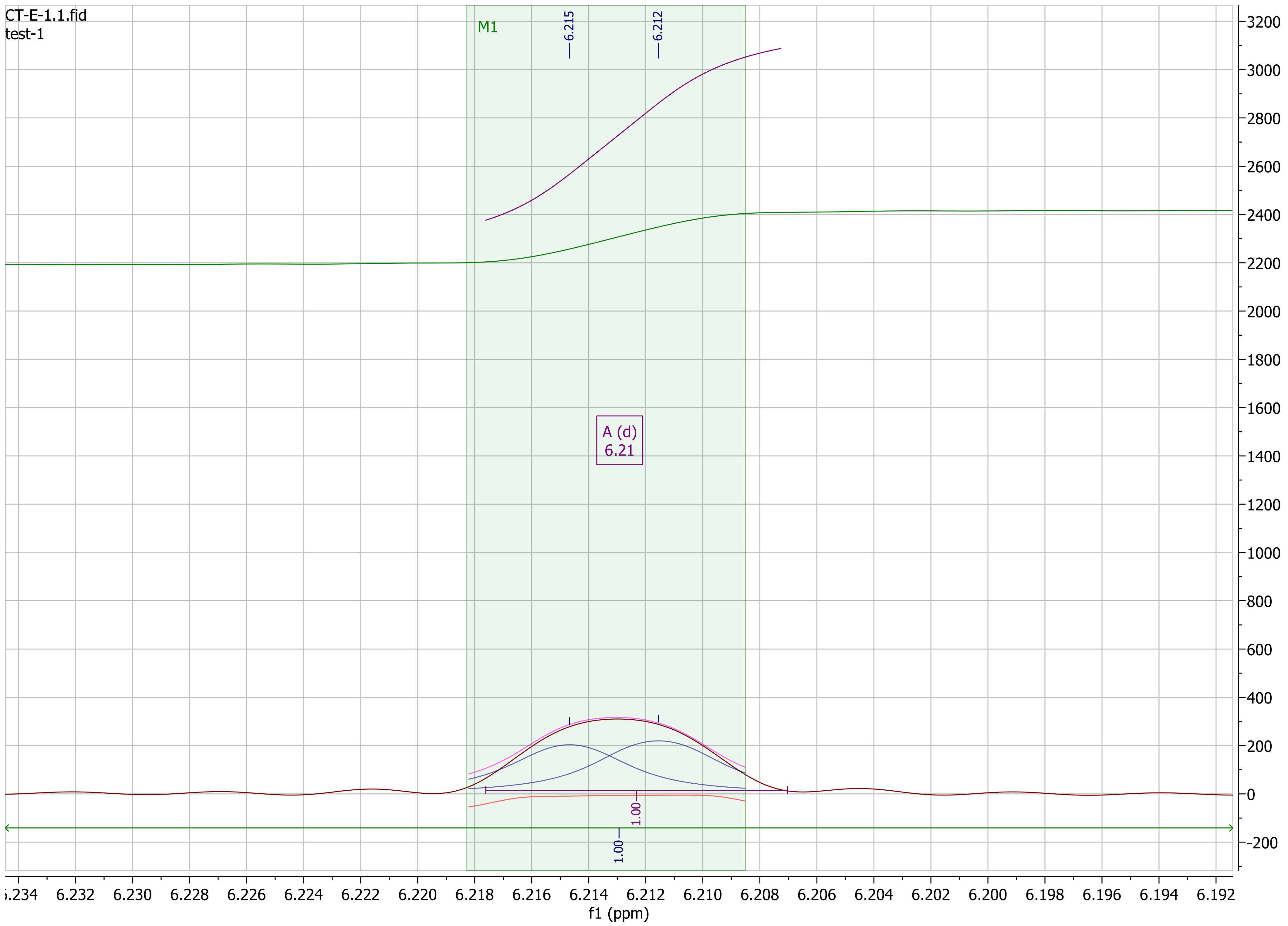
CT-E-1.1.fid
test-1
M1
6.215
6.212
A (d)
6.21
1.00
1.00
f1 (ppm)

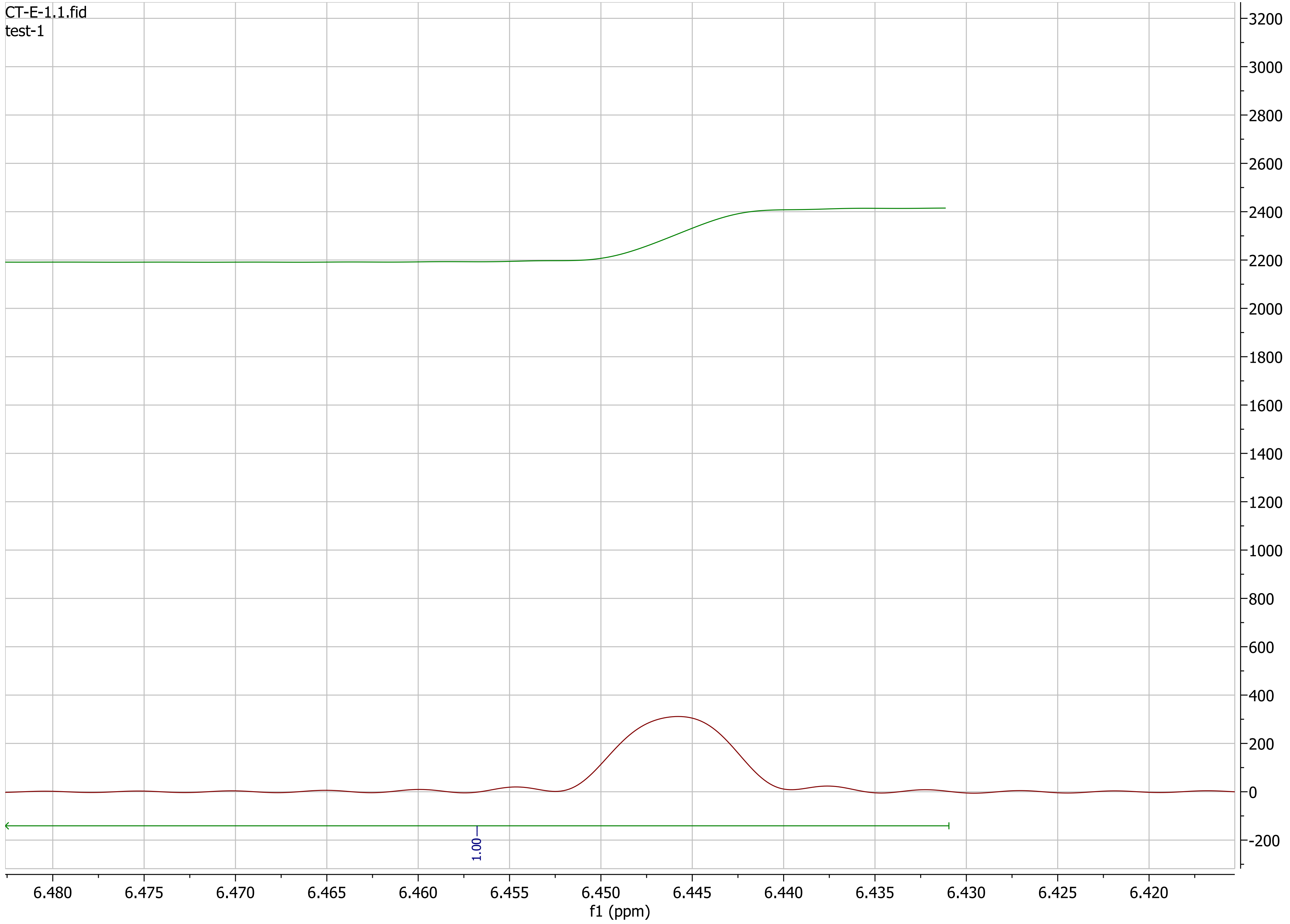
CT-E-1.1.fid
test-1
1.00
f1 (ppm)

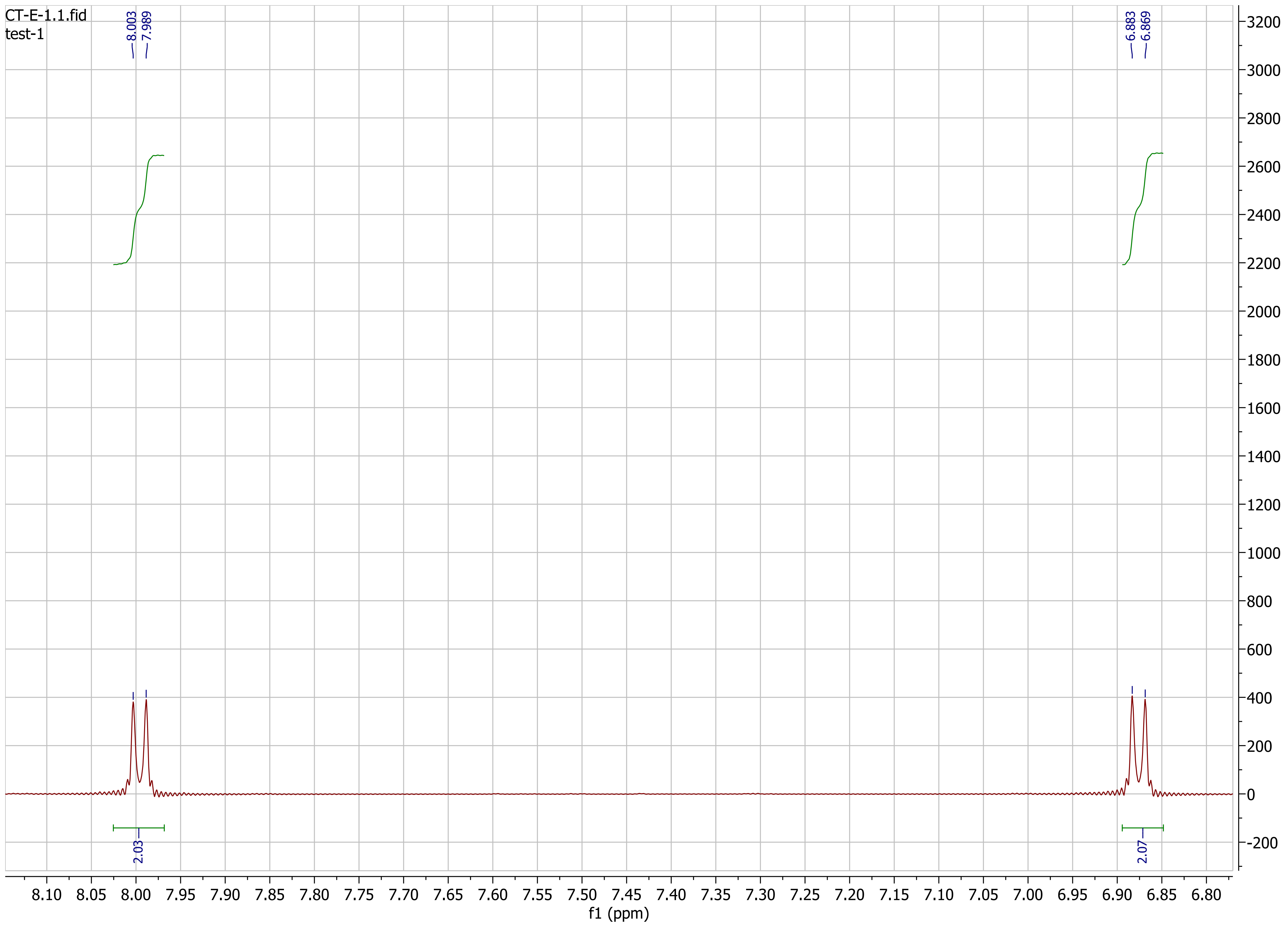

CT-E-1.1.fid
test-1
8.003
7.989
6.883
6.869
2.03
2.07
f1 (ppm)

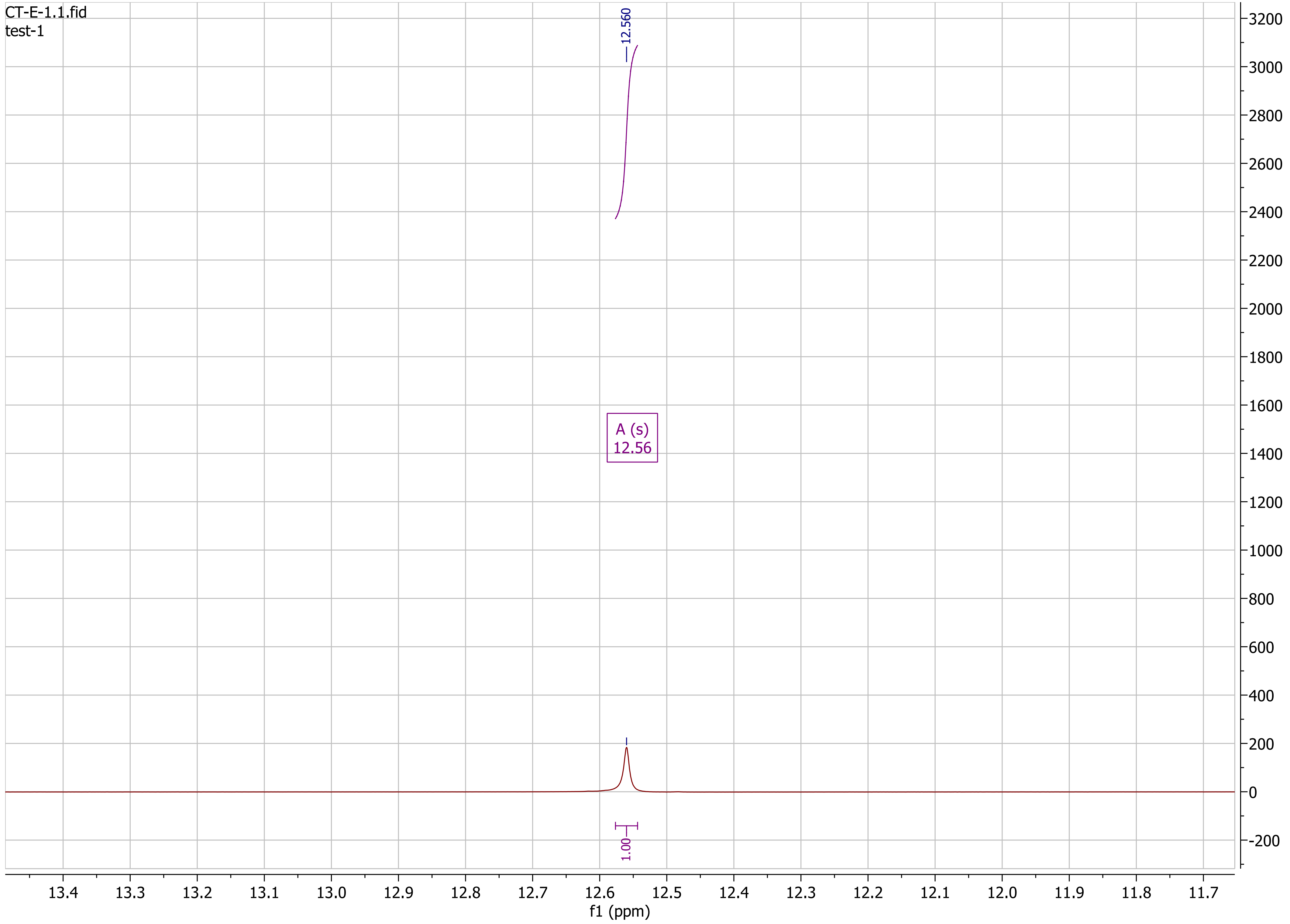

CT-E-1.1.fid
test-1
12.560
A (s)
12.56
1.00
f1 (ppm)

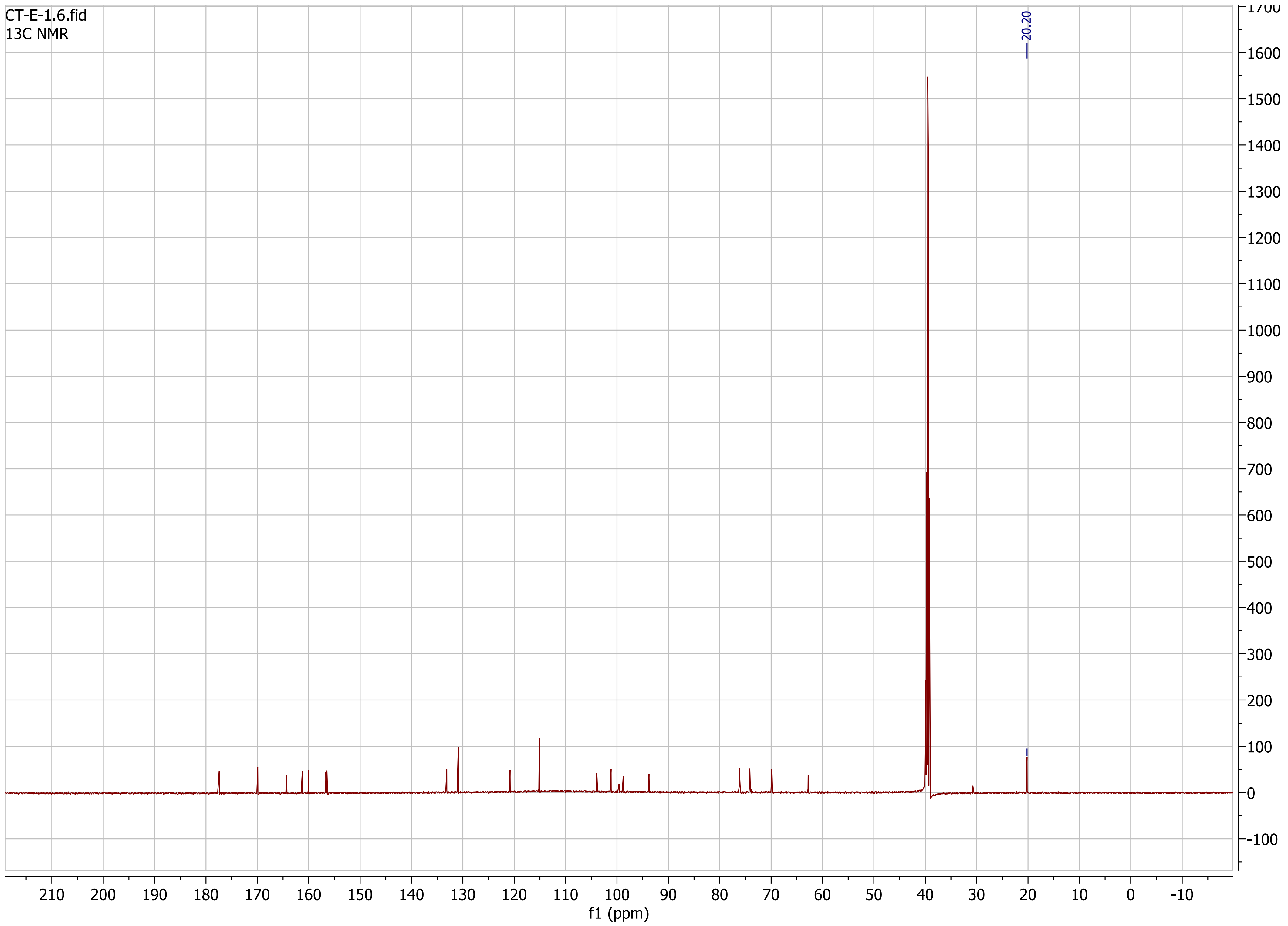
CT-E-1.6.fid
13C NMR
20.20
f1 (ppm)

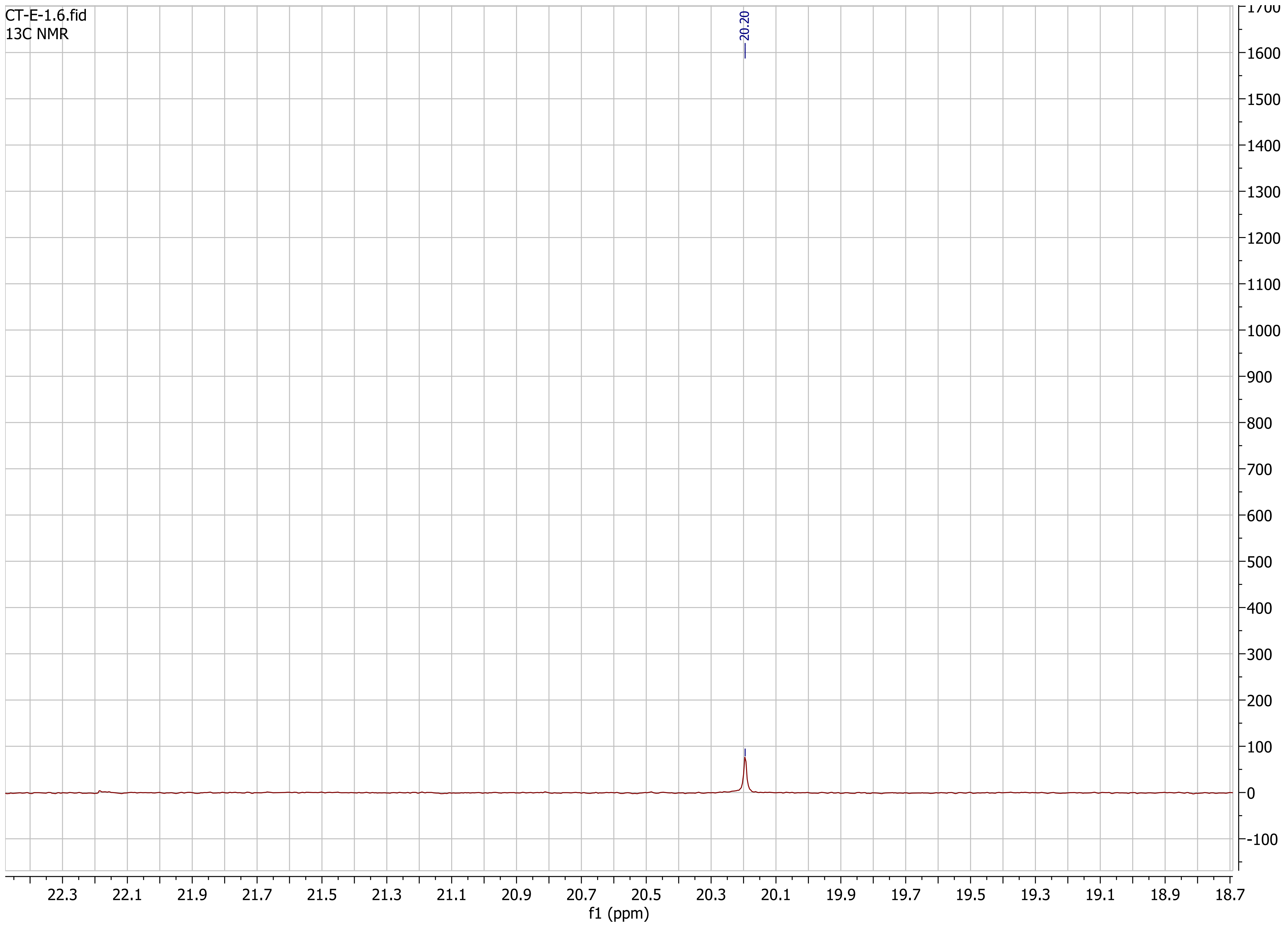
CT-E-1.6.fid
13C NMR
20.20
f1 (ppm)

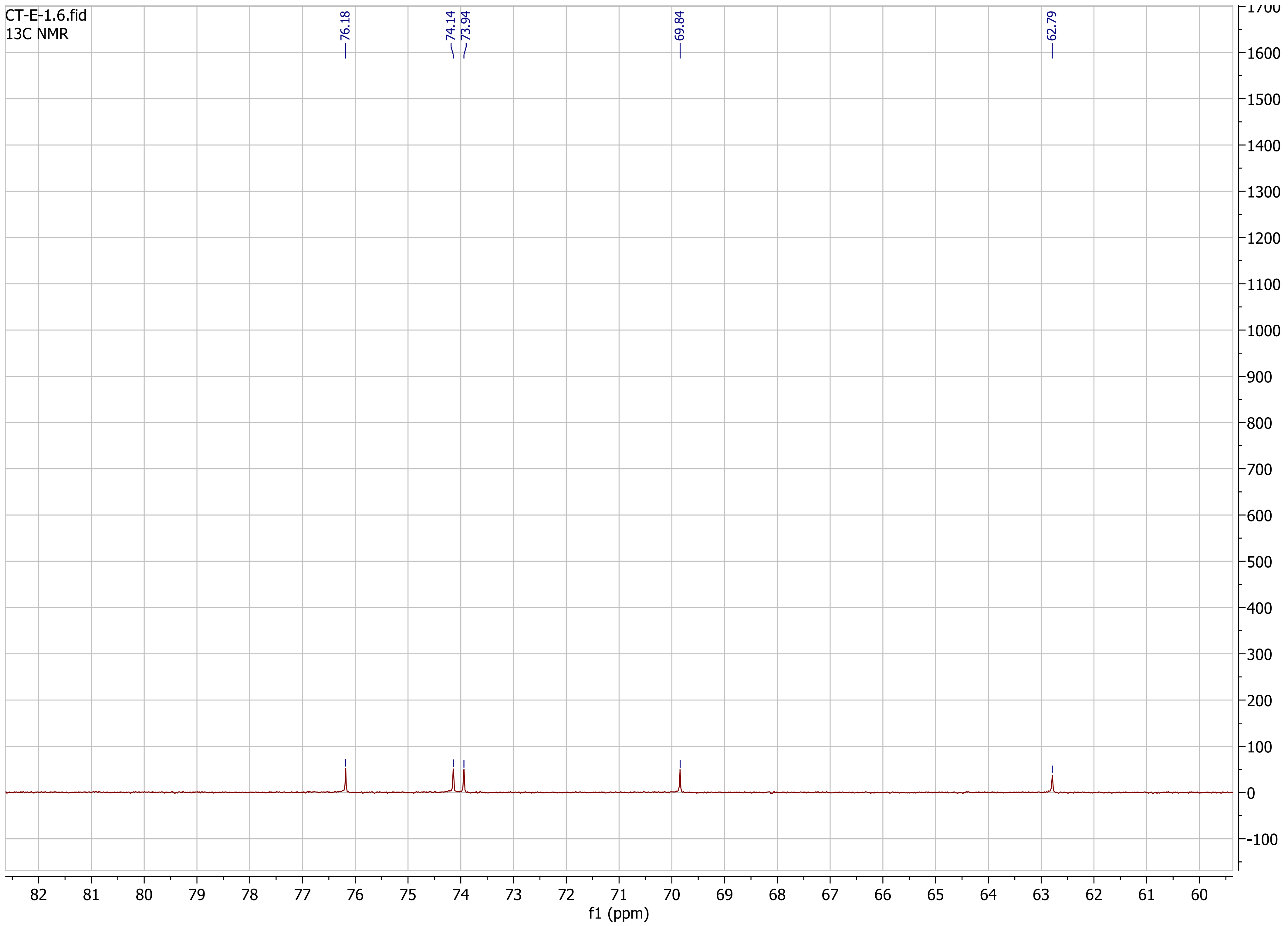

CT-E-1.6.fid
13C NMR
76.18
74.14
73.94
69.84
62.79
f1 (ppm)

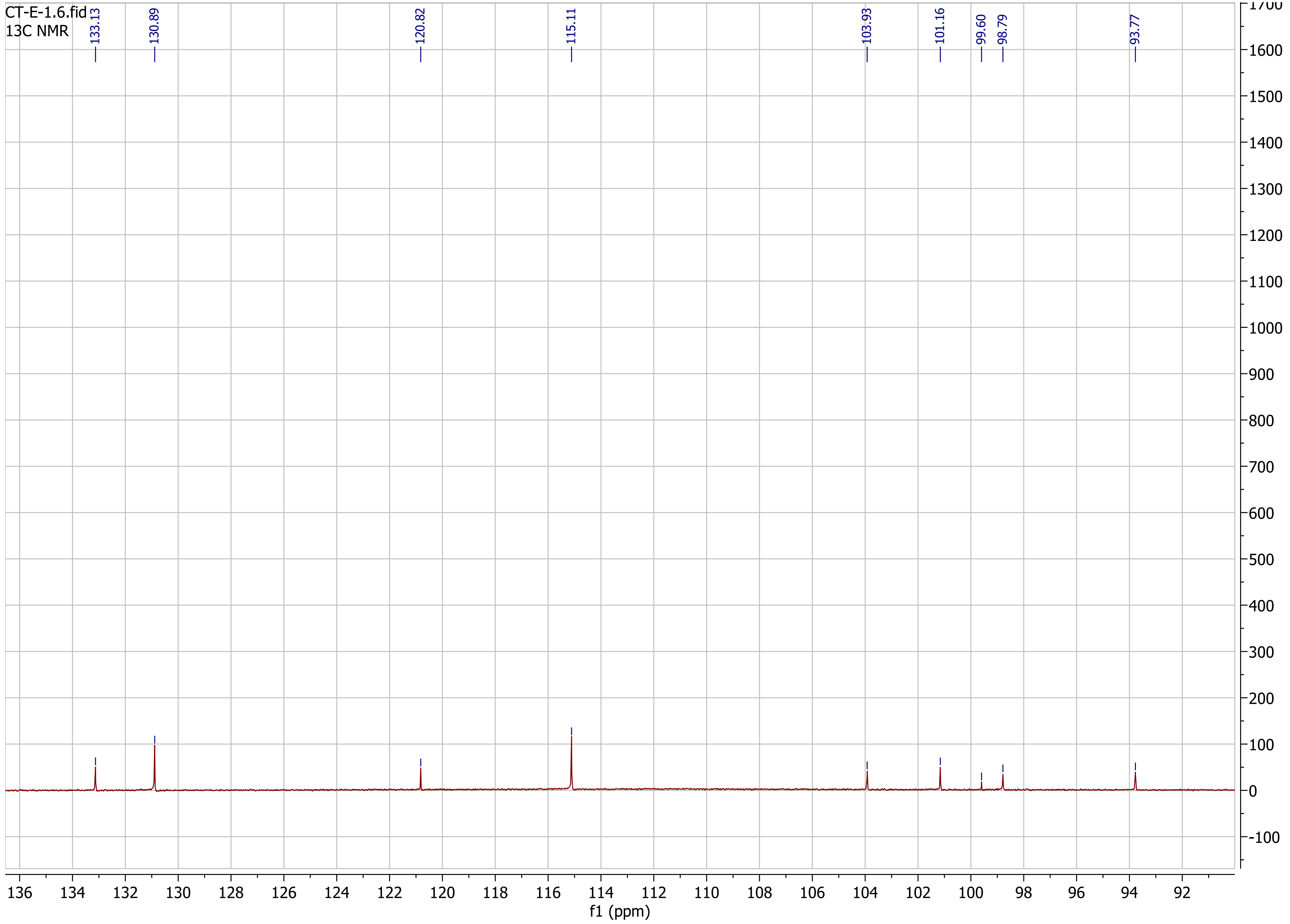
CT-E-1.6.fid
13C NMR
133.13
130.89
120.82
115.11
103.93
101.16
99.60
98.79
93.77
f1 (ppm)

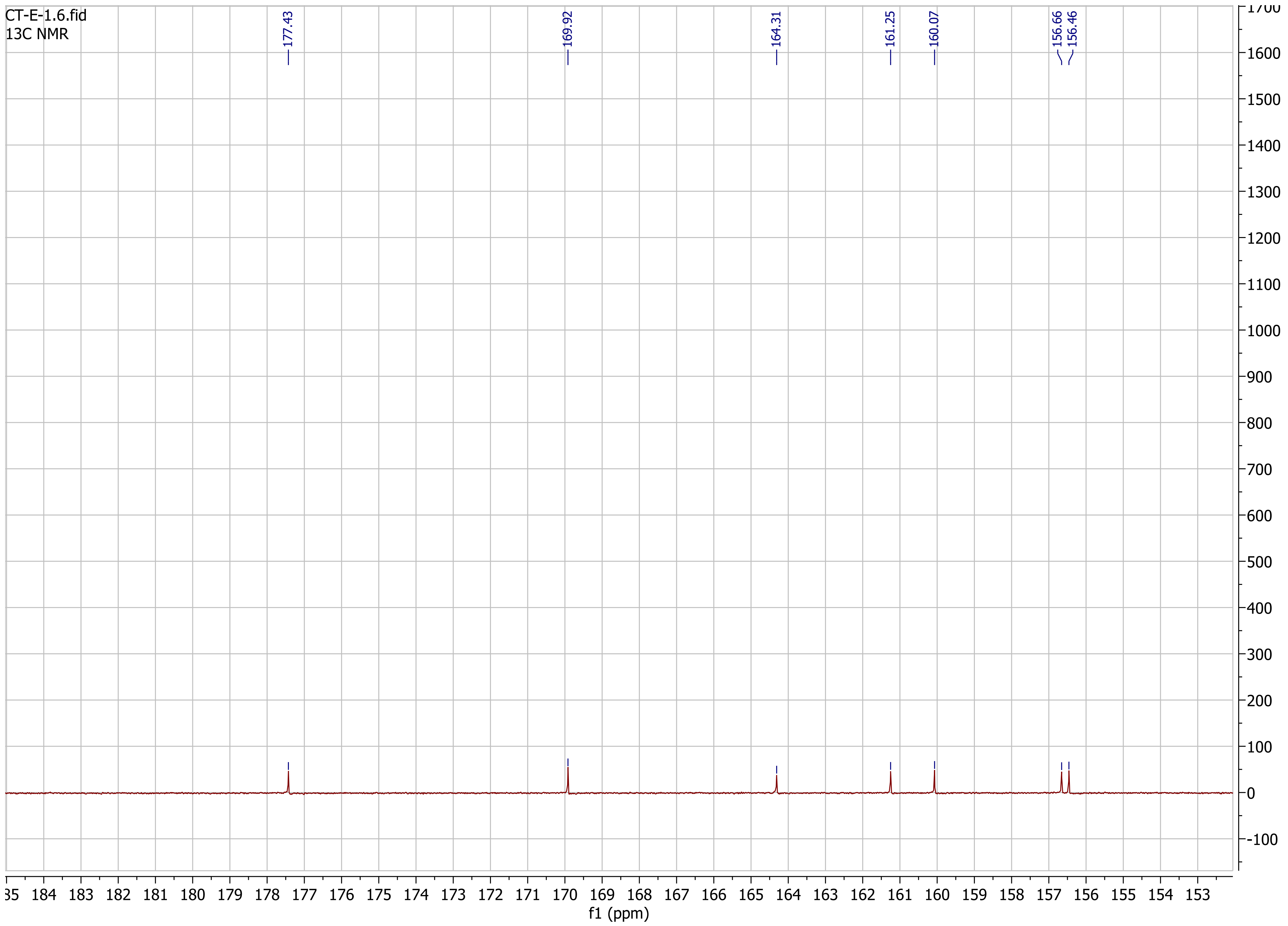
CT-E-1.6.fid
13C NMR
177.43
169.92
164.31
161.25
160.07
156.66
156.46
f1 (ppm)

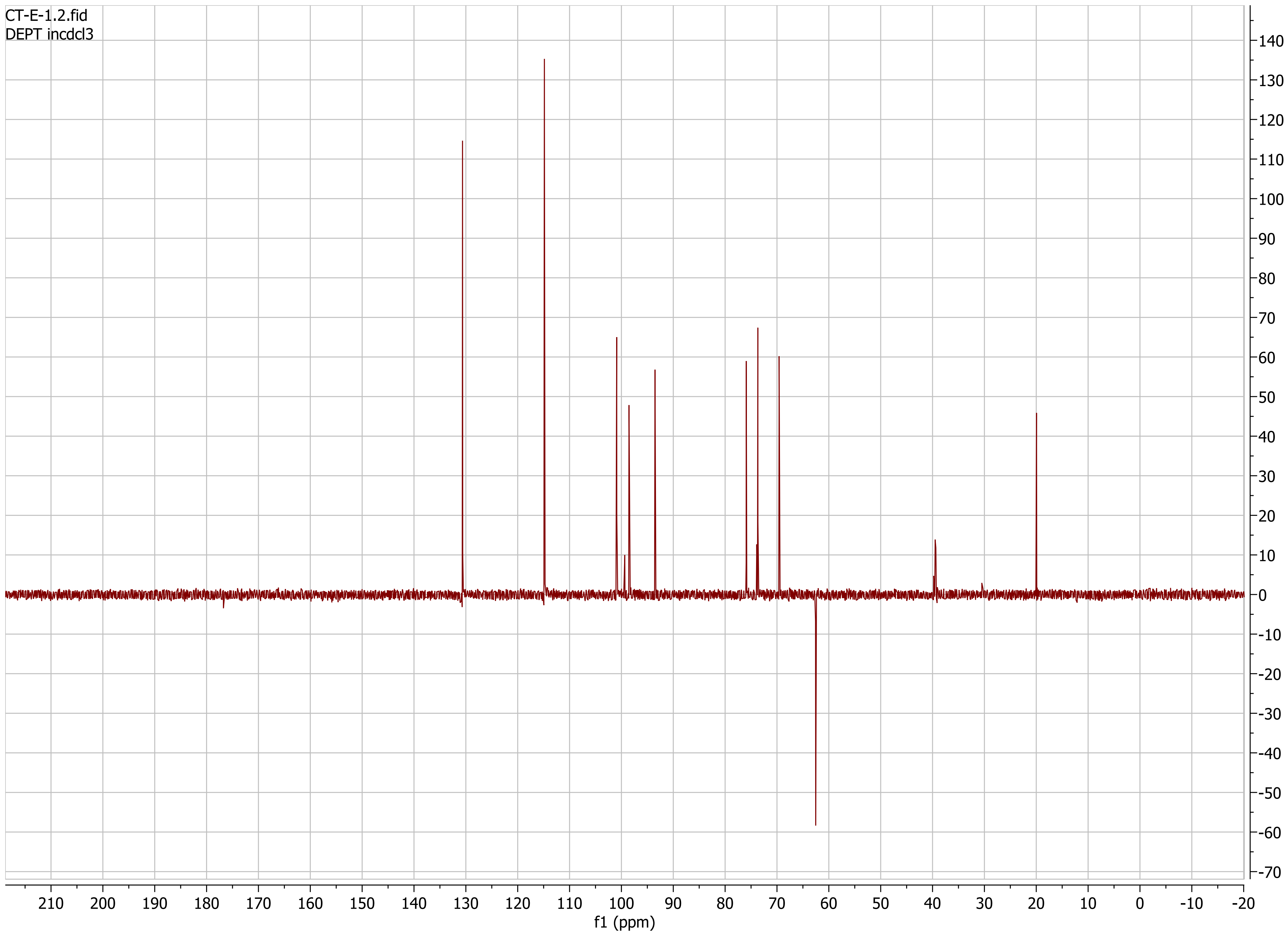

CT-E-1.2.fid
DEPT incdcl3
f1 (ppm)

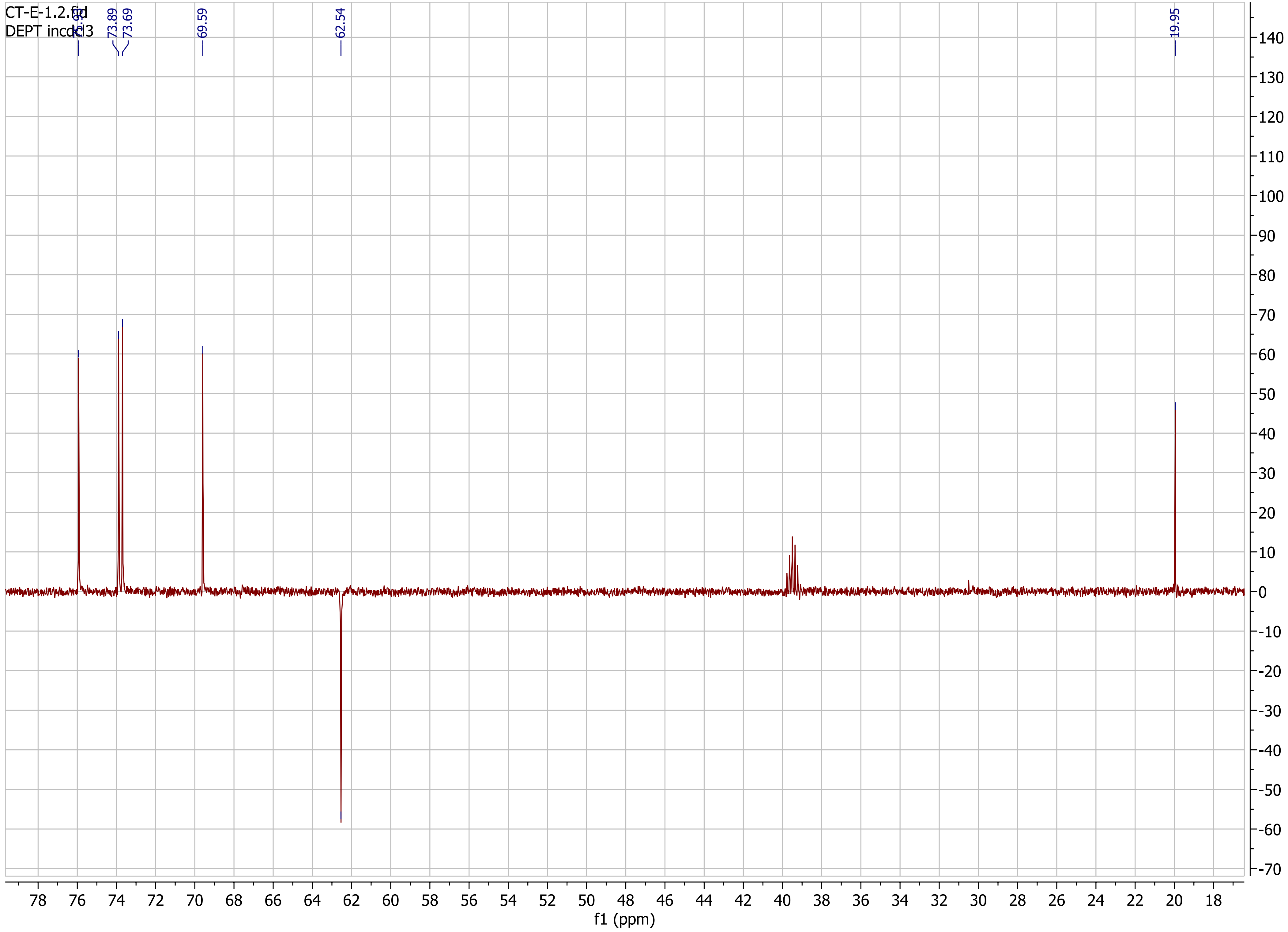
CT-E-1.2.fid
DEPT incdcl3
75.93
73.89
73.69
69.59
62.54
19.95
f1 (ppm)

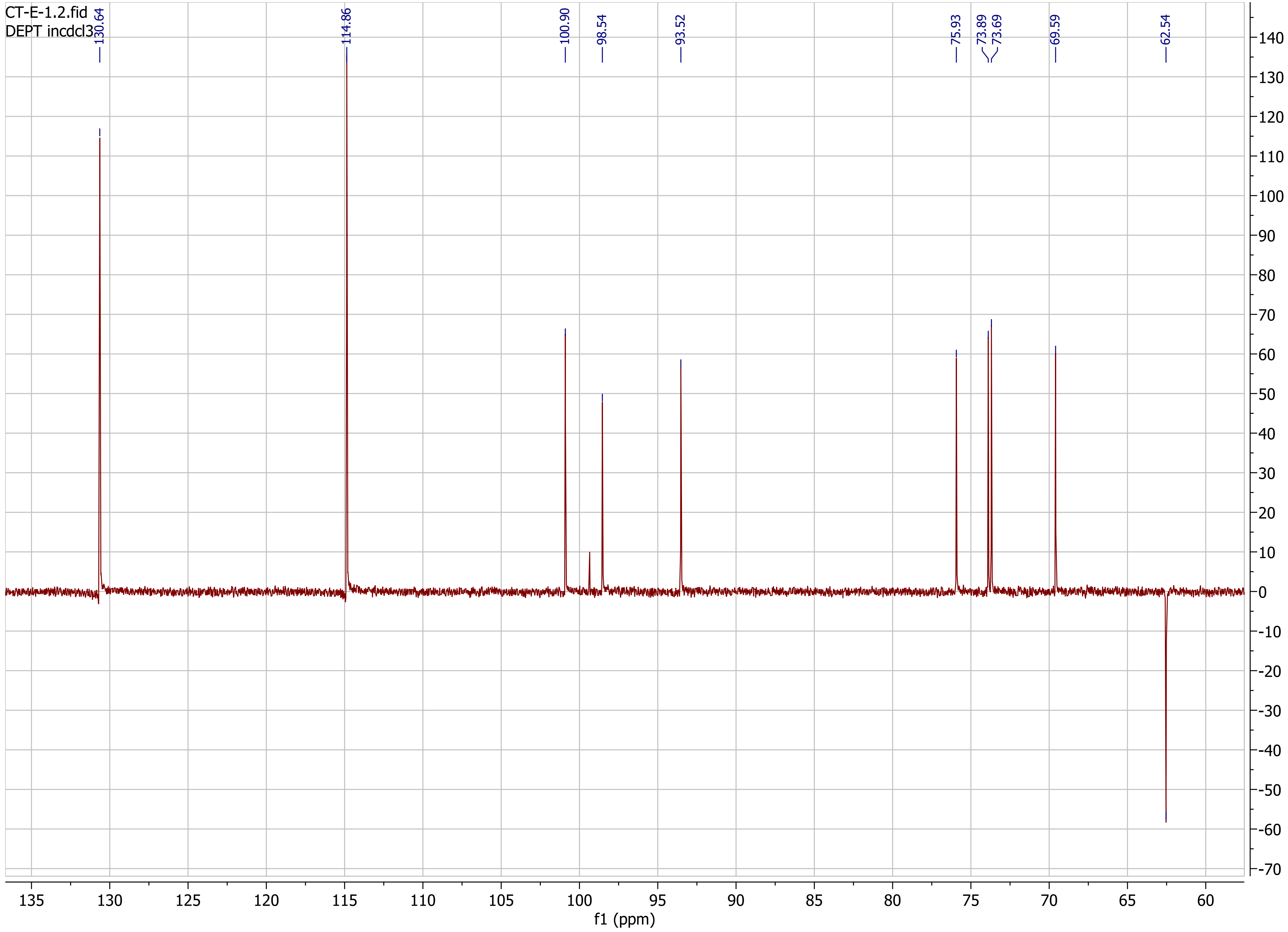

CT-E-1.2.fid
DEPT incdcl3
130.64
114.86
100.90
98.54
93.52
75.93
73.89
73.69
69.59
62.54
f1 (ppm)

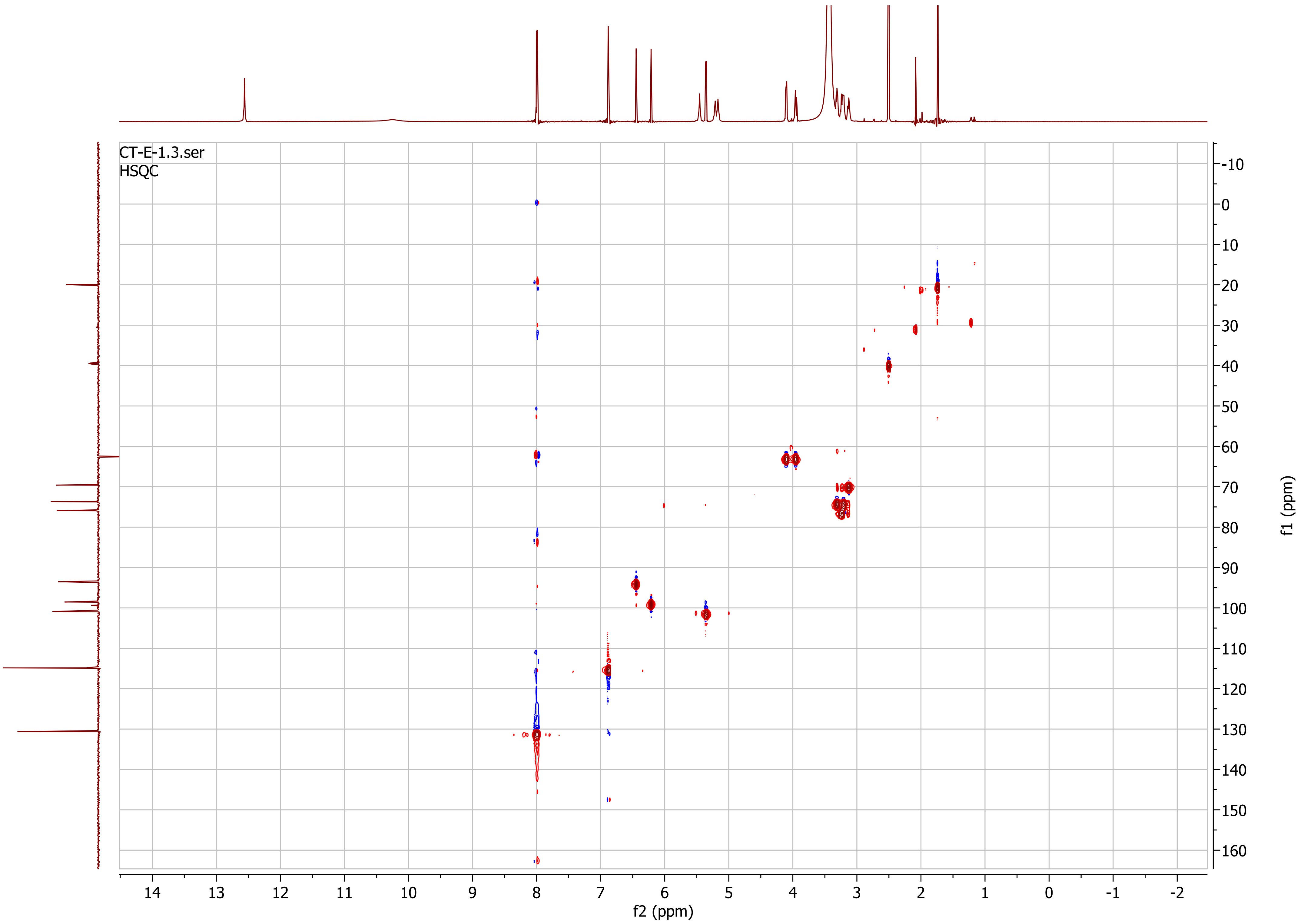
CT-E-1.3.ser
HSQC
f2 (ppm)
f1 (ppm)

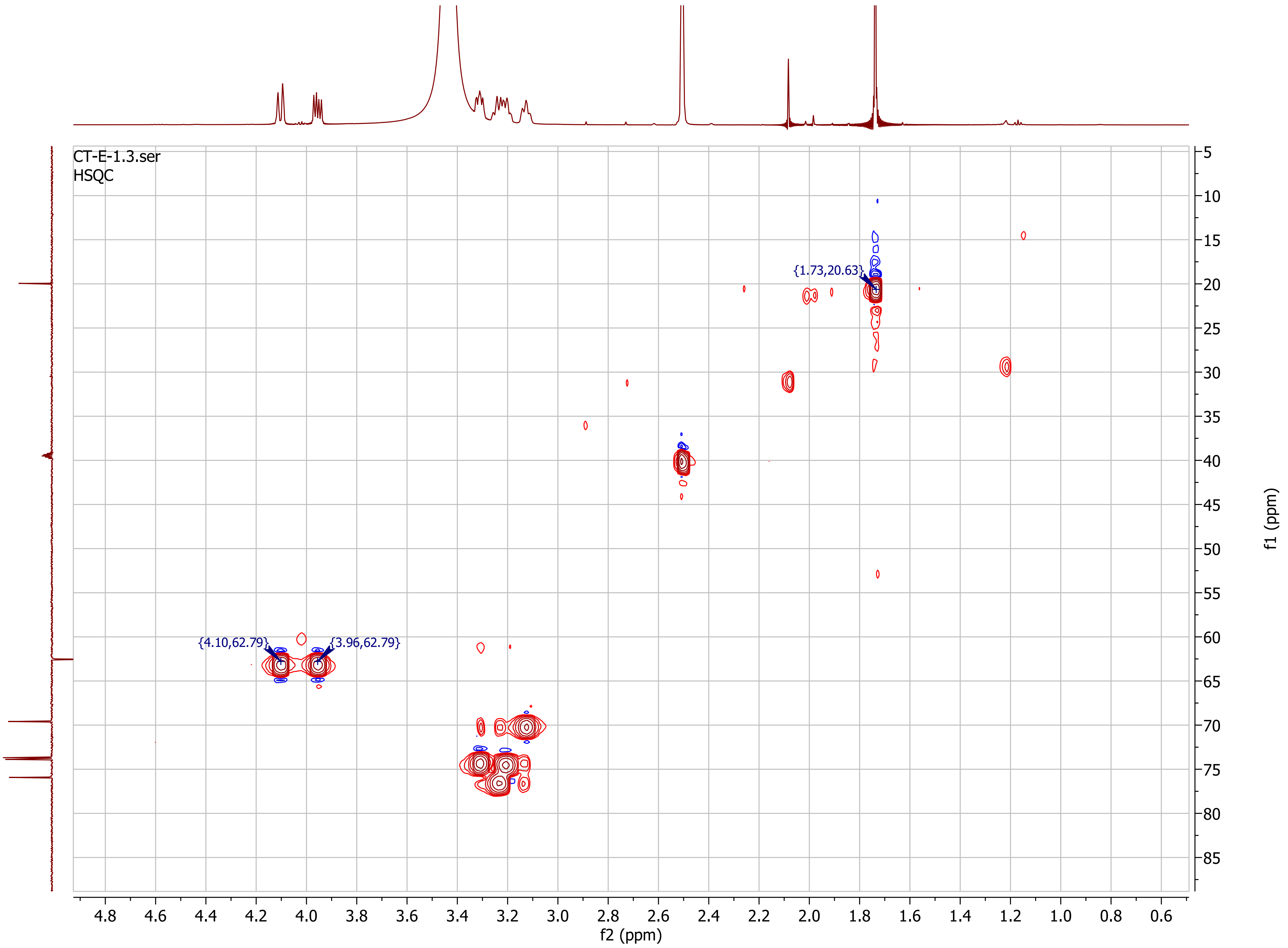

CT-E-1.3.ser
HSQC
{1.73,20.63}
{4.10,62.79}
{3.96,62.79}
f2 (ppm)
f1 (ppm)

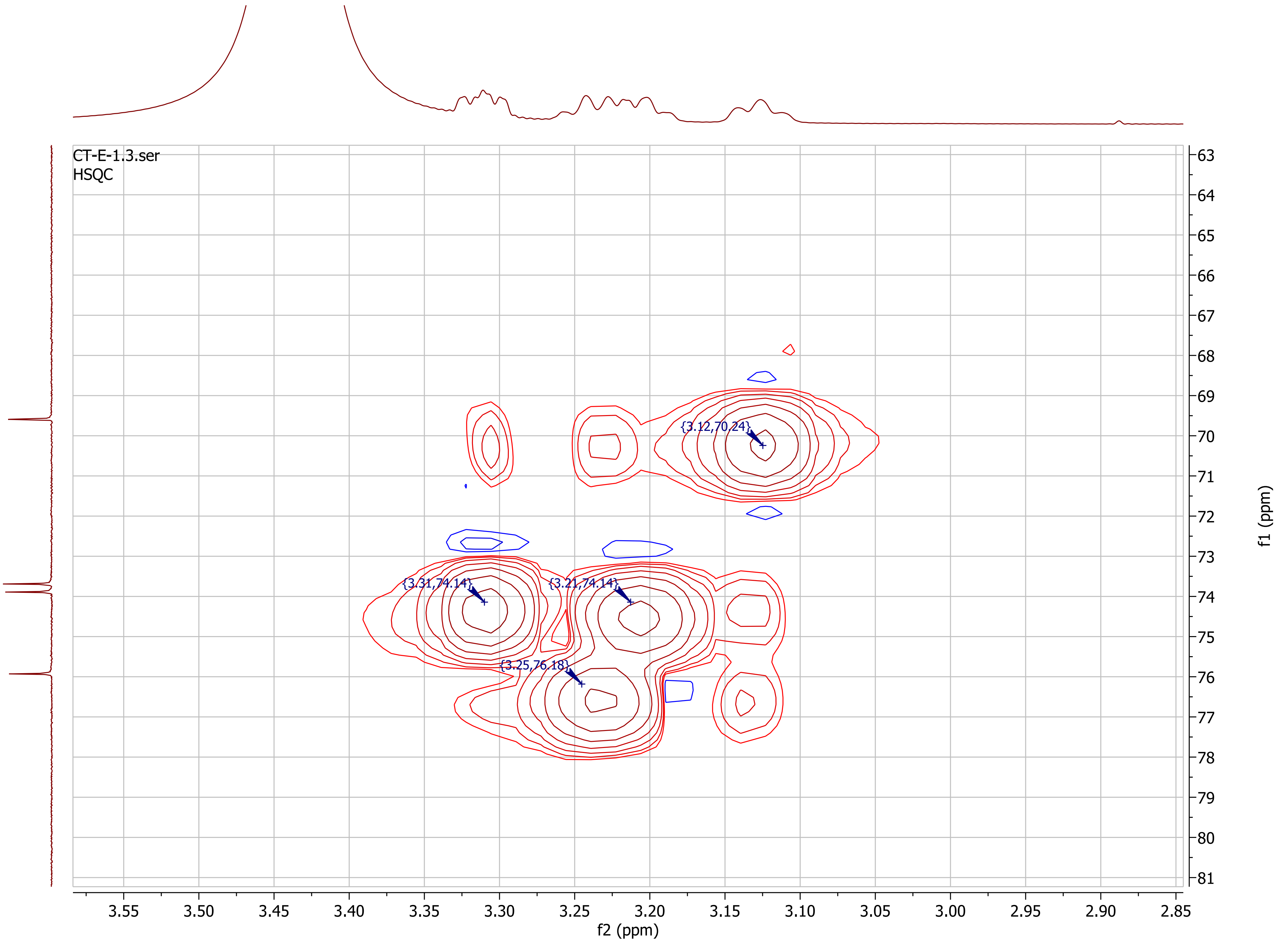
CT-E-1.3.ser
HSQC
{3.12,70.24}
{3.31,74.14}
{3.21,74.14}
{3.25,76.18}
f2 (ppm)
f1 (ppm)
3.55
3.50
3.45
3.40
3.35
3.30
3.25
3.20
3.15
3.10
3.05
3.00
2.95
2.90
2.85
63
64
65
66
67
68
69
70
71
72
73
74
75
76
77
78
79
80
81

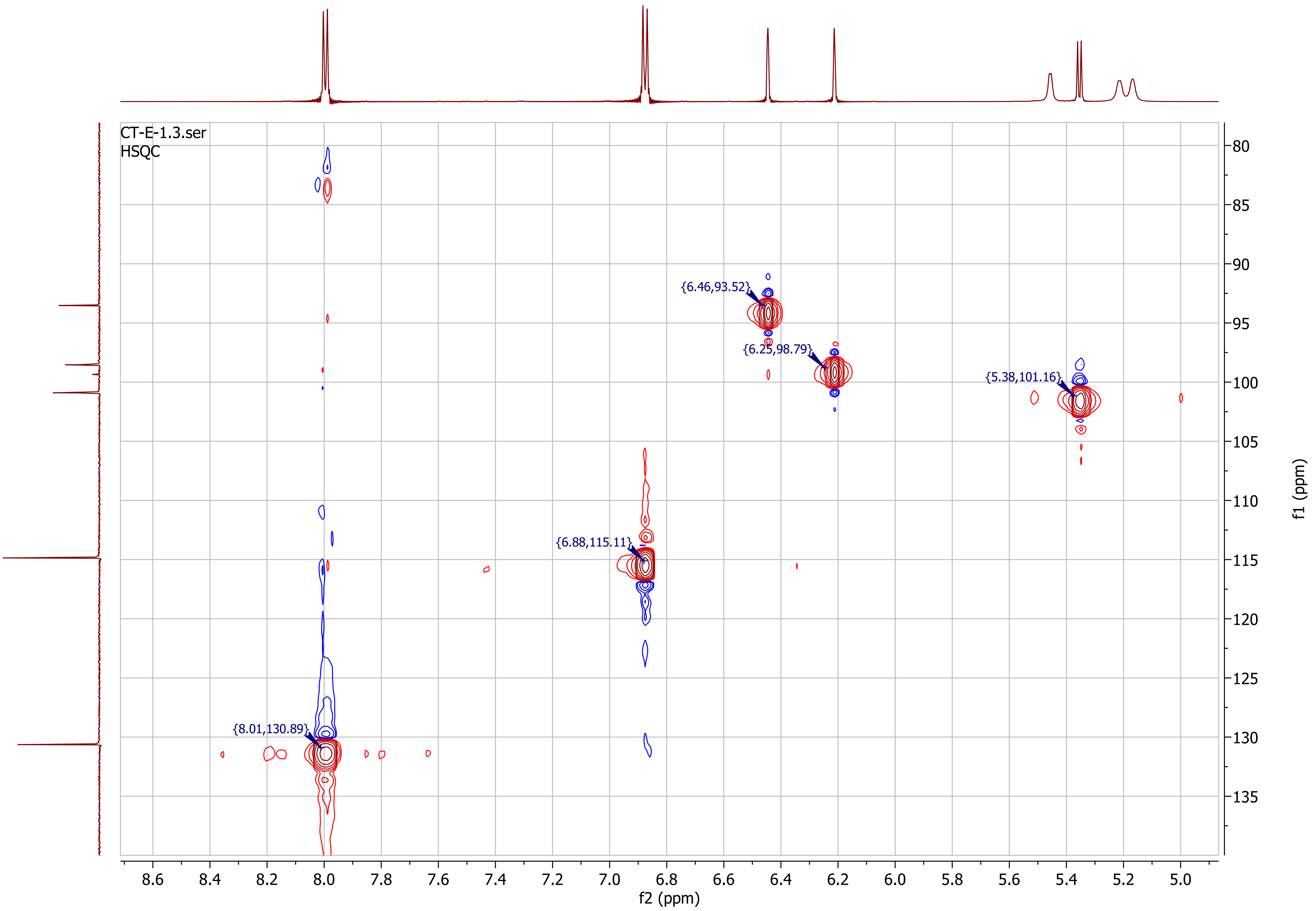
CT-E-1.3.ser
HSQC
{6.46,93.52}
{6.25,98.79}
{5.38,101.16}
{6.88,115.11}
{8.01,130.89}
f2 (ppm)
f1 (ppm)

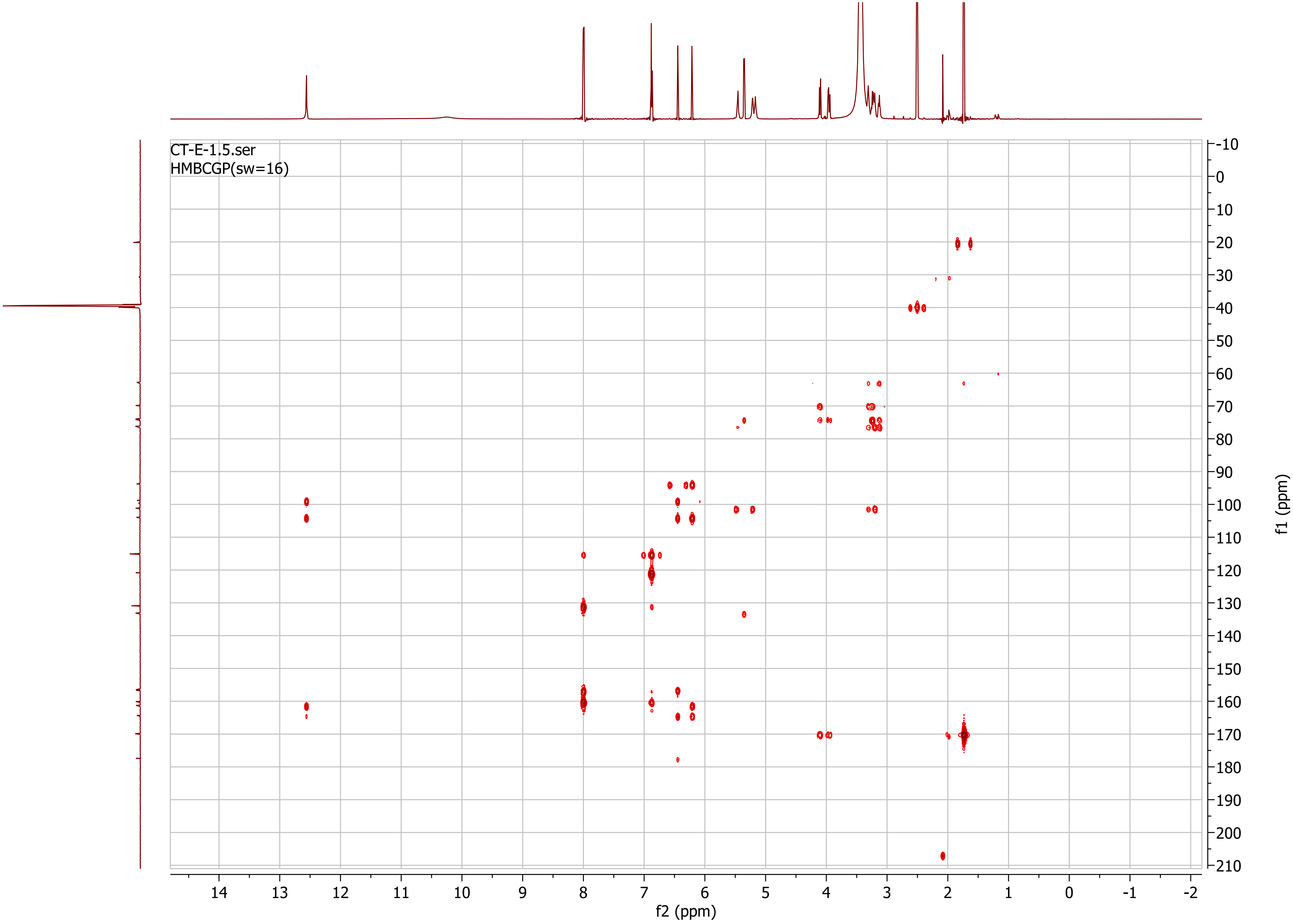
CT-E-1.5.ser
HMBCGP(sw=16)
f2 (ppm)
f1 (ppm)

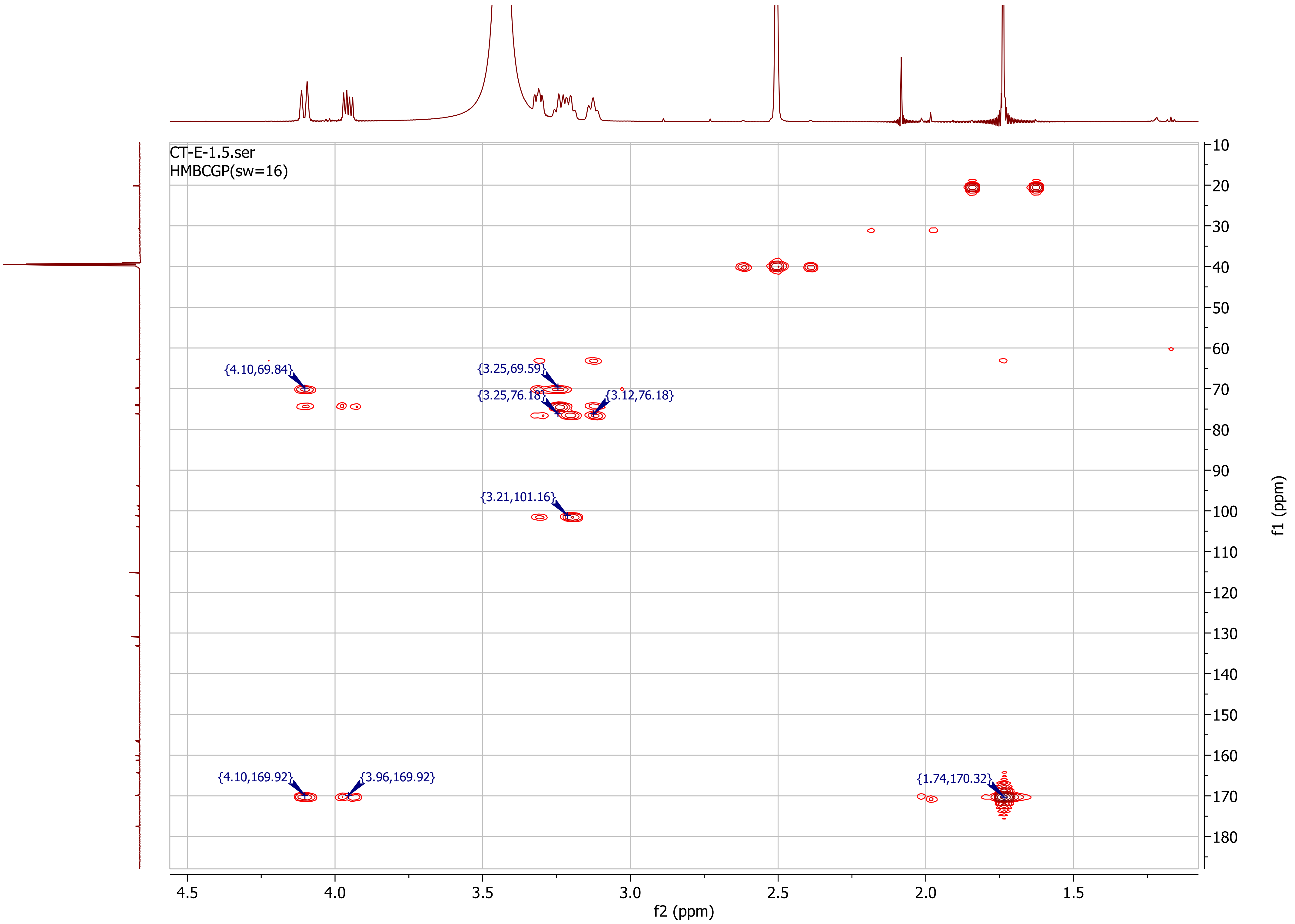
CT-E-1.5.ser
HMBCGP(sw=16)
{4.10,69.84}
{3.25,69.59}
{3.25,76.18}
{3.12,76.18}
{3.21,101.16}
{4.10,169.92}
{3.96,169.92}
{1.74,170.32}
f2 (ppm)
f1 (ppm)

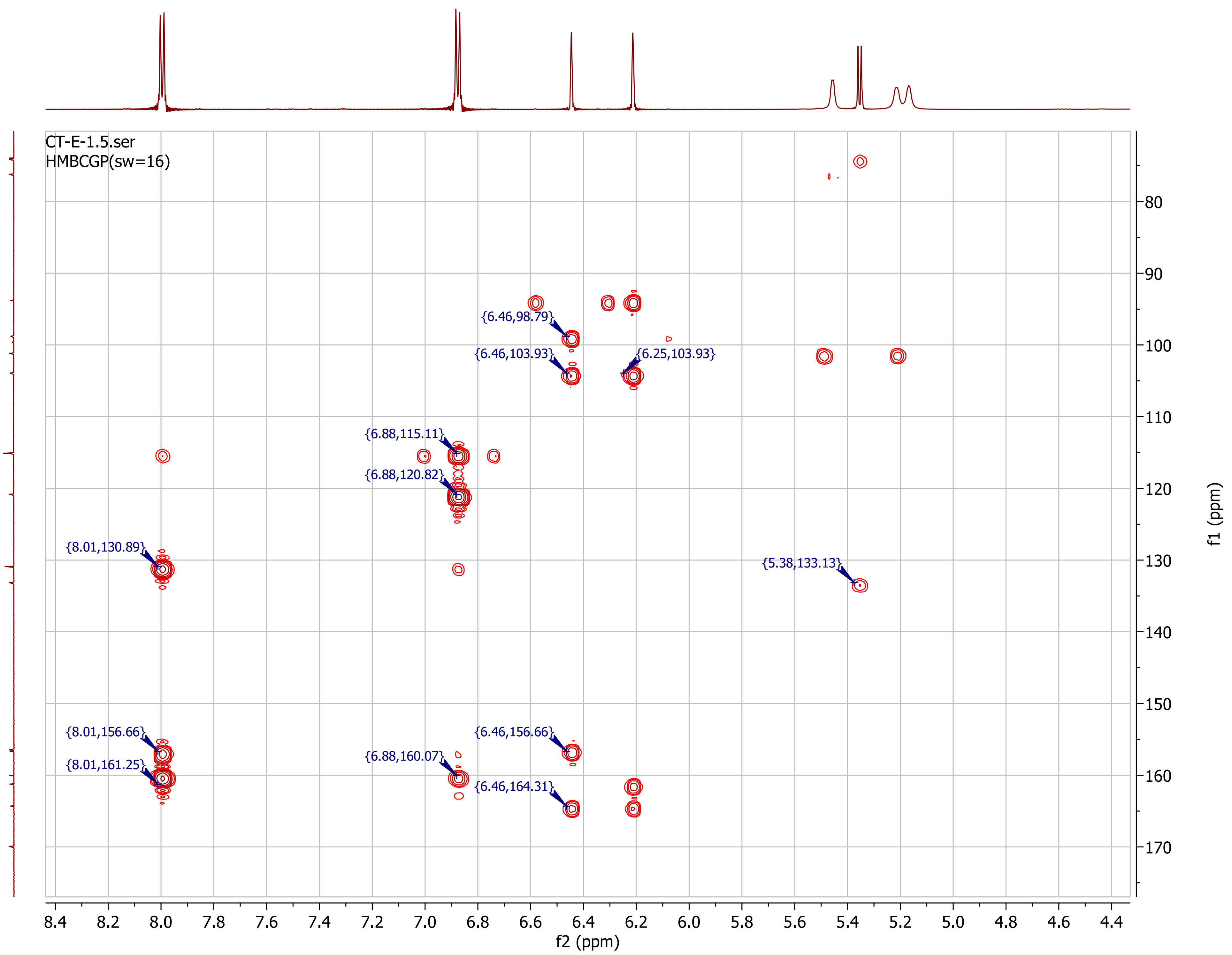
CT-E-1.5.ser
HMBCGP(sw=16)
{6.46,98.79}
{6.46,103.93}
{6.25,103.93}
{6.88,115.11}
{6.88,120.82}
{8.01,130.89}
{5.38,133.13}
{8.01,156.66}
{6.46,156.66}
{6.88,160.07}
{8.01,161.25}
{6.46,164.31}
f2 (ppm)
f1 (ppm)

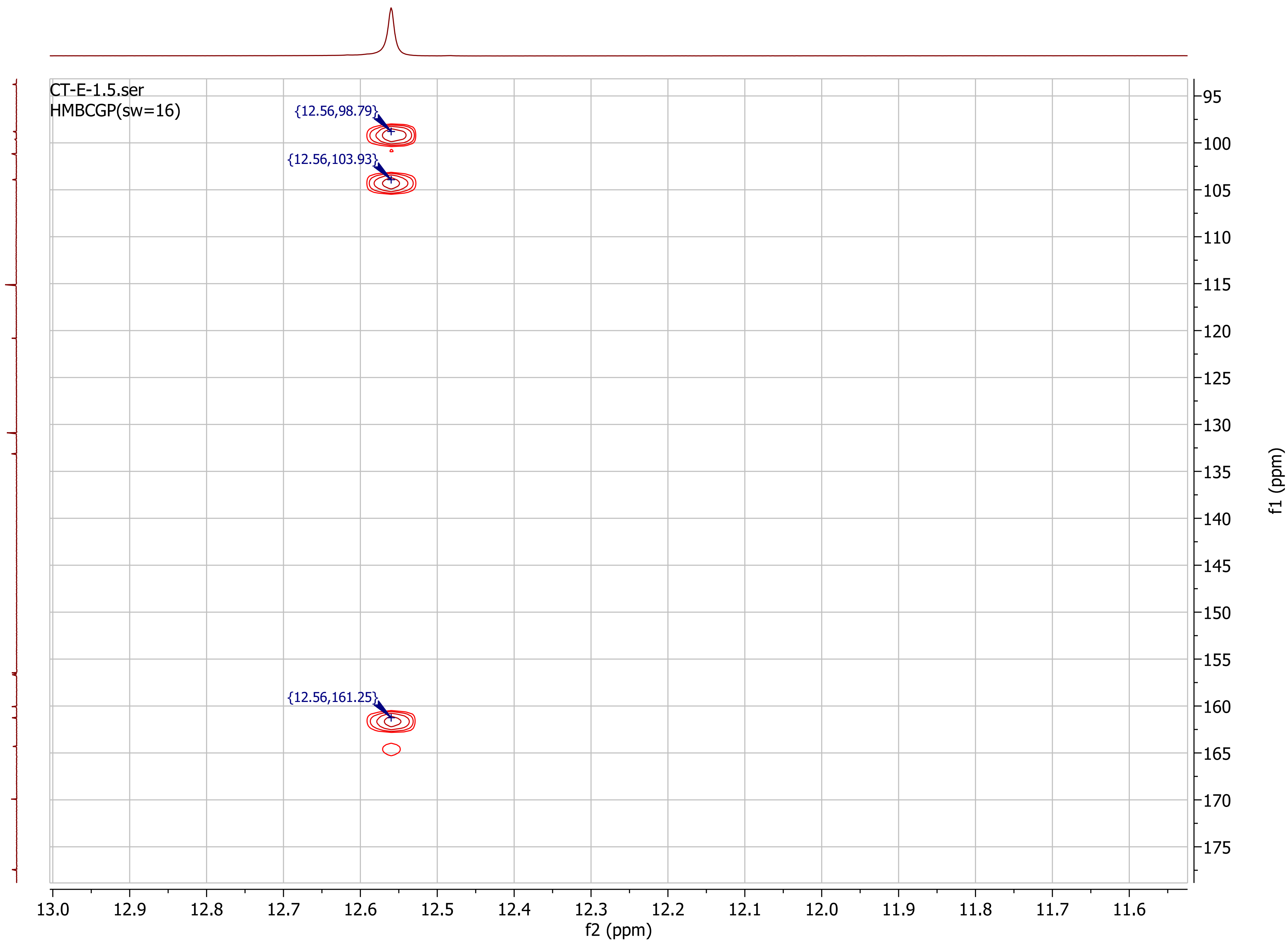
CT-E-1.5.ser
HMBCGP(sw=16)
{12.56,98.79}
{12.56,103.93}
{12.56,161.25}
f2 (ppm)
f1 (ppm)

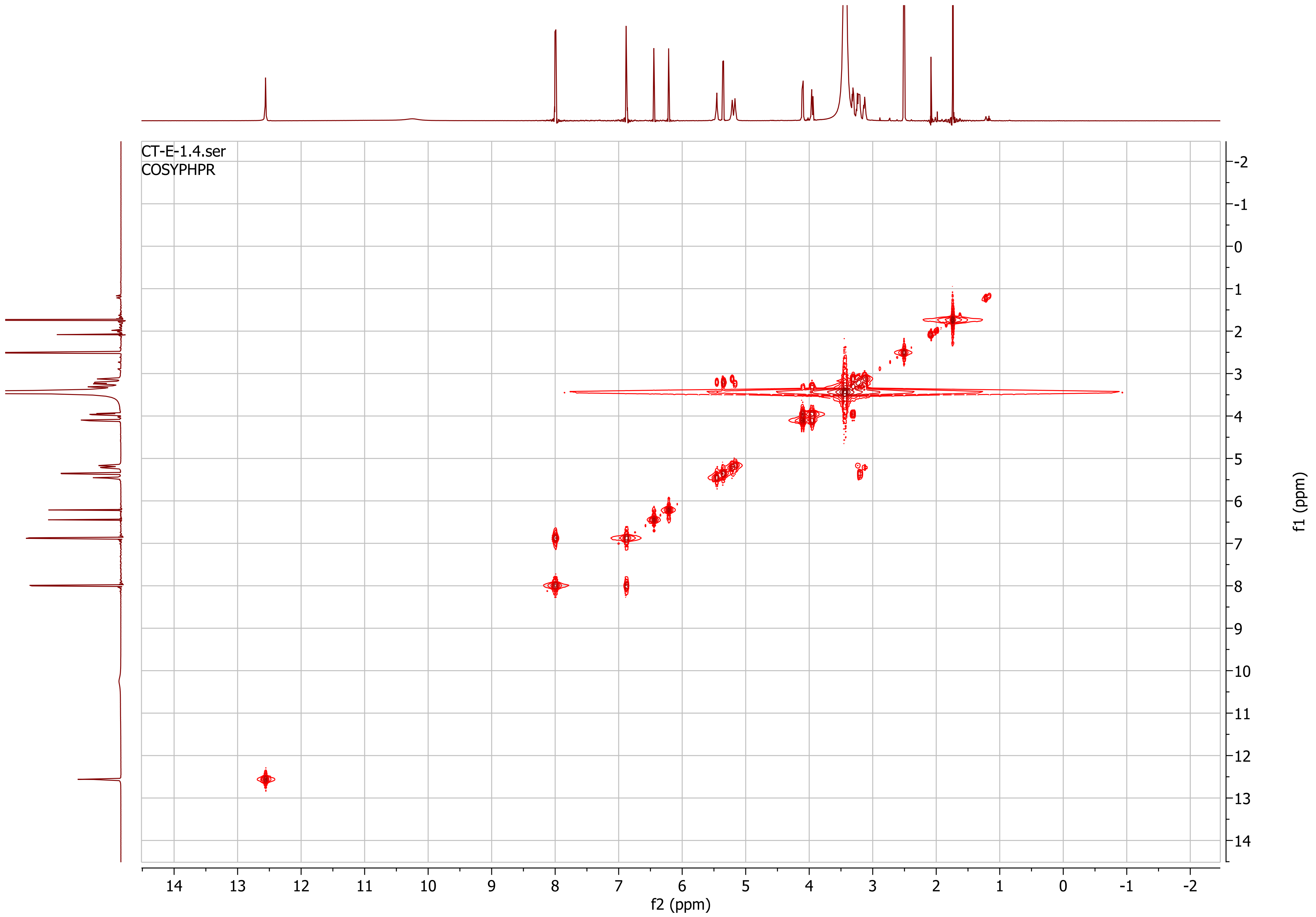

CT-E-1.4.ser
COSYPHPR
f2 (ppm)
f1 (ppm)

# Method

## 1. General experimental section

NMR spectra were carried out on a commercial instrument (Bruker Avance 300 and 600 MHz), chemical shifts (δ) are presented in parts per million (ppm) and re-calculated with respect to tetramethylsilane (TMS) ($^1H$) or carbon signals of deuterium solvents ($^{13}C$). Spin-spin coupling constants (J) are given in hertz (Hz). Mass spectra were recorded on an HP5989A instrument (CI and EI, ionization energy 70 eV) with Apollo 300 data, and on a Kratos MS50TC instrument for accurate calculations (reaching by electric shock (ESI), common solvent mixture: $CH_2Cl_2$-MeOH + $NH_4OAc$) with MASSLYNX system data. UV spectra were obtained on a Perkin-Elmer Lambda 20 Spectrometer instrument. Melting points were determined on Reichert Thermovar. For column chromatography, silica gel 0.06-0.2 mm (Acros) was used as the stationary phase. Silica gel 32-63 mesh was used for flash column chromatography.

## 2. Isolation

To study the component composition of *Calligonum tetrapterum*, we used the aboveground part of the raw material (green shots) collected in the vicinity of Sarkand, Almaty region phase fruiting. The specimen is identified by Ishmuratova M.Yu., herbarium sample is located in herbarium fund of Zhezkazgan botanical garden (N2007.09.12.03.01).

Finely raw material *C. tetrapterum* (1 kg) was extracted three times with 70% ethanol by keeping in it at room temperature for 3 days. The extracts were combined, the solvent was evaporated on a rotary evaporator, and the resulting extract was extracted with petroleum ether and ether.

From 10 g of ether extract of 70% alcoholic extract of *C. tetrapterum* raw material by chromatographic separation on silica gel. Fractions of 350 ml were taken and a total of 62 fractions were obtained. By elution with a heptane-ethyl acetate (7:3) system, a compound was isolated, which, according to mass spectral data, so pl. and was identified by $^1H$ NMR as beta-sitosterol **3**.

Compound **2** was isolated by further separation on silica gel by elution with a solvent mixture of heptane-ethyl acetate (1:7). Its structure was determined by $^{1}H$ and $^{13}C$ NMR spectroscopy. Compound **2**was obtained in the form of yellow crystals with a melting point of 277-278 °C. Its molecular formula $C_{15}H_{10}O_{6}$was confirmed by mass spectroscopy, which indicated the peak ion [M+H] _c m/z 287.

According to the data obtained, compound **2** was identified as kaempferol, which was previously isolated from some species of the *Artemisia* family and other plant species. The structure of this compound was also unambiguously established on the basis of mass spectra, $^{1}H$ and $^{13}C$ NMR data, UV spectra and their comparison with literature data.

Further elution with a heptane-ethyl acetate (1:100) solvent system gave a white amorphous solid with m.p. 271-275 °C. In accordance with the spectral data, it was proposed the structure of the kaempferol glycoside. Spectral data **1** are presented in Table 1.

**kaempferol 3-*O*-(6''-*O*-acetyl)-glucoside (1);**

Yellow crystals with so pl. 271-275 °C; UV max (ACN-$H_2O$) 263, 295 (shoulder) and 350 nm; $^{1}H$ and $^{13}C$ NMR (DMSO-$d_6$) (see Table 1).

**Kaempferol (2):**

Isolated by elution with a heptane-ethyl acetate system (1:7); Yellow crystals, m.p. 277-278 ºC (lit. 272-275 ºC); Mass spectra: $[M+H]^{-}$ m/z 287. UV max (ACN-$H_2O$) 270, 294 (shoulder) and 360 nm;

**β-Sitosterol (3):**

Isolated by elution with a heptane-ethyl acetate system (7:3). White crystals, m.p. 140-145 ºC; Mass spectra: $[M+H]^{+}$ c m/z 415

**Molecular Similarity**

Molecular Similarity of the tested compound against the eight co-crystallized ligands of SARS-Cov-2 was carried out calculated using Discovery studio 4.0. At first, the CHARMM force field was applied then the compound was prepared using prepare ligand protocol. Then, the tested compound was used as a reference compound while the co-crystallized ligands were used as a test set. The protocol was adjusted to give one output. The default molecular properties were applied. The molecular properties include the number of rotatable bonds, number of rings, number of aromatic rings, number of hydrogen bond donors (HBA), number of hydrogen bond acceptors (HBD), partition coefficient (ALog p), molecular weight (M. Wt), and molecular fractional polar surface area (MFPSA).

**3. DFT**

The DFT parameters (total energy, binding energy, HOMO, LUMO, gap energy, dipole moment, and electrostatic potential) were calculated using Discovery studio software. the tested compounds were prepared using prepare ligand protocol. Then, the prepared compounds were subjected to DFT calculation protocol using the default option

**4. Docking studies**

**Protein Preparation:** The crystal structure of the employed protein, SARS-CoV-2 RNA-dependent RNA polymerase (PDB ID: 7BV2), was obtained from Protein Data Bank (https://www.rcsb.org). At first, the crystal structure of the SARS-CoV-2 RNA-dependent RNA polymerase (PDB ID: 7BV2) complexed with the remdesivir ligand was prepared by removing crystallographic water molecules. Only one chain was retained besides the co-crystallized ligand (remdesivir). The selected protein chain was protonated using the following setting. The used electrostatic functional form was GB/VI with a distance cut-off of 15 Å. The used value of the dielectric constant was 2 with an 80 dielectric constant of the used solvent. The used Van der Waals functional form was 800R3 with a distance cut-off of 10 Å. Then, the energy of the protein chain was minimized using Hamiltonian AM1 implanted in Molecular Operating Environment (MOE 2019 and MMFF94x (Merck molecular force field) for structural optimization. Next, the

active site of the target protein was defined for ligand docking and redocking (in case of validation of docking protocol). The active site of the protein was identified as the residues that fall within the 5 Å distance from the perimeter of the co-crystallized ligand.

**Ligand Preparation:** 2D structures of kaempferol 3-*O*-(6''-*O*-acetyl)-glucoside and the standard ligand (remdesivir) were drawn using ChemBioDraw Ultra 14.0 and saved in MDL-SD file format. The 3D structures of the ligands were protonated, and the structures were optimized by energy minimization using MM2 force-field and 10000 iteration steps of 2 fs. The conformationally optimized ligands were used for docking studies.

**Docking Setup and Validation of Docking Protocol:** The protein-ligand docking studies were carried out using MOE version 2019. Validation of the docking protocol was carried out by redocking the co-crystallized reference ligand (remdesivir) against the isolated pocket of SARSCoV-2 RNA-dependent RNA polymerase (PDB ID: 7BV2). The docking protocol was validated by comparing the heavy atoms RMSD value of the re-docked ligand pose with the corresponding co-crystallized reference ligand structure.

The docking setup for the tested compounds was established according to the protocol followed in the validation step. For each docking run, 30 docked solutions were generated using ASE for scoring function and rigid receptor for refinement. The pose with ideal binding mode was selected for further investigations. The docking results were visualized using Discovery Studio (DS) 4.0. Analysis of the docking results was carried out by comparing the interactions and docking score obtained for the docked ligands with that of the re-docked reference molecul).

## 5. ADMET

ADMET descriptors (absorption, distribution, metabolism, excretion and toxicity) of the compounds were determined using Discovery studio 4.0. At first, the CHARMM force field was applied then the tested compounds were prepared and minimized according to the preparation of small molecule protocol. Then ADMET descriptors protocol was applied to carry out these studies.

### 6. Toxicity studies

The toxicity parameters of the tested compounds were calculated using Discovery studio 4.0. Indinavir was used as a reference drug. At first, the CHARMM force field was applied then the compounds were prepared and minimized according to the preparation of small molecule protocol. Then different parameters were calculated from the toxicity prediction (extensible) protocol.

### 7. Molecular dynamics simulations

The system was prepared using the web-based CHARMM-GUI interface with the CHARMM36 force field. All the simulations were done using the NAMD 2.13 package. The TIP3P explicit solvation model was used, and the periodic boundary conditions were set with a dimension of the dimensions Å in x, y, and z, respectively. The parameters for the top docking results were generated using the CHARMM general force field Afterward, the system was neutralized using ---- ($Cl^-$/$Na^+$) ions. The MD protocols involved minimization, equilibration, and production. a 2 *fs* time step of integration was chosen for all MD simulations, the equilibration was carried in the canonical (*NVT*) ensemble, while the isothermal–isobaric (*NPT*) ensemble was for the production. Through the 100 *ns* of MD production, the pressure was set at 1 atm using the Nose´–Hoover Langevin piston barostat with a Langevin piston decay of 0.05 *ps* and a period of 0.1 *ps*. The temperature was set at 298.15 K using the Langevin thermostat. A distance cutoff of 12.0 Å was applied to short-range nonbonded interactions with a pair list distance of 16 Å, and Lennard Jones interactions were smoothly truncated at 8.0 Å. Long-range electrostatic interactions were treated using the particle-mesh Ewald (PME) method, where a grid spacing of 1.0 Å was used for all simulation cells. All covalent bonds involving hydrogen atoms were constrained using the SHAKE algorithm. For consistency, we have applied the same protocol for all MD simulations.

# Toxicity Report

# Remdesivir

# TOPKAT_Developmental_Toxicity_Potential

AND Enantiomer

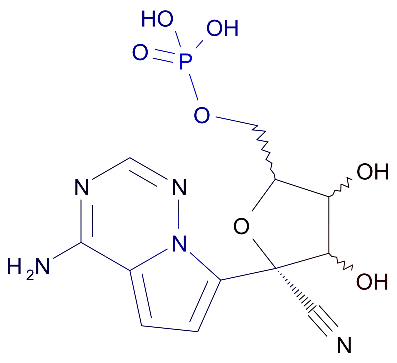

$C_{12}H_{14}N_5O_7P$
Molecular Weight: 371.24262
ALogP: -1.502
Rotatable Bonds: 4
Acceptors: 11
Donors: 5

## Model Prediction

Prediction: Non-Toxic
Probability: 0.373
Enrichment: 0.709
Bayesian Score: -5.42
Mahalanobis Distance: 9.05
Mahalanobis Distance p-value: 0.163

Prediction: Positive if the Bayesian score is above the estimated best cutoff value from minimizing the false positive and false negative rate.
Probability: The esimated probability that the sample is in the positive category. This assumes that the Bayesian score follows a normal distribution and is different from the prediction using a cutoff.
Enrichment: An estimate of enrichment, that is, the increased likelihood (versus random) of this sample being in the category.
Bayesian Score: The standard Laplacian-modified Bayesian score.
Mahalanobis Distance: The Mahalanobis distance (MD) is the distance to the center of the training data. The larger the MD, the less trustworthy the prediction.
Mahalanobis Distance p-value: The p-value gives the fraction of training data with an MD greater than or equal to the one for the given sample, assuming normally distributed data. The smaller the p-value, the less trustworthy the prediciton. For highly non-normal X properties (e.g., fingerprints), the MD p-value is wildly inaccurate.

## Structural Similar Compounds

| Name | Sinigrin (Free Acid Form) | Azthreonam | Vidarabine |
|---|---|---|---|
| Structure | 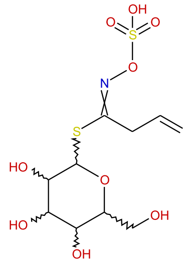 | 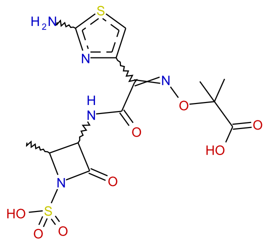 | 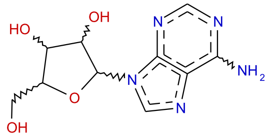 |
| Actual Endpoint | Toxic | Non-Toxic | Non-Toxic |
| Predicted Endpoint | Toxic | Non-Toxic | Non-Toxic |
| Distance | 0.632 | 0.707 | 0.714 |
| Reference | Food Cosmet Toxicol 18(2):159-72; 1980 | Chemotherapy 33:203-218; 1985 | Teratology 15(3):231-41; 1977 |

## Model Applicability

Unknown features are fingerprint features in the query molecule, but not found or appearing too infrequently in the training set.

1. All properties and OPS components are within expected ranges.

## Feature Contribution

| Top features for positive contribution | | | | |
|---|---|---|---|---|
| **Fingerprint** | **Bit/Smiles** | **Feature Structure** | **Score** | **Toxic in training set** |
| SCFP_6 | -1486266146 | AND Enantiomer 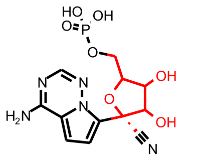 [*]CC1OC([*])([*])C(O )C1O | 0.431 | 7 out of 8 |

| | | | | |
|---|---|---|---|---|
| SCFP_6 | -1715619483 | 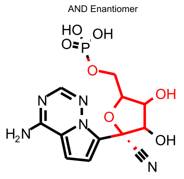 [*]OCC1OC([*])([*])C([*])C1O | 0.298 | 6 out of 8 |
| SCFP_6 | -1181430618 | 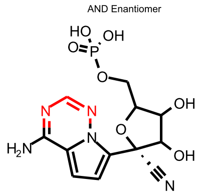 [*]:n:[cH]:n:[*] | 0.298 | 6 out of 8 |

## Top Features for negative contribution

| Fingerprint | Bit/Smiles | Feature Structure | Score | Toxic in training set |
|---|---|---|---|---|
| SCFP_6 | -1375522316 | 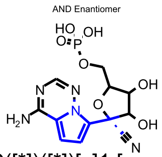 [*]C([*])([*])[c]1:[cH]:[cH]:[c](:[*]):n:1:[*] | -0.945 | 0 out of 3 |
| SCFP_6 | 2108966103 | 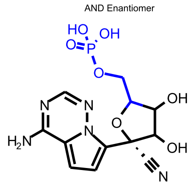 [*]C([*])COP(=O)(O)O | -0.945 | 0 out of 3 |
| SCFP_6 | 269938867 | 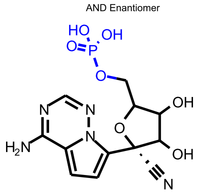 [*]OP(=O)(O)O | -0.729 | 1 out of 6 |

# Flavonoid

# TOPKAT_Developmental_Toxicity_Potential

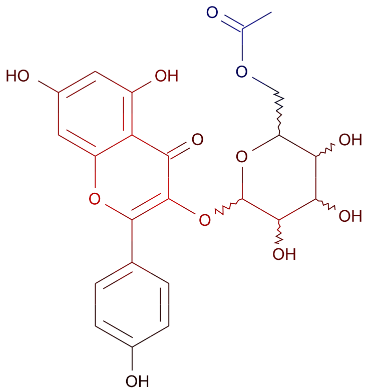


$C_{23}H_{22}O_{12}$
Molecular Weight: 490.41357
ALogP: 0.322
Rotatable Bonds: 6
Acceptors: 12
Donors: 6

## Model Prediction

Prediction: Toxic
Probability: 0.808
Enrichment: 1.54
Bayesian Score: 6.94
Mahalanobis Distance: 11.4
Mahalanobis Distance p-value: 0.000317

Prediction: Positive if the Bayesian score is above the estimated best cutoff value from minimizing the false positive and false negative rate.
Probability: The esimated probability that the sample is in the positive category. This assumes that the Bayesian score follows a normal distribution and is different from the prediction using a cutoff.
Enrichment: An estimate of enrichment, that is, the increased likelihood (versus random) of this sample being in the category.
Bayesian Score: The standard Laplacian-modified Bayesian score.
Mahalanobis Distance: The Mahalanobis distance (MD) is the distance to the center of the training data. The larger the MD, the less trustworthy the prediction.
Mahalanobis Distance p-value: The p-value gives the fraction of training data with an MD greater than or equal to the one for the given sample, assuming normally distributed data. The smaller the p-value, the less trustworthy the prediciton. For highly non-normal X properties (e.g., fingerprints), the MD p-value is wildly inaccurate.

## Structural Similar Compounds

| Name | Cefpiramide Sodium (Free acid form) | Latomoxef | Sinigrin (Free Acid Form) |
|---|---|---|---|
| Structure | 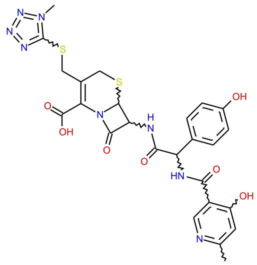 | 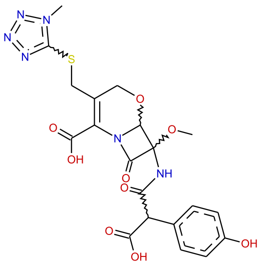 | 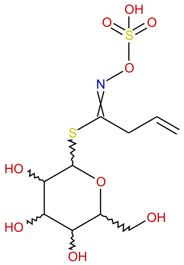 |
| Actual Endpoint | Non-Toxic | Toxic | Toxic |
| Predicted Endpoint | Non-Toxic | Non-Toxic | Toxic |
| Distance | 0.733 | 0.783 | 0.804 |
| Reference | Kiso to Rinsho 17:1000-1004; 1983 | Chemotherapy 28:1119-1141; 1980 | Food Cosmet Toxicol 18(2):159-72; 1980 |

## Model Applicability

Unknown features are fingerprint features in the query molecule, but not found or appearing too infrequently in the training set.

1. OPS PC19 out of range. Value: -2.9432. Training min, max, SD, explained variance: -2.7817, 3.2747, 1.037, 0.0157.

## Feature Contribution

| Top features for positive contribution | | | | |
|---|---|---|---|---|
| Fingerprint | Bit/Smiles | Feature Structure | Score | Toxic in training set |
| SCFP_6 | -2031220028 | 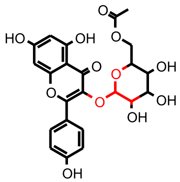 [*]OC(O[*])C([*])[*] | 0.538 | 7 out of 7 |

| | | | | |
|---|---|---|---|---|
| SCFP_6 | -37268149 | 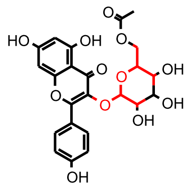 [*]CC1OC(O[*])C([*])[ *]C1[*] | 0.523 | 6 out of 6 |
| SCFP_6 | 616636418 | 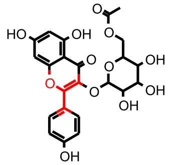 [*]OC(=C([*])[*])[c]( :[*]):[*] | 0.478 | 4 out of 4 |

**Top Features for negative contribution**

| **Fingerprint** | **Bit/Smiles** | **Feature Structure** | **Score** | **Toxic in training set** |
|---|---|---|---|---|
| SCFP_6 | 733985735 | 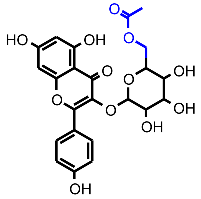 [*]COC(=O)C | -0.945 | 0 out of 3 |
| SCFP_6 | 1112262477 | 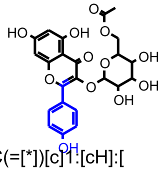 [*]C(=[*])[c]1:[cH]:[ cH]:[c](O):[cH]:[cH] :1 | -0.422 | 0 out of 1 |
| SCFP_6 | -183093204 | 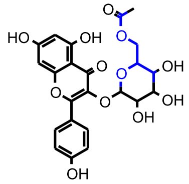 [*]OC(COC(=[*])[*])C( [*])[*] | -0.422 | 0 out of 1 |

# Remdesivir

# TOPKAT_Mouse_Female_FDA_None_vs_Carcinogen

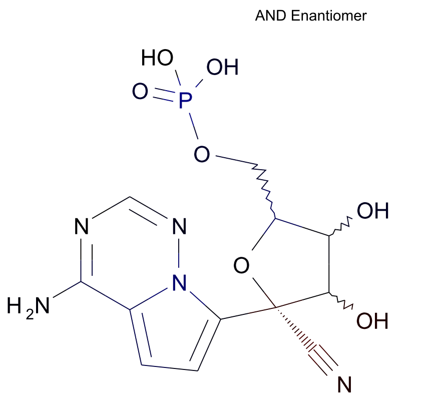


$C_{12}H_{14}N_5O_7P$

Molecular Weight: 371.24262

ALogP: -1.502

Rotatable Bonds: 4

Acceptors: 11

Donors: 5

## Model Prediction

Prediction: Non-Carcinogen

Probability: 0.206

Enrichment: 0.642

Bayesian Score: -7.17

Mahalanobis Distance: 12.9

Mahalanobis Distance p-value: 0.00074

Prediction: Positive if the Bayesian score is above the estimated best cutoff value from minimizing the false positive and false negative rate.
Probability: The esimated probability that the sample is in the positive category. This assumes that the Bayesian score follows a normal distribution and is different from the prediction using a cutoff.
Enrichment: An estimate of enrichment, that is, the increased likelihood (versus random) of this sample being in the category.
Bayesian Score: The standard Laplacian-modified Bayesian score.
Mahalanobis Distance: The Mahalanobis distance (MD) is the distance to the center of the training data. The larger the MD, the less trustworthy the prediction.
Mahalanobis Distance p-value: The p-value gives the fraction of training data with an MD greater than or equal to the one for the given sample, assuming normally distributed data. The smaller the p-value, the less trustworthy the prediciton. For highly non-normal X properties (e.g., fingerprints), the MD p-value is wildly inaccurate.

## Structural Similar Compounds

| Name | Famotidine | Tetracycline | Oxytetracycline |
|---|---|---|---|
| Structure | 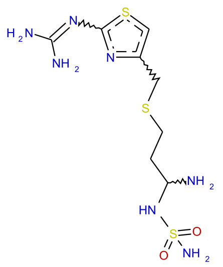 | 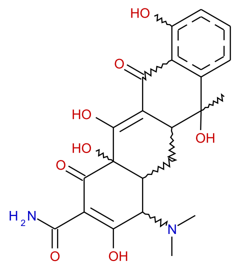 | 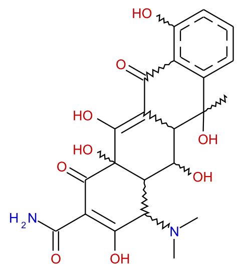 |
| Actual Endpoint | Non-Carcinogen | Non-Carcinogen | Non-Carcinogen |
| Predicted Endpoint | Non-Carcinogen | Non-Carcinogen | Non-Carcinogen |
| Distance | 0.846 | 0.848 | 0.870 |
| Reference | US FDA (Centre for Drug Eval.& Res./Off. Testing & Res.) Sept. 1997 | US FDA (Centre for Drug Eval.& Res./Off. Testing & Res.) Sept. 1997 | US FDA (Centre for Drug Eval.& Res./Off. Testing & Res.) Sept. 1997 |

## Model Applicability

Unknown features are fingerprint features in the query molecule, but not found or appearing too infrequently in the training set.

1. All properties and OPS components are within expected ranges.
2. Unknown ECFP_2 feature: 1126642748: [*]OP(=O)(O)O
3. Unknown ECFP_2 feature: -1250439909: [*]COP(=[*])([*])[*]
4. Unknown ECFP_2 feature: 1258791451: [*]C1[*][*]O[C@]1(C#[*])[c](:[*]):[*]
5. Unknown ECFP_2 feature: -1507082173: [*][c]1:[*]:[*]:[c](:[*]):n:1:n:[*]
6. Unknown ECFP_2 feature: -66263742: [*]C([*])([*])[c]1:[cH]:[*]:[*]:n:1:[*]

## Feature Contribution

**Top features for positive contribution**

| Fingerprint | Bit/Smiles | Feature Structure | Score | Carcinogen in training set |
|---|---|---|---|---|

| | | | | |
|---|---|---|---|---|
| ECFP_6 | -1114776580 | 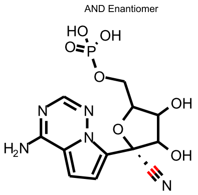 [*]C#[*] | 0.755 | 11 out of 15 |
| ECFP_6 | -521596699 | 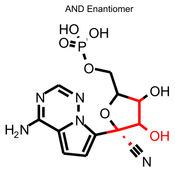 [*]C1[*][*]C([*])([*] )C1O | 0.451 | 3 out of 5 |
| ECFP_6 | -264833661 | 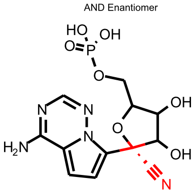 [*]C([*])([*])C#N | 0.424 | 1 out of 1 |
| **Top Features for negative contribution** | | | | |
| **Fingerprint** | **Bit/Smiles** | **Feature Structure** | **Score** | **Carcinogen in training set** |
| ECFP_6 | 1334415134 | 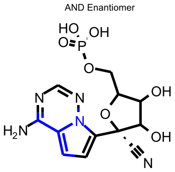 [*][c](:[*]):[c]1:[cH ]:[*]:[*]:n:1:[*] | -0.935 | 0 out of 5 |
| ECFP_6 | 2100964382 | 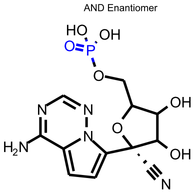 [*]P(=O)([*])[*] | -0.935 | 0 out of 5 |
| | | | | |

| | | | | |
|---|---|---|---|---|
| ECFP_6 | -826638028 | 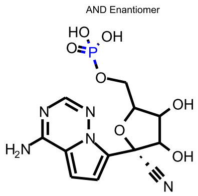 [*]P(=[*])([*])[*] | -0.935 | 0 out of 5 |

# Flavonoid

# TOPKAT_Mouse_Female_FDA_None_vs_Carcinogen

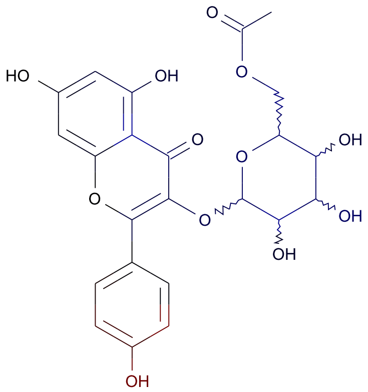

$C_{23}H_{22}O_{12}$
Molecular Weight: 490.41357
ALogP: 0.322
Rotatable Bonds: 6
Acceptors: 12
Donors: 6

## Structural Similar Compounds

| Name | Daunorubicin | Methotrexate | Minocycline |
|---|---|---|---|
| Structure | 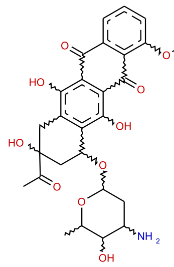 | 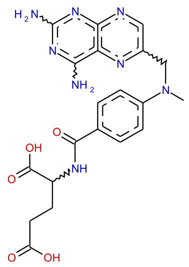 | 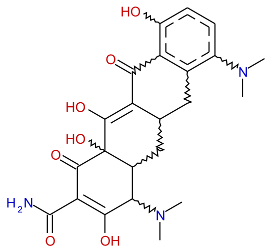 |
| Actual Endpoint | Non-Carcinogen | Non-Carcinogen | Non-Carcinogen |
| Predicted Endpoint | Non-Carcinogen | Non-Carcinogen | Non-Carcinogen |
| Distance | 0.668 | 0.712 | 0.809 |
| Reference | US FDA (Centre for Drug Eval.& Res./Off. Testing & Res.) Sept. 1997 | US FDA (Centre for Drug Eval.& Res./Off. Testing & Res.) Sept. 1997 | US FDA (Centre for Drug Eval.& Res./Off. Testing & Res.) Sept. 1997 |

## Model Prediction

Prediction: Non-Carcinogen
Probability: 0.211
Enrichment: 0.658
Bayesian Score: -8.44
Mahalanobis Distance: 15.1
Mahalanobis Distance p-value: 1.61e-007

Prediction: Positive if the Bayesian score is above the estimated best cutoff value from minimizing the false positive and false negative rate.
Probability: The esimated probability that the sample is in the positive category. This assumes that the Bayesian score follows a normal distribution and is different from the prediction using a cutoff.
Enrichment: An estimate of enrichment, that is, the increased likelihood (versus random) of this sample being in the category.
Bayesian Score: The standard Laplacian-modified Bayesian score.
Mahalanobis Distance: The Mahalanobis distance (MD) is the distance to the center of the training data. The larger the MD, the less trustworthy the prediction.
Mahalanobis Distance p-value: The p-value gives the fraction of training data with an MD greater than or equal to the one for the given sample, assuming normally distributed data. The smaller the p-value, the less trustworthy the prediciton. For highly non-normal X properties (e.g., fingerprints), the MD p-value is wildly inaccurate.

## Model Applicability

Unknown features are fingerprint features in the query molecule, but not found or appearing too infrequently in the training set.

1. All properties and OPS components are within expected ranges.
2. Unknown ECFP_2 feature: 1310940530: [*]C([*])OC(=[*])[*]
3. Unknown ECFP_2 feature: 1795218697: [*]OC(=C([*])[*])C(=[*])[*]
4. Unknown ECFP_2 feature: 1796421070: [*]OC(=C([*])[*])[c](:[*]):[*]

## Feature Contribution

### Top features for positive contribution

| Fingerprint | Bit/Smiles | Feature Structure | Score | Carcinogen in training set |
|---|---|---|---|---|
| ECFP_6 | 1419645508 | 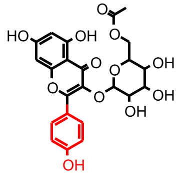 [*][c]1:[cH]:[cH]:[c](O):[cH]:[cH]:1 | 0.675 | 4 out of 5 |

| | | | | |
|---|---|---|---|---|
| ECFP_6 | -790637051 | 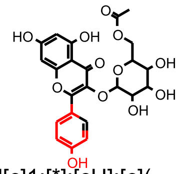 [*][c]1:[*]:[cH]:[c](O):[cH]:[cH]:1 | 0.615 | 6 out of 9 |
| ECFP_6 | 1740779540 | 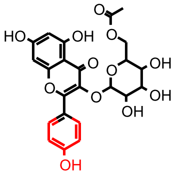 O[c]1:[cH]:[cH]:[*]:[cH]:[cH]:1 | 0.56 | 4 out of 6 |

**Top Features for negative contribution**

| Fingerprint | Bit/Smiles | Feature Structure | Score | Carcinogen in training set |
|---|---|---|---|---|
| ECFP_6 | 1717462980 | 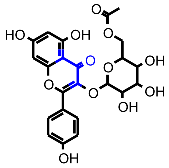 [*]C(=[*])C(=O)[c](:[*]):[*] | -1.25 | 0 out of 8 |
| ECFP_6 | -219423964 | 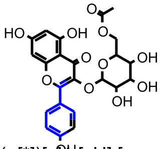 [*]C(=[*])[c]1:[cH]:[*]:[c]([*]):[cH]:[cH]:1 | -0.935 | 0 out of 5 |
| ECFP_6 | -2060414325 | 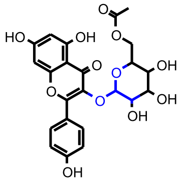 [*]OC(O[*])C([*])[*] | -0.657 | 0 out of 3 |

## Remdesivir

## TOPKAT_Mouse_Male_FDA_None_vs_Carcinogen

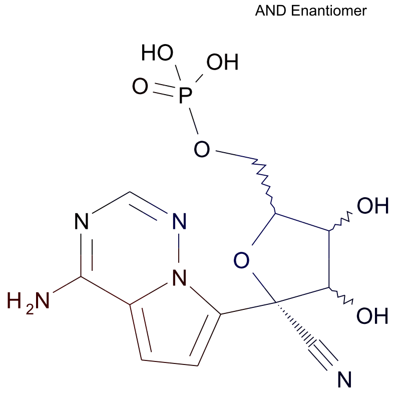

$C_{12}H_{14}N_5O_7P$
Molecular Weight: 371.24262
ALogP: -1.502
Rotatable Bonds: 4
Acceptors: 11
Donors: 5

## Model Prediction

Prediction: Non-Carcinogen
Probability: 0.239
Enrichment: 0.812
Bayesian Score: -2.82
Mahalanobis Distance: 19.2
Mahalanobis Distance p-value: 7.81e-017

Prediction: Positive if the Bayesian score is above the estimated best cutoff value from minimizing the false positive and false negative rate.
Probability: The esimated probability that the sample is in the positive category. This assumes that the Bayesian score follows a normal distribution and is different from the prediction using a cutoff.
Enrichment: An estimate of enrichment, that is, the increased likelihood (versus random) of this sample being in the category.
Bayesian Score: The standard Laplacian-modified Bayesian score.
Mahalanobis Distance: The Mahalanobis distance (MD) is the distance to the center of the training data. The larger the MD, the less trustworthy the prediction.
Mahalanobis Distance p-value: The p-value gives the fraction of training data with an MD greater than or equal to the one for the given sample, assuming normally distributed data. The smaller the p-value, the less trustworthy the prediciton. For highly non-normal X properties (e.g., fingerprints), the MD p-value is wildly inaccurate.

## Structural Similar Compounds

| Name | Famotidine | Tetracycline | Ribavirin |
|---|---|---|---|
| Structure | 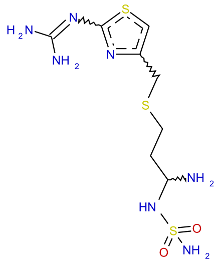 | 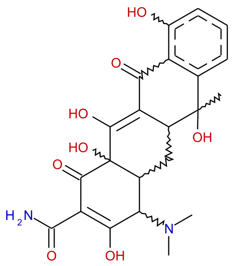 | 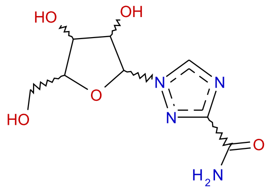 |
| Actual Endpoint | Non-Carcinogen | Non-Carcinogen | Non-Carcinogen |
| Predicted Endpoint | Non-Carcinogen | Non-Carcinogen | Non-Carcinogen |
| Distance | 0.813 | 0.843 | 0.860 |
| Reference | US FDA (Centre for Drug Eval.& Res./Off. Testing & Res.) Sept. 1997 | US FDA (Centre for Drug Eval.& Res./Off. Testing & Res.) Sept. 1997 | US FDA (Centre for Drug Eval.& Res./Off. Testing & Res.) Sept. 1997 |

## Model Applicability

Unknown features are fingerprint features in the query molecule, but not found or appearing too infrequently in the training set.

1. All properties and OPS components are within expected ranges.
2. Unknown FCFP_2 feature: 472180098: [*]OP(=O)(O)O
3. Unknown FCFP_2 feature: -332197802: [*][c]1:[*]:[*]:[c](:[*]):n:1:n:[*]

## Feature Contribution

**Top features for positive contribution**

| Fingerprint | Bit/Smiles | Feature Structure | Score | Carcinogen in training set |
|---|---|---|---|---|
| FCFP_6 | -450797925 | 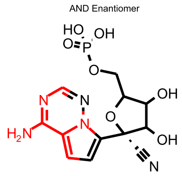 N[c]1:n:[cH]:[*]:n2:[ *]:[*]:[cH]:[c]:1:2 | 0.676 | 2 out of 2 |

| | | | | |
|---|---|---|---|---|
| FCFP_6 | -1151884458 | 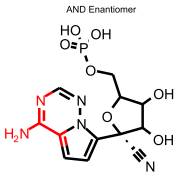<br>[*]:n:[c](N):[c](:[*]):[*] | 0.348 | 6 out of 15 |
| FCFP_6 | -1280036918 | 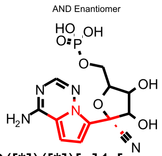<br>[*]C([*])([*])[c]1:[cH]:[cH]:[c](:[*]):n:1:[*] | 0.333 | 7 out of 18 |

## Top Features for negative contribution

| Fingerprint | Bit/Smiles | Feature Structure | Score | Carcinogen in training set |
|---|---|---|---|---|
| FCFP_6 | -124685461 | 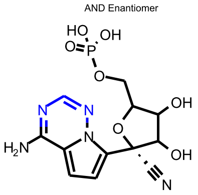<br>[*]:n:[cH]:n:[*] | -0.731 | 1 out of 12 |
| FCFP_6 | 422052003 | 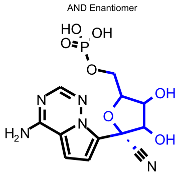<br>[*]CC1OC([*])([*])C(O)C1O | -0.582 | 0 out of 3 |
| FCFP_6 | -1277879912 | 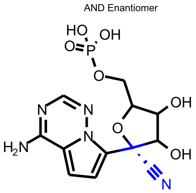<br>[*]C([*])([*])C#N | -0.582 | 0 out of 3 |

# Flavonoid

# TOPKAT_Mouse_Male_FDA_None_vs_Carcinogen

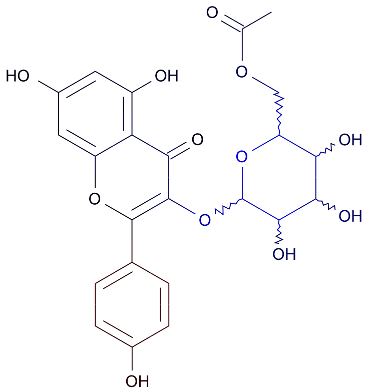

$C_{23}H_{22}O_{12}$

Molecular Weight: 490.41357

ALogP: 0.322

Rotatable Bonds: 6

Acceptors: 12

Donors: 6

## Model Prediction

Prediction: Non-Carcinogen

Probability: 0.154

Enrichment: 0.524

Bayesian Score: -8.56

Mahalanobis Distance: 14

Mahalanobis Distance p-value: 6.73e-006

Prediction: Positive if the Bayesian score is above the estimated best cutoff value from minimizing the false positive and false negative rate.
Probability: The esimated probability that the sample is in the positive category. This assumes that the Bayesian score follows a normal distribution and is different from the prediction using a cutoff.
Enrichment: An estimate of enrichment, that is, the increased likelihood (versus random) of this sample being in the category.
Bayesian Score: The standard Laplacian-modified Bayesian score.
Mahalanobis Distance: The Mahalanobis distance (MD) is the distance to the center of the training data. The larger the MD, the less trustworthy the prediction.
Mahalanobis Distance p-value: The p-value gives the fraction of training data with an MD greater than or equal to the one for the given sample, assuming normally distributed data. The smaller the p-value, the less trustworthy the prediciton. For highly non-normal X properties (e.g., fingerprints), the MD p-value is wildly inaccurate.

## Structural Similar Compounds

| Name | Daunorubicin | Methotrexate | Minocycline |
|---|---|---|---|
| Structure | 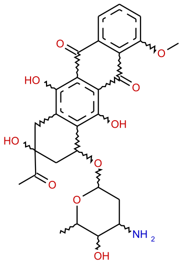 | 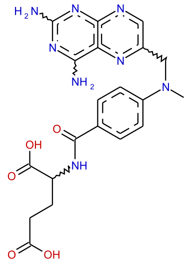 | 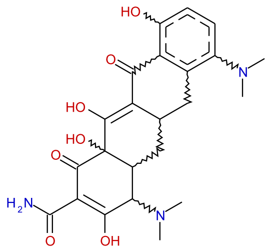 |
| Actual Endpoint | Non-Carcinogen | Carcinogen | Non-Carcinogen |
| Predicted Endpoint | Non-Carcinogen | Carcinogen | Non-Carcinogen |
| Distance | 0.638 | 0.700 | 0.797 |
| Reference | US FDA (Centre for Drug Eval.& Res./Off. Testing & Res.) Sept. 1997 | US FDA (Centre for Drug Eval.& Res./Off. Testing & Res.) Sept. 1997 | US FDA (Centre for Drug Eval.& Res./Off. Testing & Res.) Sept. 1997 |

## Model Applicability

Unknown features are fingerprint features in the query molecule, but not found or appearing too infrequently in the training set.

1. All properties and OPS components are within expected ranges.
2. Unknown FCFP_2 feature: -1678245750: [*]OC(=C([*])[*])[c](:[*]):[*]

## Feature Contribution

**Top features for positive contribution**

| Fingerprint | Bit/Smiles | Feature Structure | Score | Carcinogen in training set |
|---|---|---|---|---|
| FCFP_6 | -1066794953 | 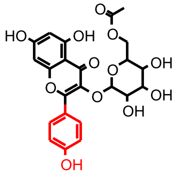 [*][c]1:[cH]:[cH]:[c](O):[cH]:[cH]:1 | 0.668 | 3 out of 4 |

| | | | | |
|---|---|---|---|---|
| FCFP_6 | -1847351220 | 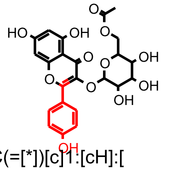 [*]C(=[*])[c]1:[cH]:[cH]:[c](O):[cH]:[cH]:1 | 0.547 | 3 out of 5 |
| FCFP_6 | -158888774 | 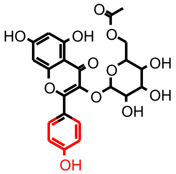 O[c]1:[cH]:[cH]:[*]:[cH]:[cH]:1 | 0.367 | 5 out of 12 |
| **Top Features for negative contribution** | | | | |
| **Fingerprint** | **Bit/Smiles** | **Feature Structure** | **Score** | **Carcinogen in training set** |
| FCFP_6 | 699500266 | 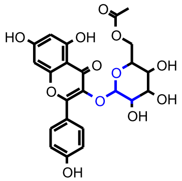 [*]OC(O[*])C([*])[*] | -0.85 | 1 out of 14 |
| FCFP_6 | -333501072 | 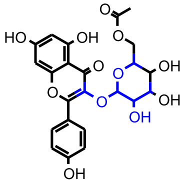 [*]C1[*]C([*])C(O)C(OC(=[*])[*])O1 | -0.582 | 0 out of 3 |
| FCFP_6 | 422052003 | 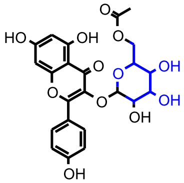 [*]CC1OC([*])([*])C(O)C1O | -0.582 | 0 out of 3 |

# Remdesivir

# TOPKAT_Ocular_Irritancy_Mild_vs_Moderate_Severe

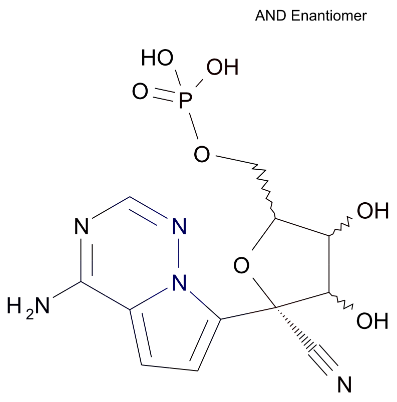


$C_{12}H_{14}N_5O_7P$

Molecular Weight: 371.24262

ALogP: -1.502

Rotatable Bonds: 4

Acceptors: 11

Donors: 5

## Structural Similar Compounds

| Name | 1;3;6-NAPHTHALENE TRISULFONIC ACID;7-AMINO- | Methanol; (s-triazine-2;4;6-triyltrinitrilo)hexa- | 2;2'-Biphenyldisulfonic acid; 4;4'-diamino- |
|---|---|---|---|
| Structure | 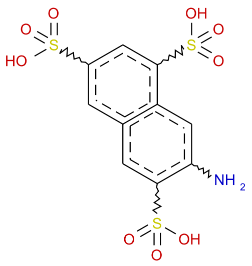 | 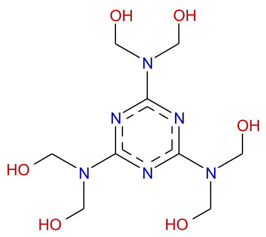 | 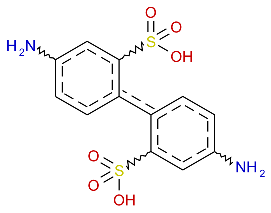 |
| Actual Endpoint | Mild | Moderate_Severe | Mild |
| Predicted Endpoint | Mild | Moderate_Severe | Mild |
| Distance | 0.776 | 0.802 | 0.878 |
| Reference | 28ZPAK-;190;72 | Prehled Prumyslove Toxikologie; Organicke Latky; Marhold; J. -;876;86 | Prehled Prumyslove Toxikologie; Organicke Latky; Marhold; J. pp 1061;86 |

## Model Prediction

Prediction: Mild

Probability: 0.789

Enrichment: 1.15

Bayesian Score: -1.39

Mahalanobis Distance: 13.8

Mahalanobis Distance p-value: 1.42e-009

Prediction: Positive if the Bayesian score is above the estimated best cutoff value from minimizing the false positive and false negative rate.
Probability: The esimated probability that the sample is in the positive category. This assumes that the Bayesian score follows a normal distribution and is different from the prediction using a cutoff.
Enrichment: An estimate of enrichment, that is, the increased likelihood (versus random) of this sample being in the category.
Bayesian Score: The standard Laplacian-modified Bayesian score.
Mahalanobis Distance: The Mahalanobis distance (MD) is the distance to the center of the training data. The larger the MD, the less trustworthy the prediction.
Mahalanobis Distance p-value: The p-value gives the fraction of training data with an MD greater than or equal to the one for the given sample, assuming normally distributed data. The smaller the p-value, the less trustworthy the prediciton. For highly non-normal X properties (e.g., fingerprints), the MD p-value is wildly inaccurate.

## Model Applicability

Unknown features are fingerprint features in the query molecule, but not found or appearing too infrequently in the training set.

1. OPS PC17 out of range. Value: 4.6782. Training min, max, SD, explained variance: -4.348, 3.9505, 1.094, 0.0146.
2. Unknown FCFP_2 feature: 472180098: [*]OP(=O)(O)O
3. Unknown FCFP_2 feature: -836603894: [*]C1[*][*]O[C@]1(C#[*])[c](:[*]):[*]
4. Unknown FCFP_2 feature: -124685461: [*]:n:[cH]:n:[*]
5. Unknown FCFP_2 feature: -1151884458: [*]:n:[c](N):[c](:[*]):[*]

## Feature Contribution

**Top features for positive contribution**

| Fingerprint | Bit/Smiles | Feature Structure | Score | Moderate_Severe in training set |
|---|---|---|---|---|

| | | | | |
|---|---|---|---|---|
| FCFP_10 | 1070061035 | 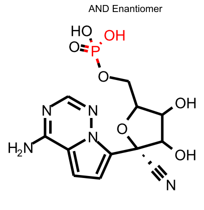 [*]P(=[*])([*])O | 0.239 | 284 out of 338 |
| FCFP_10 | -1539132615 | 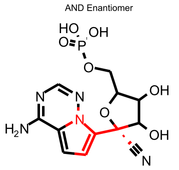 [*]C([*])([*])[c]1:[cH]:[*]:[*]:n:1:[*] | 0.224 | 11 out of 13 |
| FCFP_10 | -1043250487 | 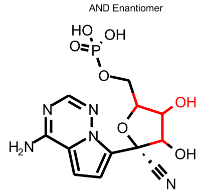 [*]C1[*][*]C([*])C1O | 0.22 | 62 out of 75 |

**Top Features for negative contribution**

| **Fingerprint** | **Bit/Smiles** | **Feature Structure** | **Score** | **Moderate_Severe in training set** |
|---|---|---|---|---|
| FCFP_10 | 4427049 | 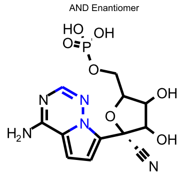 [*]:[cH]:n:n(:[*]):[*] | -1.29 | 0 out of 4 |
| FCFP_10 | -332197802 | 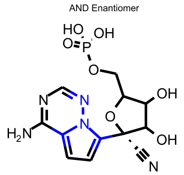 [*][c]1:[*]:[*]:[c](:[*]):n:1:n:[*] | -0.507 | 0 out of 1 |

| | | | | |
|---|---|---|---|---|
| FCFP_10 | 713358128 | 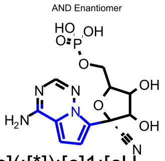[*][c](:[*]):[c]1:[cH]:[cH]:[c]([*]):n:1:[*] | -0.307 | 8 out of 17 |

# Flavonoid

# TOPKAT_Ocular_Irritancy_Mild_vs_Moderate_Severe

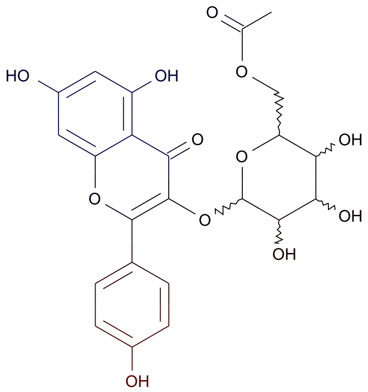

$C_{23}H_{22}O_{12}$

Molecular Weight: 490.41357

ALogP: 0.322

Rotatable Bonds: 6

Acceptors: 12

Donors: 6

## Structural Similar Compounds

| Name | Methanol; (s-triazine-2;4;6-triyltrinitrilo)hexa- | 2-Naphthalenesulfonic acid; 5;6'-iminobis(1-hydroxy- | 1;3;6-NAPHTHALENE TRISULFONIC ACID;7-AMINO- |
|---|---|---|---|
| Structure | 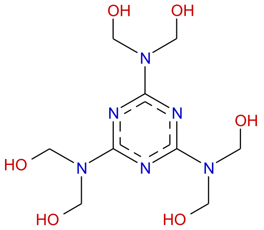 | 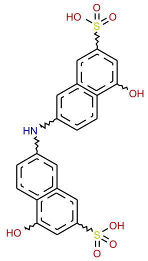 | 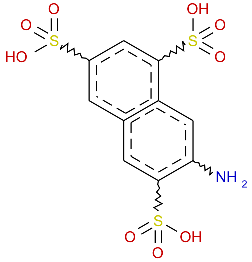 |
| Actual Endpoint | Moderate_Severe | Mild | Mild |
| Predicted Endpoint | Moderate_Severe | Mild | Mild |
| Distance | 0.862 | 0.944 | 1.115 |
| Reference | Prehled Prumyslove Toxikologie; Organicke Latky; Marhold; J. -;876;86 | Prehled Prumyslove Toxikologie; Organicke Latky; Marhold; J. pp 1065;86 | 28ZPAK-;190;72 |

## Model Prediction

Prediction: Moderate_Severe

Probability: 0.817

Enrichment: 1.19

Bayesian Score: -0.257

Mahalanobis Distance: 14

Mahalanobis Distance p-value: 4.67e-010

Prediction: Positive if the Bayesian score is above the estimated best cutoff value from minimizing the false positive and false negative rate.
Probability: The esimated probability that the sample is in the positive category. This assumes that the Bayesian score follows a normal distribution and is different from the prediction using a cutoff.
Enrichment: An estimate of enrichment, that is, the increased likelihood (versus random) of this sample being in the category.
Bayesian Score: The standard Laplacian-modified Bayesian score.
Mahalanobis Distance: The Mahalanobis distance (MD) is the distance to the center of the training data. The larger the MD, the less trustworthy the prediction.
Mahalanobis Distance p-value: The p-value gives the fraction of training data with an MD greater than or equal to the one for the given sample, assuming normally distributed data. The smaller the p-value, the less trustworthy the prediciton. For highly non-normal X properties (e.g., fingerprints), the MD p-value is wildly inaccurate.

## Model Applicability

Unknown features are fingerprint features in the query molecule, but not found or appearing too infrequently in the training set.

1. All properties and OPS components are within expected ranges.
2. Unknown FCFP_2 feature: -1678245750: [*]OC(=C([*])[*])[c](:[*]):[*]

## Feature Contribution

### Top features for positive contribution

| Fingerprint | Bit/Smiles | Feature Structure | Score | Moderate_Severe in training set |
|---|---|---|---|---|
| FCFP_10 | -1066794953 | 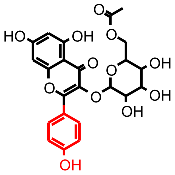 [*][c]1:[cH]:[cH]:[c](O):[cH]:[cH]:1 | 0.378 | 13 out of 13 |

| | | | | |
|---|---|---|---|---|
| FCFP_10 | -158888774 | 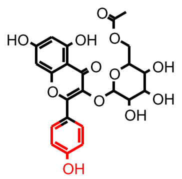 O[c]1:[cH]:[cH]:[*]:[cH]:[cH]:1 | 0.356 | 24 out of 25 |
| FCFP_10 | -1847351220 | 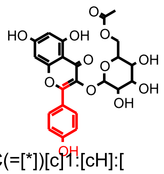 [*]C(=[*])[c]1:[cH]:[cH]:[c](O):[cH]:[cH]:1 | 0.273 | 9 out of 10 |
| **Top Features for negative contribution** | | | | |
| **Fingerprint** | **Bit/Smiles** | **Feature Structure** | **Score** | **Moderate_Severe in training set** |
| FCFP_10 | 115228054 | 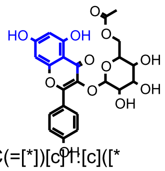 [*]C(=[*])[c]1:[c]([*]):[cH]:[c](O):[cH]:[c]:1O | -0.507 | 0 out of 1 |
| FCFP_10 | 946068634 | 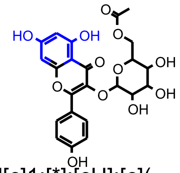 [*][c]1:[*]:[cH]:[c](O):[cH]:[c]:1O | -0.4 | 1 out of 3 |
| FCFP_10 | -1099193755 | 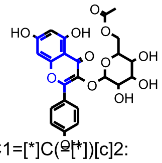 [*]C1=[*]C(=[*])[c]2:[c]([*]):[*]:[c]([*]):[cH]:[c]:2O1 | -0.361 | 2 out of 5 |

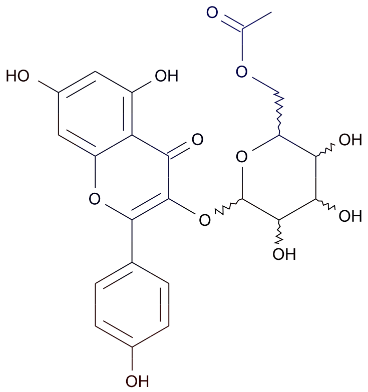

$C_{23}H_{22}O_{12}$
Molecular Weight: 490.41357
ALogP: 0.322
Rotatable Bonds: 6
Acceptors: 12
Donors: 6

## Structural Similar Compounds

| Name | Methanol; (s-triazine-2;4;6-triyltrinitrilo)hexa- | 2;7-NAPHTHALENE DISULFONIC ACID;4-AMINO-5-HYDROXY-;P-TOLUENE SULFONATE (ESTER) | ANTHRAQUINONE;1;5-DIAMINO-4;8-DIHYDROXY- |
|---|---|---|---|
| Structure | 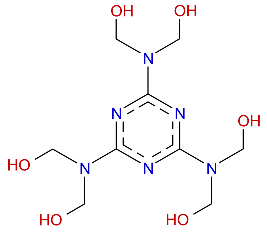 | 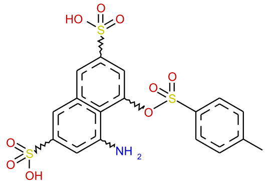 | 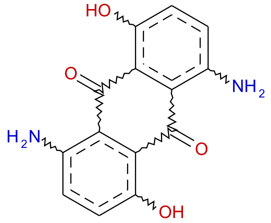 |
| Actual Endpoint | Moderate | Moderate | Moderate |
| Predicted Endpoint | Moderate | Moderate | Moderate |
| Distance | 0.870 | 1.251 | 1.294 |
| Reference | Prehled Prumyslove Toxikologie; Organicke Latky; Marhold; J. -;876;86 | 28ZPAK-;194;72 | 28ZPAK-;103;72 |

## Model Prediction

Prediction: Moderate
Probability: 0.562
Enrichment: 0.907
Bayesian Score: -3
Mahalanobis Distance: 15
Mahalanobis Distance p-value: 4.87e-014

Prediction: Positive if the Bayesian score is above the estimated best cutoff value from minimizing the false positive and false negative rate.
Probability: The esimated probability that the sample is in the positive category. This assumes that the Bayesian score follows a normal distribution and is different from the prediction using a cutoff.
Enrichment: An estimate of enrichment, that is, the increased likelihood (versus random) of this sample being in the category.
Bayesian Score: The standard Laplacian-modified Bayesian score.
Mahalanobis Distance: The Mahalanobis distance (MD) is the distance to the center of the training data. The larger the MD, the less trustworthy the prediction.
Mahalanobis Distance p-value: The p-value gives the fraction of training data with an MD greater than or equal to the one for the given sample, assuming normally distributed data. The smaller the p-value, the less trustworthy the prediciton. For highly non-normal X properties (e.g., fingerprints), the MD p-value is wildly inaccurate.

## Model Applicability

Unknown features are fingerprint features in the query molecule, but not found or appearing too infrequently in the training set.

1. All properties and OPS components are within expected ranges.

## Feature Contribution

### Top features for positive contribution

| Fingerprint | Bit/Smiles | Feature Structure | Score | Severe in training set |
|---|---|---|---|---|
| SCFP_12 | 130348166 | 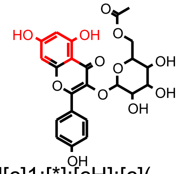 [*][c]1:[*]:[cH]:[c](O):[cH]:[c]:1O | 0.376 | 4 out of 4 |

| | | | | |
|---|---|---|---|---|
| SCFP_12 | 611156666 | 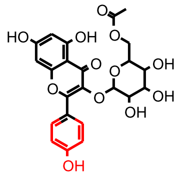 O[c]1:[cH]:[cH]:[*]:[cH]:[cH]:1 | 0.298 | 20 out of 24 |
| SCFP_12 | 1112262477 | 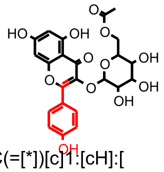 [*]C(=[*])[c]1:[cH]:[cH]:[c](O):[cH]:[cH]:1 | 0.218 | 1 out of 1 |

**Top Features for negative contribution**

| Fingerprint | Bit/Smiles | Feature Structure | Score | Severe in training set |
|---|---|---|---|---|
| SCFP_12 | -1031053674 | 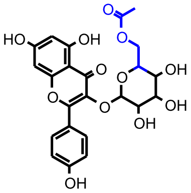 [*]C([*])COC(=O)C | -0.874 | 5 out of 22 |
| SCFP_12 | 860600739 | 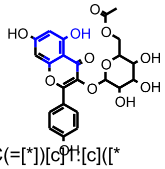 [*]C(=[*])[c]1:[c]([*]):[*]:[c]([*]):[cH]:[c]:1O | -0.703 | 1 out of 5 |
| SCFP_12 | 616636418 | 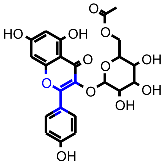 [*]OC(=C([*])[*])[c](:[*]):[*] | -0.475 | 0 out of 1 |

# Remdesivir

# TOPKAT_Ocular_Irritancy_None_vs_Irritant

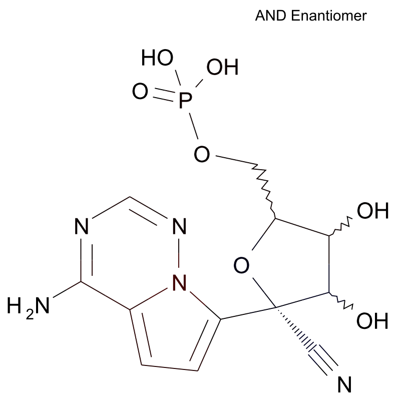


$C_{12}H_{14}N_5O_7P$

Molecular Weight: 371.24262

ALogP: -1.502

Rotatable Bonds: 4

Acceptors: 11

Donors: 5

## Structural Similar Compounds

| Name | 1;3;6-NAPHTHALENE TRISULFONIC ACID;7-AMINO- | Methanol; (s-triazine-2;4;6-triyltrinitrilo)hexa- | 2;2'-Biphenyldisulfonic acid; 4;4'-diamino- |
|---|---|---|---|
| Structure | 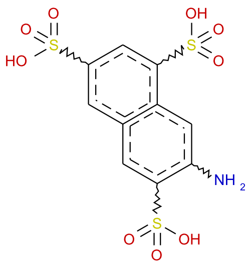 | 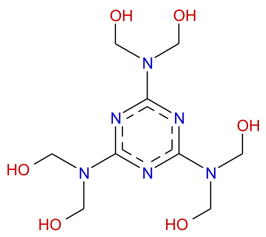 | 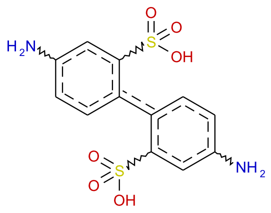 |
| Actual Endpoint | Irritant | Irritant | Irritant |
| Predicted Endpoint | Irritant | Irritant | Irritant |
| Distance | 0.766 | 0.795 | 0.859 |
| Reference | 28ZPAK-;190;72 | Prehled Prumyslove Toxikologie; Organicke Latky; Marhold; J. -;876;86 | Prehled Prumyslove Toxikologie; Organicke Latky; Marhold; J. pp 1061;86 |

## Model Prediction

Prediction: Irritant

Probability: 1

Enrichment: 1.18

Bayesian Score: 1.33

Mahalanobis Distance: 10.7

Mahalanobis Distance p-value: 0.0147

Prediction: Positive if the Bayesian score is above the estimated best cutoff value from minimizing the false positive and false negative rate.
Probability: The esimated probability that the sample is in the positive category. This assumes that the Bayesian score follows a normal distribution and is different from the prediction using a cutoff.
Enrichment: An estimate of enrichment, that is, the increased likelihood (versus random) of this sample being in the category.
Bayesian Score: The standard Laplacian-modified Bayesian score.
Mahalanobis Distance: The Mahalanobis distance (MD) is the distance to the center of the training data. The larger the MD, the less trustworthy the prediction.
Mahalanobis Distance p-value: The p-value gives the fraction of training data with an MD greater than or equal to the one for the given sample, assuming normally distributed data. The smaller the p-value, the less trustworthy the prediciton. For highly non-normal X properties (e.g., fingerprints), the MD p-value is wildly inaccurate.

## Model Applicability

Unknown features are fingerprint features in the query molecule, but not found or appearing too infrequently in the training set.

1. All properties and OPS components are within expected ranges.
2. Unknown FCFP_2 feature: 472180098: [*]OP(=O)(O)O
3. Unknown FCFP_2 feature: -124685461: [*]:n:[cH]:n:[*]
4. Unknown FCFP_2 feature: -1151884458: [*]:n:[c](N):[c](:[*]):[*]

## Feature Contribution

**Top features for positive contribution**

| Fingerprint | Bit/Smiles | Feature Structure | Score | Irritant in training set |
|---|---|---|---|---|

| | | | | |
|---|---|---|---|---|
| FCFP_12 | 1747237384 | 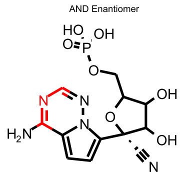 [*][c](:[*]):n:[cH]:[*] | 0.208 | 44 out of 44 |
| FCFP_12 | 178336375 | 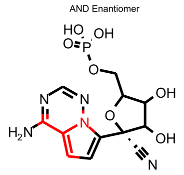 [*][c](:[*]):[c]1:[cH]:[*]:[*]:n:1:[*] | 0.202 | 19 out of 19 |
| FCFP_12 | 713358128 | 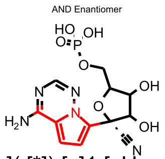 [*][c](:[*]):[c]1:[cH]:[cH]:[c]([*]):n:1:[*] | 0.2 | 17 out of 17 |
| **Top Features for negative contribution** | | | | |
| **Fingerprint** | **Bit/Smiles** | **Feature Structure** | **Score** | **Irritant in training set** |
| FCFP_12 | -836603894 | 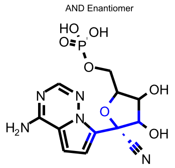 [*]C1[*][*]O[C@]1(C#[*])[c](:[*]):[*] | -0.592 | 0 out of 1 |
| FCFP_12 | -1277879912 | 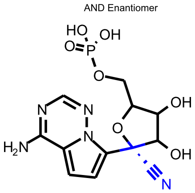 [*]C([*])([*])C#N | -0.0939 | 33 out of 45 |

| | | | | |
|---|---|---|---|---|
| FCFP_12 | 1872154524 | 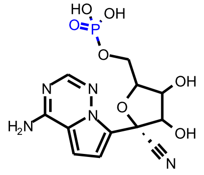 [*]P(=O)([*])[*] | 0 | 563 out of 690 |

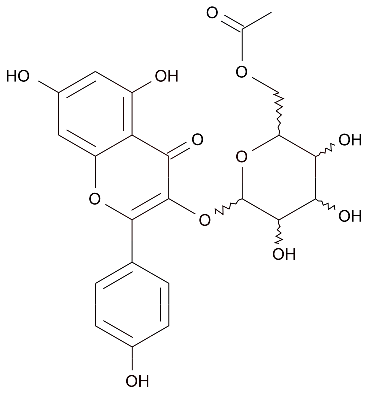


$C_{23}H_{22}O_{12}$
Molecular Weight: 490.41357
ALogP: 0.322
Rotatable Bonds: 6
Acceptors: 12
Donors: 6

## Structural Similar Compounds

| Name | Methanol; (s-triazine-2;4;6-triyltrinitrilo)hexa- | 2-Naphthalenesulfonic acid; 5;6'-iminobis(1-hydroxy- | 1;3;6-NAPHTHALENE TRISULFONIC ACID;7-AMINO- |
|---|---|---|---|
| Structure | 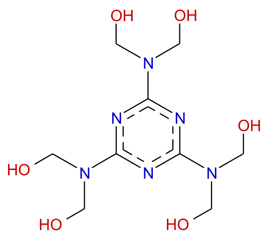 | 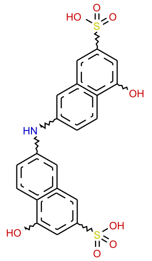 | 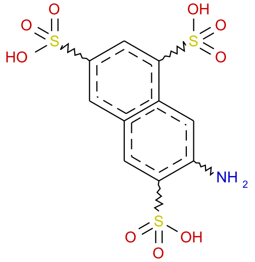 |
| Actual Endpoint | Irritant | Irritant | Irritant |
| Predicted Endpoint | Irritant | Irritant | Irritant |
| Distance | 0.839 | 0.915 | 1.113 |
| Reference | Prehled Prumyslove Toxikologie; Organicke Latky; Marhold; J. -;876;86 | Prehled Prumyslove Toxikologie; Organicke Latky; Marhold; J. pp 1065;86 | 28ZPAK-;190;72 |

## Model Prediction

Prediction: Irritant
Probability: 1
Enrichment: 1.18
Bayesian Score: 1.54
Mahalanobis Distance: 13.6
Mahalanobis Distance p-value: 3.14e-009

Prediction: Positive if the Bayesian score is above the estimated best cutoff value from minimizing the false positive and false negative rate.
Probability: The esimated probability that the sample is in the positive category. This assumes that the Bayesian score follows a normal distribution and is different from the prediction using a cutoff.
Enrichment: An estimate of enrichment, that is, the increased likelihood (versus random) of this sample being in the category.
Bayesian Score: The standard Laplacian-modified Bayesian score.
Mahalanobis Distance: The Mahalanobis distance (MD) is the distance to the center of the training data. The larger the MD, the less trustworthy the prediction.
Mahalanobis Distance p-value: The p-value gives the fraction of training data with an MD greater than or equal to the one for the given sample, assuming normally distributed data. The smaller the p-value, the less trustworthy the prediciton. For highly non-normal X properties (e.g., fingerprints), the MD p-value is wildly inaccurate.

## Model Applicability

Unknown features are fingerprint features in the query molecule, but not found or appearing too infrequently in the training set.

1. All properties and OPS components are within expected ranges.
2. Unknown FCFP_2 feature: -1678245750: [*]OC(=C([*])[*])[c](:[*]):[*]

## Feature Contribution

**Top features for positive contribution**

| Fingerprint | Bit/Smiles | Feature Structure | Score | Irritant in training set |
|---|---|---|---|---|
| FCFP_12 | -1099193755 | 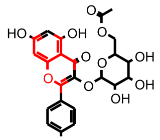 [*]C1=[*]C(=[*])[c]2:[c]([*]):[*]:[c]([*]):[cH]:[c]:2O1 | 0.175 | 5 out of 5 |

| | | | | |
|---|---|---|---|---|
| FCFP_12 | 436915834 | 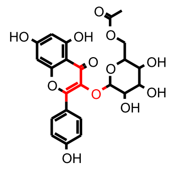<br>[*]OC(=C([*])[*])C(=[<br>*])[*] | 0.167 | 4 out of 4 |
| FCFP_12 | 946068634 | 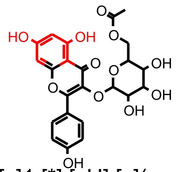<br>[*][c]1:[*]:[cH]:[c](<br>O):[cH]:[c]:1O | 0.156 | 3 out of 3 |
| **Top Features for negative contribution** | | | | |
| **Fingerprint** | **Bit/Smiles** | **Feature Structure** | **Score** | **Irritant in training set** |
| FCFP_12 | -432846198 | 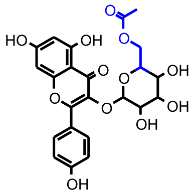<br>[*]C([*])COC(=O)C | -0.229 | 91 out of 142 |
| FCFP_12 | 1673930087 | 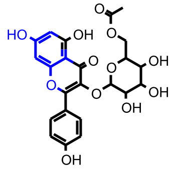<br>[*]O[c]1:[cH]:[c](O):<br>[cH]:[*]:[c]:1[*] | -0.218 | 5 out of 8 |
| FCFP_12 | 565998553 | 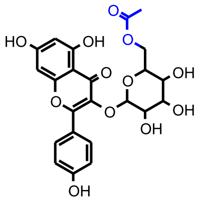<br>[*]OC(=O)C | -0.0662 | 198 out of 262 |

# Remdesivir

# TOPKAT_Rat_Female_FDA_None_vs_Carcinogen

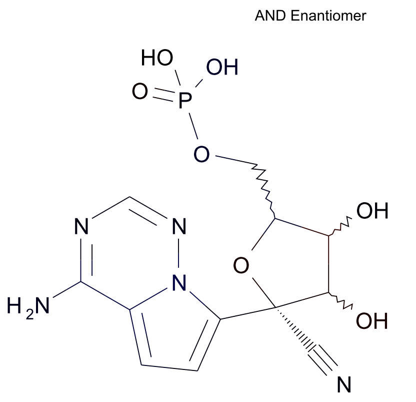


$C_{12}H_{14}N_5O_7P$

Molecular Weight: 371.24262

ALogP: -1.502

Rotatable Bonds: 4

Acceptors: 11

Donors: 5

## Structural Similar Compounds

| Name | Streptozocin | Tetracycline | Famotidine |
|---|---|---|---|
| Structure | 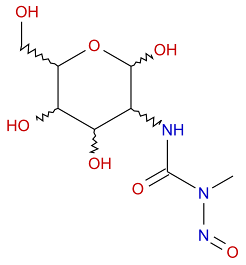 | 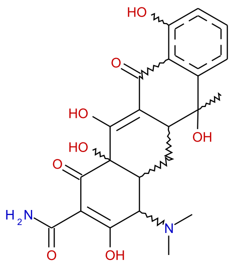 | 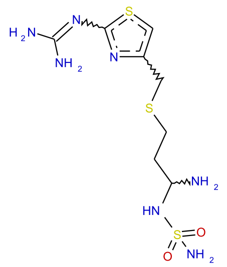 |
| Actual Endpoint | Carcinogen | Non-Carcinogen | Non-Carcinogen |
| Predicted Endpoint | Carcinogen | Non-Carcinogen | Non-Carcinogen |
| Distance | 0.810 | 0.858 | 0.861 |
| Reference | US FDA (Centre for Drug Eval.& Res./Off. Testing & Res.) Sept. 1997 | US FDA (Centre for Drug Eval.& Res./Off. Testing & Res.) Sept. 1997 | US FDA (Centre for Drug Eval.& Res./Off. Testing & Res.) Sept. 1997 |

## Model Prediction

Prediction: Non-Carcinogen

Probability: 0.243

Enrichment: 0.756

Bayesian Score: -3.24

Mahalanobis Distance: 13.8

Mahalanobis Distance p-value: 5.04e-006

Prediction: Positive if the Bayesian score is above the estimated best cutoff value from minimizing the false positive and false negative rate.
Probability: The esimated probability that the sample is in the positive category. This assumes that the Bayesian score follows a normal distribution and is different from the prediction using a cutoff.
Enrichment: An estimate of enrichment, that is, the increased likelihood (versus random) of this sample being in the category.
Bayesian Score: The standard Laplacian-modified Bayesian score.
Mahalanobis Distance: The Mahalanobis distance (MD) is the distance to the center of the training data. The larger the MD, the less trustworthy the prediction.
Mahalanobis Distance p-value: The p-value gives the fraction of training data with an MD greater than or equal to the one for the given sample, assuming normally distributed data. The smaller the p-value, the less trustworthy the prediciton. For highly non-normal X properties (e.g., fingerprints), the MD p-value is wildly inaccurate.

## Model Applicability

Unknown features are fingerprint features in the query molecule, but not found or appearing too infrequently in the training set.

1. All properties and OPS components are within expected ranges.
2. Unknown ECFP_2 feature: 1126642748: [*]OP(=O)(O)O
3. Unknown ECFP_2 feature: -1250439909: [*]COP(=[*])([*])[*]
4. Unknown ECFP_2 feature: 1258791451: [*]C1[*][*]O[C@]1(C#[*])[c](:[*]):[*]
5. Unknown ECFP_2 feature: -1507082173: [*][c]1:[*]:[*]:[c](:[*]):n:1:n:[*]
6. Unknown ECFP_2 feature: -66263742: [*]C([*])([*])[c]1:[cH]:[*]:[*]:n:1:[*]

## Feature Contribution

| Top features for positive contribution | | | | |
|---|---|---|---|---|
| Fingerprint | Bit/Smiles | Feature Structure | Score | Carcinogen in training set |

| | | | | |
|---|---|---|---|---|
| ECFP_12 | -553149446 | 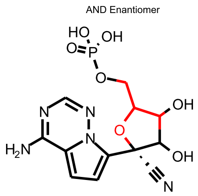 [*]CC1O[*][*]C1[*] | 0.575 | 3 out of 4 |
| ECFP_12 | -1114776580 | 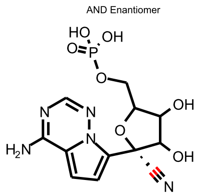 [*]C#[*] | 0.461 | 10 out of 19 |
| ECFP_12 | -521596699 | 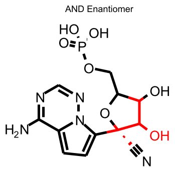 [*]C1[*][*]C([*])([*] )C1O | 0.445 | 3 out of 5 |
| **Top Features for negative contribution** | | | | |
| **Fingerprint** | **Bit/Smiles** | **Feature Structure** | **Score** | **Carcinogen in training set** |
| ECFP_12 | 2024329577 | 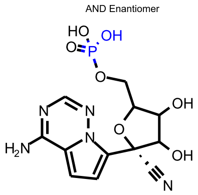 [*]P(=[*])([*])O | -0.661 | 0 out of 3 |
| ECFP_12 | -1687549011 | 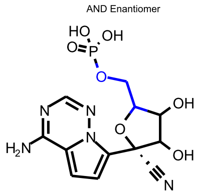 [*]OCC([*])[*] | -0.661 | 0 out of 3 |

| | | | | |
|---|---|---|---|---|
| ECFP_12 | -1734834311 | 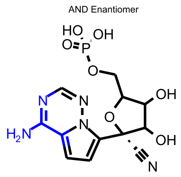 [*]:n:[c](N):[c](:[*]):[*] | -0.56 | 1 out of 8 |

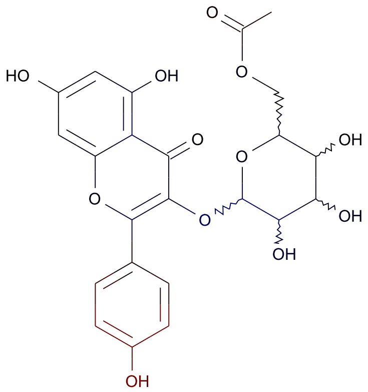

$C_{23}H_{22}O_{12}$
Molecular Weight: 490.41357
ALogP: 0.322
Rotatable Bonds: 6
Acceptors: 12
Donors: 6

## Model Prediction

Prediction: Non-Carcinogen
Probability: 0.247
Enrichment: 0.767
Bayesian Score: -2.98
Mahalanobis Distance: 12.4
Mahalanobis Distance p-value: 0.00147

Prediction: Positive if the Bayesian score is above the estimated best cutoff value from minimizing the false positive and false negative rate.
Probability: The esimated probability that the sample is in the positive category. This assumes that the Bayesian score follows a normal distribution and is different from the prediction using a cutoff.
Enrichment: An estimate of enrichment, that is, the increased likelihood (versus random) of this sample being in the category.
Bayesian Score: The standard Laplacian-modified Bayesian score.
Mahalanobis Distance: The Mahalanobis distance (MD) is the distance to the center of the training data. The larger the MD, the less trustworthy the prediction.
Mahalanobis Distance p-value: The p-value gives the fraction of training data with an MD greater than or equal to the one for the given sample, assuming normally distributed data. The smaller the p-value, the less trustworthy the prediciton. For highly non-normal X properties (e.g., fingerprints), the MD p-value is wildly inaccurate.

## Structural Similar Compounds

| Name | Minocycline | Tetracycline | Netilmicin |
|---|---|---|---|
| Structure | 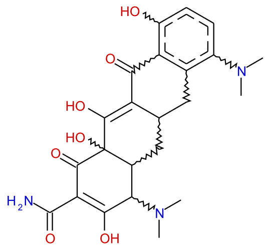 | 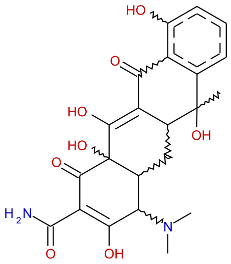 | 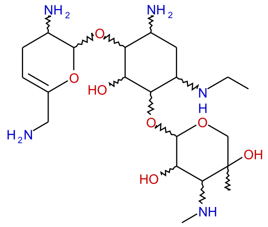 |
| Actual Endpoint | Carcinogen | Non-Carcinogen | Non-Carcinogen |
| Predicted Endpoint | Carcinogen | Non-Carcinogen | Non-Carcinogen |
| Distance | 0.838 | 0.855 | 0.981 |
| Reference | US FDA (Centre for Drug Eval.& Res./Off. Testing & Res.) Sept. 1997 | US FDA (Centre for Drug Eval.& Res./Off. Testing & Res.) Sept. 1997 | US FDA (Centre for Drug Eval.& Res./Off. Testing & Res.) Sept. 1997 |

## Model Applicability

Unknown features are fingerprint features in the query molecule, but not found or appearing too infrequently in the training set.

1. All properties and OPS components are within expected ranges.
2. Unknown ECFP_2 feature: 1310940530: [*]C([*])OC(=[*])[*]
3. Unknown ECFP_2 feature: 1795218697: [*]OC(=C([*])[*])C(=[*])[*]

## Feature Contribution

**Top features for positive contribution**

| Fingerprint | Bit/Smiles | Feature Structure | Score | Carcinogen in training set |
|---|---|---|---|---|
| ECFP_12 | 1419645508 | 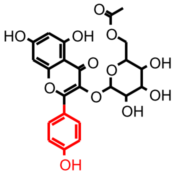 [*][c]1:[cH]:[cH]:[c](O):[cH]:[cH]:1 | 0.736 | 5 out of 6 |

| | | | | |
|---|---|---|---|---|
| ECFP_12 | 1740779540 | 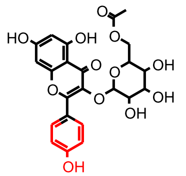 O[c]1:[cH]:[cH]:[*]:[cH]:[cH]:1 | 0.633 | 5 out of 7 |
| ECFP_12 | -553149446 | 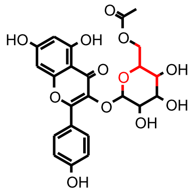 [*]CC1O[*][*]C1[*] | 0.575 | 3 out of 4 |

**Top Features for negative contribution**

| Fingerprint | Bit/Smiles | Feature Structure | Score | Carcinogen in training set |
|---|---|---|---|---|
| ECFP_12 | -2060414325 | 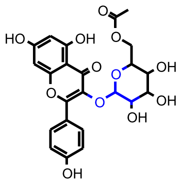 [*]OC(O[*])C([*])[*] | -0.811 | 0 out of 4 |
| ECFP_12 | -1687549011 | 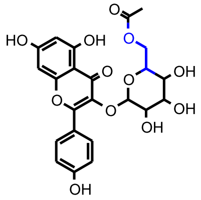 [*]OCC([*])[*] | -0.661 | 0 out of 3 |
| ECFP_12 | -181568884 | 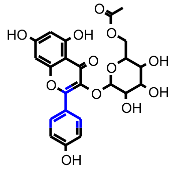 [*]C(=[*])[c](:[cH]:[*]):[cH]:[*] | -0.505 | 3 out of 18 |

# Remdesivir

# TOPKAT_Rat_Male_FDA_None_vs_Carcinogen

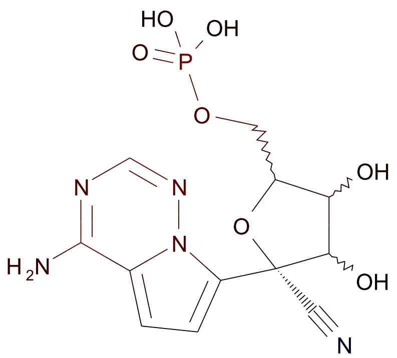


$C_{12}H_{14}N_5O_7P$
Molecular Weight: 371.24262
ALogP: -1.502
Rotatable Bonds: 4
Acceptors: 11
Donors: 5

## Model Prediction

Prediction: Carcinogen
Probability: 0.481
Enrichment: 1.44
Bayesian Score: 3.82
Mahalanobis Distance: 14.1
Mahalanobis Distance p-value: 3.32e-005

Prediction: Positive if the Bayesian score is above the estimated best cutoff value from minimizing the false positive and false negative rate.
Probability: The esimated probability that the sample is in the positive category. This assumes that the Bayesian score follows a normal distribution and is different from the prediction using a cutoff.
Enrichment: An estimate of enrichment, that is, the increased likelihood (versus random) of this sample being in the category.
Bayesian Score: The standard Laplacian-modified Bayesian score.
Mahalanobis Distance: The Mahalanobis distance (MD) is the distance to the center of the training data. The larger the MD, the less trustworthy the prediction.
Mahalanobis Distance p-value: The p-value gives the fraction of training data with an MD greater than or equal to the one for the given sample, assuming normally distributed data. The smaller the p-value, the less trustworthy the prediciton. For highly non-normal X properties (e.g., fingerprints), the MD p-value is wildly inaccurate.

## Structural Similar Compounds

| Name | Streptozocin | Famotidine | Tetracycline |
|---|---|---|---|
| Structure | 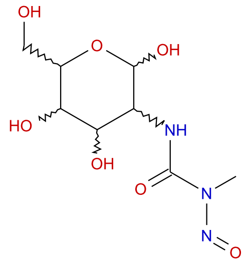 | 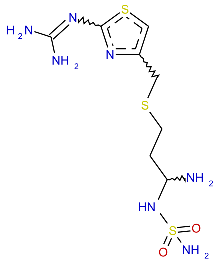 | 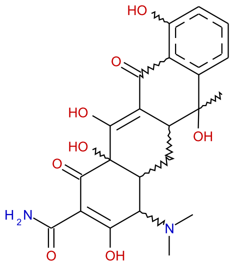 |
| Actual Endpoint | Carcinogen | Non-Carcinogen | Non-Carcinogen |
| Predicted Endpoint | Carcinogen | Non-Carcinogen | Non-Carcinogen |
| Distance | 0.789 | 0.850 | 0.856 |
| Reference | US FDA (Centre for Drug Eval.& Res./Off. Testing & Res.) Sept. 1997 | US FDA (Centre for Drug Eval.& Res./Off. Testing & Res.) Sept. 1997 | US FDA (Centre for Drug Eval.& Res./Off. Testing & Res.) Sept. 1997 |

## Model Applicability

Unknown features are fingerprint features in the query molecule, but not found or appearing too infrequently in the training set.

1. All properties and OPS components are within expected ranges.

## Feature Contribution

| Top features for positive contribution | | | | |
|---|---|---|---|---|
| **Fingerprint** | **Bit/Smiles** | **Feature Structure** | **Score** | **Carcinogen in training set** |
| SCFP_6 | -1029620989 | 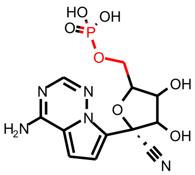  [*]COP(=[*])([*])[*] | 0.712 | 3 out of 3 |

| | | | | |
|---|---|---|---|---|
| SCFP_6 | 1245795878 | 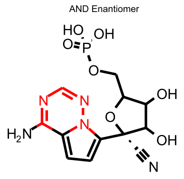  [*][c]1:[*]:n(:[*]):n :[cH]:n:1 | 0.603 | 2 out of 2 |
| SCFP_6 | 149212520 | 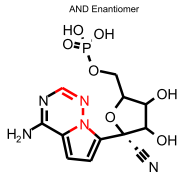  [*]:[cH]:n:n(:[*]):[* ] | 0.543 | 9 out of 15 |
| **Top Features for negative contribution** | | | | |
| **Fingerprint** | **Bit/Smiles** | **Feature Structure** | **Score** | **Carcinogen in training set** |
| SCFP_6 | -1019297400 | 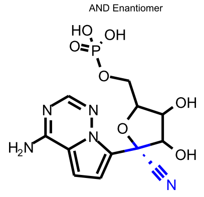  [*]C([*])([*])C#N | -0.674 | 0 out of 3 |
| SCFP_6 | 194135988 | 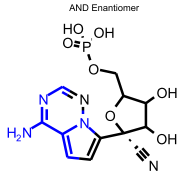  N[c]1:n:[cH]:[*]:n2:[ *]:[*]:[cH]:[c]:1:2 | -0.278 | 0 out of 1 |
| SCFP_6 | -424515134 | 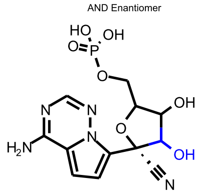  [*]C([*])O | -0.157 | 30 out of 110 |

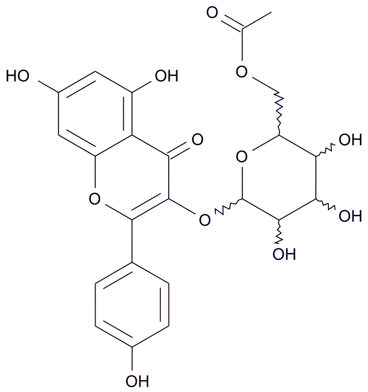

$C_{23}H_{22}O_{12}$
Molecular Weight: 490.41357
ALogP: 0.322
Rotatable Bonds: 6
Acceptors: 12
Donors: 6

## Model Prediction

Prediction: Non-Carcinogen
Probability: 0.287
Enrichment: 0.858
Bayesian Score: -2.69
Mahalanobis Distance: 11.6
Mahalanobis Distance p-value: 0.0665

Prediction: Positive if the Bayesian score is above the estimated best cutoff value from minimizing the false positive and false negative rate.
Probability: The esimated probability that the sample is in the positive category. This assumes that the Bayesian score follows a normal distribution and is different from the prediction using a cutoff.
Enrichment: An estimate of enrichment, that is, the increased likelihood (versus random) of this sample being in the category.
Bayesian Score: The standard Laplacian-modified Bayesian score.
Mahalanobis Distance: The Mahalanobis distance (MD) is the distance to the center of the training data. The larger the MD, the less trustworthy the prediction.
Mahalanobis Distance p-value: The p-value gives the fraction of training data with an MD greater than or equal to the one for the given sample, assuming normally distributed data. The smaller the p-value, the less trustworthy the prediciton. For highly non-normal X properties (e.g., fingerprints), the MD p-value is wildly inaccurate.

## Structural Similar Compounds

| Name | Minocycline | Tetracycline | Oxytetracycline |
|---|---|---|---|
| Structure | 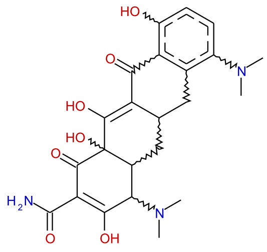 | 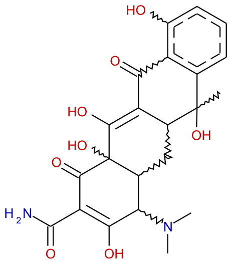 | 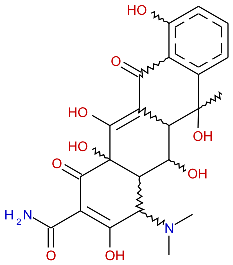 |
| Actual Endpoint | Carcinogen | Non-Carcinogen | Non-Carcinogen |
| Predicted Endpoint | Carcinogen | Non-Carcinogen | Non-Carcinogen |
| Distance | 0.809 | 0.826 | 0.832 |
| Reference | US FDA (Centre for Drug Eval.& Res./Off. Testing & Res.) Sept. 1997 | US FDA (Centre for Drug Eval.& Res./Off. Testing & Res.) Sept. 1997 | US FDA (Centre for Drug Eval.& Res./Off. Testing & Res.) Sept. 1997 |

## Model Applicability

Unknown features are fingerprint features in the query molecule, but not found or appearing too infrequently in the training set.

1. All properties and OPS components are within expected ranges.

## Feature Contribution

### Top features for positive contribution

| Fingerprint | Bit/Smiles | Feature Structure | Score | Carcinogen in training set |
|---|---|---|---|---|
| SCFP_6 | 1958008606 | 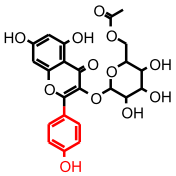 [*][c]1:[cH]:[cH]:[c](O):[cH]:[cH]:1 | 0.536 | 4 out of 6 |

| | | | | |
|---|---|---|---|---|
| SCFP_6 | -1715619483 | 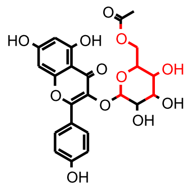 [*]OCC1OC([*])([*])C([*])C1O | 0.273 | 2 out of 4 |
| SCFP_6 | 276223760 | 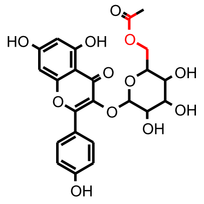 [*]COC(=[*])[*] | 0.243 | 24 out of 58 |

## Top Features for negative contribution

| **Fingerprint** | **Bit/Smiles** | **Feature Structure** | **Score** | **Carcinogen in training set** |
|---|---|---|---|---|
| SCFP_6 | -1031053674 | 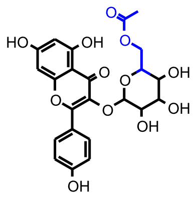 [*]C([*])COC(=O)C | -0.664 | 1 out of 9 |
| SCFP_6 | 616636418 | 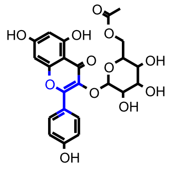 [*]OC(=C([*])[*])[c](:[*]):[*] | -0.278 | 0 out of 1 |
| SCFP_6 | 1433439981 | 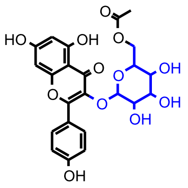 [*]CC1OC(O[*])C(O)C(O)C1O | -0.278 | 0 out of 1 |

# Remdesivir

# TOPKAT_Rat_Male_FDA_Single_vs_Multiple

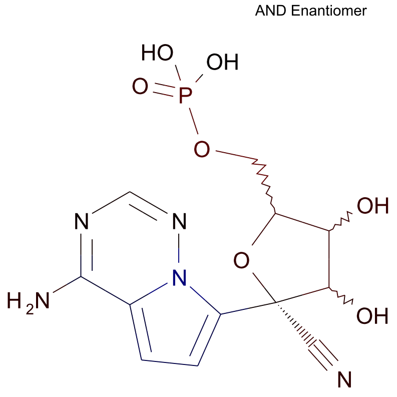


$C_{12}H_{14}N_5O_7P$
Molecular Weight: 371.24262
ALogP: -1.502
Rotatable Bonds: 4
Acceptors: 11
Donors: 5

## Model Prediction

Prediction: Multiple-Carcinogen
Probability: 0.556
Enrichment: 1.34
Bayesian Score: 3.52
Mahalanobis Distance: 14
Mahalanobis Distance p-value: 8.72e-005

Prediction: Positive if the Bayesian score is above the estimated best cutoff value from minimizing the false positive and false negative rate.
Probability: The esimated probability that the sample is in the positive category. This assumes that the Bayesian score follows a normal distribution and is different from the prediction using a cutoff.
Enrichment: An estimate of enrichment, that is, the increased likelihood (versus random) of this sample being in the category.
Bayesian Score: The standard Laplacian-modified Bayesian score.
Mahalanobis Distance: The Mahalanobis distance (MD) is the distance to the center of the training data. The larger the MD, the less trustworthy the prediction.
Mahalanobis Distance p-value: The p-value gives the fraction of training data with an MD greater than or equal to the one for the given sample, assuming normally distributed data. The smaller the p-value, the less trustworthy the prediciton. For highly non-normal X properties (e.g., fingerprints), the MD p-value is wildly inaccurate.

## Structural Similar Compounds

| Name | Streptozocin | Minocycline | Ribavirin |
|---|---|---|---|
| Structure | 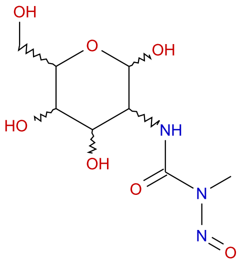 | 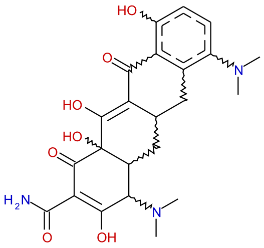 | 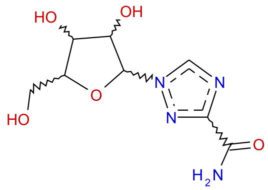 |
| Actual Endpoint | Multiple-Carcinogen | Single-Carcinogen | Multiple-Carcinogen |
| Predicted Endpoint | Multiple-Carcinogen | Single-Carcinogen | Multiple-Carcinogen |
| Distance | 0.817 | 0.908 | 0.929 |
| Reference | US FDA (Centre for Drug Eval.& Res./Off. Testing & Res.) Sept. 1997 | US FDA (Centre for Drug Eval.& Res./Off. Testing & Res.) Sept. 1997 | US FDA (Centre for Drug Eval.& Res./Off. Testing & Res.) Sept. 1997 |

## Model Applicability

Unknown features are fingerprint features in the query molecule, but not found or appearing too infrequently in the training set.

1. Num_H_Acceptors out of range. Value: 11. Training min, max, mean, SD: 0, 9, 3.8906, 2.196.

## Feature Contribution

### Top features for positive contribution

| Fingerprint | Bit/Smiles | Feature Structure | Score | Multiple-Carcinogen in training set |
|---|---|---|---|---|
| SCFP_8 | -1029620989 | 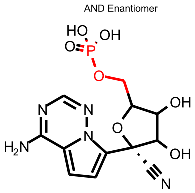 [*]COP(=[*])([*])[*] | 0.649 | 3 out of 3 |

| | | | | |
|---|---|---|---|---|
| SCFP_8 | 2 | 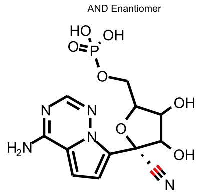 [*]C#[*] | 0.584 | 6 out of 8 |
| SCFP_8 | -1486266146 | 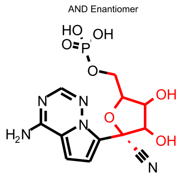 [*]CC1OC([*])([*])C(O )C1O | 0.553 | 2 out of 2 |

## Top Features for negative contribution

| Fingerprint | Bit/Smiles | Feature Structure | Score | Multiple-Carcinogen in training set |
|---|---|---|---|---|
| SCFP_8 | -1381862798 | 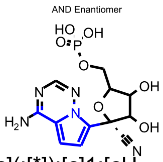 [*][c](:[*]):[c]1:[cH ]:[cH]:[c]([*]):n:1: [*] | -0.572 | 1 out of 7 |
| SCFP_8 | 1245795878 | 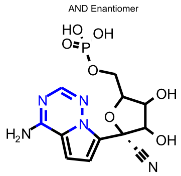 [*][c]1:[*]:n(:[*]):n :[cH]:n:1 | -0.546 | 0 out of 2 |
| SCFP_8 | -1375522316 | 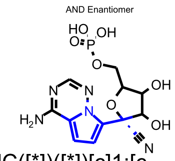 [*]C([*])([*])[c]1:[c H]:[cH]:[c](:[*]):n: 1:[*] | -0.546 | 0 out of 2 |

AND Enantiomer

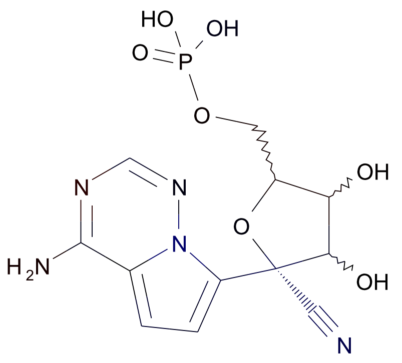

$C_{12}H_{14}N_5O_7P$
Molecular Weight: 371.24262
ALogP: -1.502
Rotatable Bonds: 4
Acceptors: 11
Donors: 5

## Model Prediction

Prediction: Mild

Probability: 0.0911

Enrichment: 0.247

Bayesian Score: -8.73

Mahalanobis Distance: 13.5

Mahalanobis Distance p-value: 1.21e-009

Prediction: Positive if the Bayesian score is above the estimated best cutoff value from minimizing the false positive and false negative rate.
Probability: The esimated probability that the sample is in the positive category. This assumes that the Bayesian score follows a normal distribution and is different from the prediction using a cutoff.
Enrichment: An estimate of enrichment, that is, the increased likelihood (versus random) of this sample being in the category.
Bayesian Score: The standard Laplacian-modified Bayesian score.
Mahalanobis Distance: The Mahalanobis distance (MD) is the distance to the center of the training data. The larger the MD, the less trustworthy the prediction.
Mahalanobis Distance p-value: The p-value gives the fraction of training data with an MD greater than or equal to the one for the given sample, assuming normally distributed data. The smaller the p-value, the less trustworthy the prediciton. For highly non-normal X properties (e.g., fingerprints), the MD p-value is wildly inaccurate.

## Structural Similar Compounds

| Name | 1,3,6-Naphthalenetrisulfonic acid, 7-amino- | 2,7-Anthracenedisulfonic acid, 9,10-dihydro-4,5-diamino-9,10-dioxo- 1-hydroxy-, disodium salt | 1,5-Naphthalenedisulfonic acid, 2-amino- |
|---|---|---|---|
| Structure | 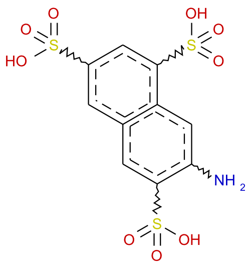 | 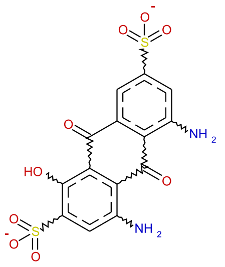 | 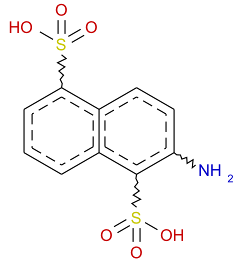 |
| Actual Endpoint | Mild | Mild | Mild |
| Predicted Endpoint | Mild | Mild | Mild |
| Distance | 0.759 | 1.033 | 1.137 |
| Reference | 85JCAE "Prehled Prumyslove Toxikologie; Organicke Latky," Marhold, J., Prague , Czechoslovakia, Avicenum, 1986 Volume(issue)/page/year: -,1058,1986 | 28ZPAK "Sbornik Vysledku Toxixologickeho Vysetreni Latek A Pripravku," Marhold d, J.V., Institut Pro Vychovu Vedoucicn Pracovniku Chemickeho Prumyclu Praha, Cz echoslovakia, 1972 Volume(issue)/page/year: -,239,1 | 85JCAE "Prehled Prumyslove Toxikologie; Organicke Latky," Marhold, J., Prague , Czechoslovakia, Avicenum, 1986 Volume(issue)/page/year: -,1058,1986 |

## Model Applicability

Unknown features are fingerprint features in the query molecule, but not found or appearing too infrequently in the training set.

1. All properties and OPS components are within expected ranges.
2. Unknown FCFP_2 feature: 472180098: [*]OP(=O)(O)O
3. Unknown FCFP_2 feature: -332197802: [*][c]1:[*]:[*]:[c](:[*]):n:1:n:[*]

## Feature Contribution

### Top features for positive contribution

| Fingerprint | Bit/Smiles | Feature Structure | Score | Moderate_Severe in training set |
|---|---|---|---|---|

| | | | | |
|---|---|---|---|---|
| FCFP_12 | -1151884458 | 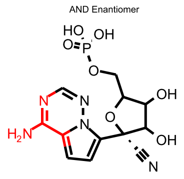<br>[*]:n:[c](N):[c](:[*]<br>):[*] | 0.385 | 1 out of 1 |
| FCFP_12 | 76292238 | 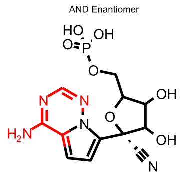<br>[*]:[c]1:[*]:n:[cH]:n<br>:[c]:1N | 0.385 | 1 out of 1 |
| FCFP_12 | -124685461 | 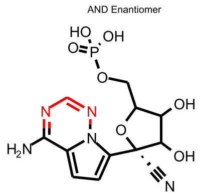<br>[*]:n:[cH]:n:[*] | 0.206 | 2 out of 4 |
| **Top Features for negative contribution** | | | | |
| **Fingerprint** | **Bit/Smiles** | **Feature Structure** | **Score** | **Moderate_Severe in training set** |
| FCFP_12 | 4427049 | 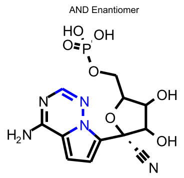<br>[*]:[cH]:n:n(:[*]):[*<br>] | -0.893 | 0 out of 4 |
| FCFP_12 | -1277879912 | 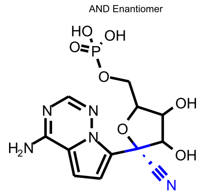<br>[*]C([*])([*])C#N | -0.548 | 5 out of 26 |

| | | | | |
|---|---|---|---|---|
| FCFP_12 | -836603894 | AND Enantiomer 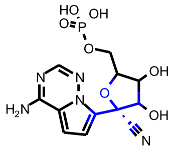 [*]C1[*][*]O[C@]1(C#[*])[c](:[*]):[*] | -0.543 | 0 out of 2 |

# Remdesivir

# TOPKAT_Skin_Irritancy_None_vs_Irritant

AND Enantiomer

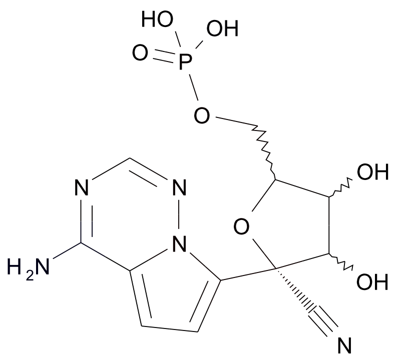

$C_{12}H_{14}N_5O_7P$

Molecular Weight: 371.24262

ALogP: -1.502

Rotatable Bonds: 4

Acceptors: 11

Donors: 5

## Model Prediction

Prediction: Irritant

Probability: 0.976

Enrichment: 1.06

Bayesian Score: -0.492

Mahalanobis Distance: 13.2

Mahalanobis Distance p-value: 3.18e-008

Prediction: Positive if the Bayesian score is above the estimated best cutoff value from minimizing the false positive and false negative rate.
Probability: The esimated probability that the sample is in the positive category. This assumes that the Bayesian score follows a normal distribution and is different from the prediction using a cutoff.
Enrichment: An estimate of enrichment, that is, the increased likelihood (versus random) of this sample being in the category.
Bayesian Score: The standard Laplacian-modified Bayesian score.
Mahalanobis Distance: The Mahalanobis distance (MD) is the distance to the center of the training data. The larger the MD, the less trustworthy the prediction.
Mahalanobis Distance p-value: The p-value gives the fraction of training data with an MD greater than or equal to the one for the given sample, assuming normally distributed data. The smaller the p-value, the less trustworthy the prediciton. For highly non-normal X properties (e.g., fingerprints), the MD p-value is wildly inaccurate.

## Structural Similar Compounds

| Name | 1,3,6-Naphthalenetrisulfonic acid, 7-amino- | 2,2'-Benzidine disulfonic acid | 2,7-Anthracenedisulfonic acid, 9,10-dihydro-4,5-diamino-9,10-dioxo- 1-hydroxy-, disodium salt |
|---|---|---|---|
| Structure | 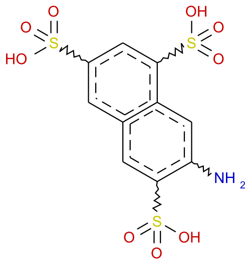 | 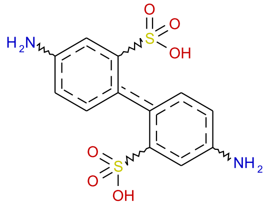 | 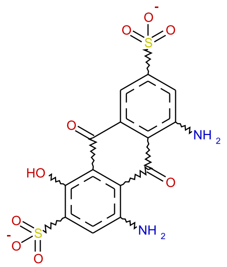 |
| Actual Endpoint | Irritant | Non-Irritant | Irritant |
| Predicted Endpoint | Non-Irritant | Non-Irritant | Non-Irritant |
| Distance | 0.755 | 0.896 | 1.025 |
| Reference | 85JCAE "Prehled Prumyslove Toxikologie; Organicke Latky," Marhold, J., Prague , Czechoslovakia, Avicenum, 1986 Volume(issue)/page/year: -,1058,1986 | 28ZPAK -,191,72 | 28ZPAK "Sbornik Vysledku Toxixologickeho Vysetreni Latek A Pripravku," Marhol d, J.V., Institut Pro Vychovu Vedoucicn Pracovniku Chemickeho Prumyclu Praha, Cz echoslovakia, 1972 Volume(issue)/page/year: -,239,1 |

## Model Applicability

Unknown features are fingerprint features in the query molecule, but not found or appearing too infrequently in the training set.

1. All properties and OPS components are within expected ranges.
2. Unknown FCFP_2 feature: 472180098: [*]OP(=O)(O)O
3. Unknown FCFP_2 feature: -332197802: [*][c]1:[*]:[*]:[c](:[*]):n:1:n:[*]

## Feature Contribution

| Top features for positive contribution | | | | |
|---|---|---|---|---|
| **Fingerprint** | **Bit/Smiles** | **Feature Structure** | **Score** | **Irritant in training set** |

| | | | | |
|---|---|---|---|---|
| FCFP_12 | 654335567 | 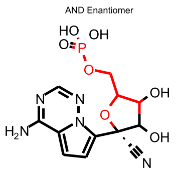 [*]C1[*][*]OC1COP(=[*])([*])[*] | 0.0856 | 29 out of 29 |
| FCFP_12 | -1539132615 | 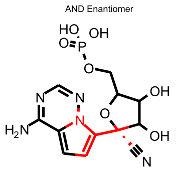 [*]C([*])([*])[c]1:[cH]:[*]:[*]:n:1:[*] | 0.0795 | 9 out of 9 |
| FCFP_12 | -1280036918 | 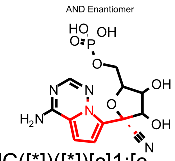 [*]C([*])([*])[c]1:[cH]:[cH]:[c](:[*]):n:1:[*] | 0.0772 | 7 out of 7 |

**Top Features for negative contribution**

| Fingerprint | Bit/Smiles | Feature Structure | Score | Irritant in training set |
|---|---|---|---|---|
| FCFP_12 | 1069584379 | 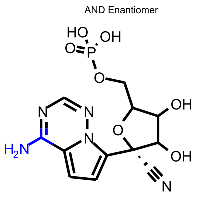 [*]:[c](:[*])N | -0.439 | 38 out of 65 |
| FCFP_12 | 1618154665 | 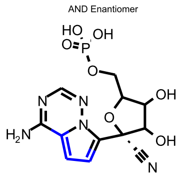 [*]:[c]1:[*]:[*]:[cH]:[cH]:1 | -0.0845 | 412 out of 490 |

| | | | | |
|---|---|---|---|---|
| FCFP_12 | 16 | 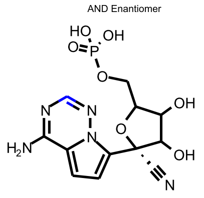 [*]:[cH]:[*] | -0.0843 | 423 out of 503 |

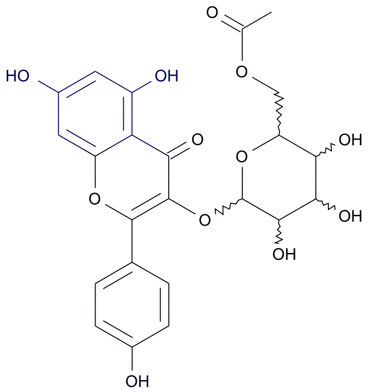

$C_{23}H_{22}O_{12}$
Molecular Weight: 490.41357
ALogP: 0.322
Rotatable Bonds: 6
Acceptors: 12
Donors: 6

## Model Prediction

Prediction: Non-Irritant
Probability: 0.892
Enrichment: 0.969
Bayesian Score: -2.84
Mahalanobis Distance: 13.3
Mahalanobis Distance p-value: 8.75e-009

Prediction: Positive if the Bayesian score is above the estimated best cutoff value from minimizing the false positive and false negative rate.
Probability: The esimated probability that the sample is in the positive category. This assumes that the Bayesian score follows a normal distribution and is different from the prediction using a cutoff.
Enrichment: An estimate of enrichment, that is, the increased likelihood (versus random) of this sample being in the category.
Bayesian Score: The standard Laplacian-modified Bayesian score.
Mahalanobis Distance: The Mahalanobis distance (MD) is the distance to the center of the training data. The larger the MD, the less trustworthy the prediction.
Mahalanobis Distance p-value: The p-value gives the fraction of training data with an MD greater than or equal to the one for the given sample, assuming normally distributed data. The smaller the p-value, the less trustworthy the prediciton. For highly non-normal X properties (e.g., fingerprints), the MD p-value is wildly inaccurate.

## Structural Similar Compounds

| Name | 1,3,6-Naphthalenetrisulfonic acid, 7-amino- | 2,2'-Benzidine disulfonic acid | Amipurimycin, hydrate |
|---|---|---|---|
| Structure | 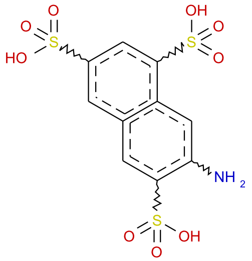 | 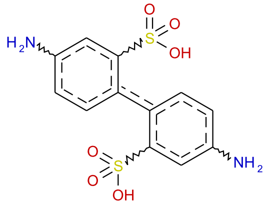 | 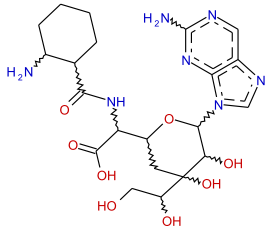 |
| Actual Endpoint | Irritant | Non-Irritant | Irritant |
| Predicted Endpoint | Non-Irritant | Non-Irritant | Non-Irritant |
| Distance | 1.105 | 1.157 | 1.230 |
| Reference | 85JCAE "Prehled Prumyslove Toxikologie; Organicke Latky," Marhold, J., Prague , Czechoslovakia, Avicenum, 1986 Volume(issue)/page/year: -,1058,1986 | 28ZPAK -,191,72 | JANTAJ Journal of Antibiotics. (Japan Antibiotics Research Assoc., 2-20-8 Ka miosaki, Shinagawa-ku, Tokyo, 141, Japan) V.2-5, 1948-52; V.21- 1968- Volume(issue)/page/year: 30,1,1977 |

## Model Applicability

Unknown features are fingerprint features in the query molecule, but not found or appearing too infrequently in the training set.

1. All properties and OPS components are within expected ranges.

## Feature Contribution

**Top features for positive contribution**

| Fingerprint | Bit/Smiles | Feature Structure | Score | Irritant in training set |
|---|---|---|---|---|

| | | | | |
|---|---|---|---|---|
| FCFP_12 | 654335567 | 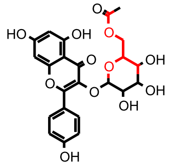<br>[*]C1[*][*]OC1COP(=[* ])([*])[*] | 0.0856 | 29 out of 29 |
| FCFP_12 | 699500266 | 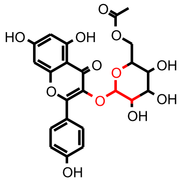<br>[*]OC(O[*])C([*])[*] | 0.0852 | 25 out of 25 |
| FCFP_12 | 436915834 | 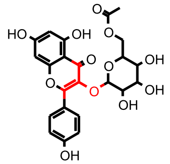<br>[*]OC(=C([*])[*])C(=[ *])[*] | 0.0756 | 6 out of 6 |

## Top Features for negative contribution

| Fingerprint | Bit/Smiles | Feature Structure | Score | Irritant in training set |
|---|---|---|---|---|
| FCFP_12 | 946068634 | 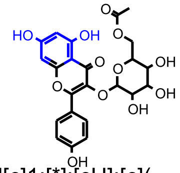<br>[*][c]1:[*]:[cH]:[c]( O):[cH]:[c]:1O | -1.04 | 0 out of 2 |
| FCFP_12 | 115228054 | 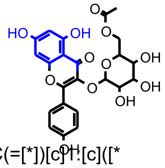<br>[*]C(=[*])[c]1:[c]([* ]):[cH]:[c](O):[cH]: [c]:1O | -0.65 | 0 out of 1 |

| | | | | |
|---|---|---|---|---|
| FCFP_12 | -1604301295 | 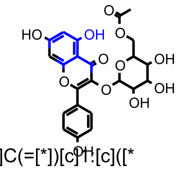<br>[*]C(=[*])[c]1:[c]([*]):[*]:[c]([*]):[cH]:[c]:1O | -0.18 | 22 out of 29 |

# Remdesivir

# TOPKAT_Carcinogenic_Potency_TD50_Mouse

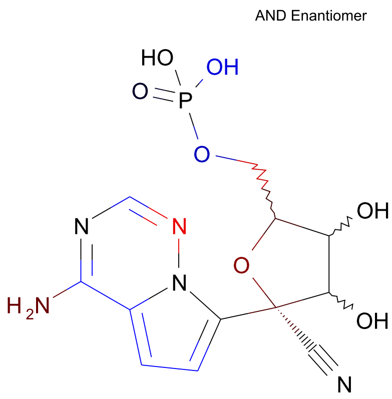


$C_{12}H_{14}N_5O_7P$

Molecular Weight: 371.24262

ALogP: -1.502

Rotatable Bonds: 4

Acceptors: 11

Donors: 5

## Structural Similar Compounds

| Name | 377 | (N-6)-(Methylnitroso)adenosine | 338 |
|---|---|---|---|
| Structure | 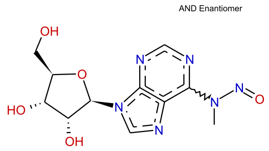 | 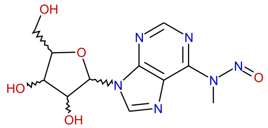 | 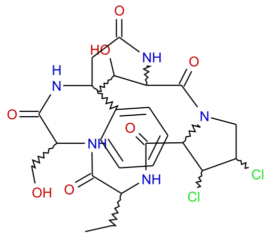 |
| Actual Endpoint (-log C) | 4.22928 | 4.22928 | 4.39533 |
| Predicted Endpoint (-log C) | 5.36013 | 5.36013 | 4.31268 |
| Distance | 0.852 | 0.852 | 0.919 |
| Reference | CPDB | CPDB | CPDB |

## Model Prediction

Prediction: 9.25

Unit: mg/kg_body_weight/day

Mahalanobis Distance: 14

Mahalanobis Distance p-value: 2.59e-010

Mahalanobis Distance: The Mahalanobis distance (MD) is a generalization of the Euclidean distance that accounts for correlations among the X properties. It is calculated as the distance to the center of the training data. The larger the MD, the less trustworthy the prediction.
Mahalanobis Distance p-value: The p-value gives the fraction of training data with an MD greater than or equal to the one for the given sample, assuming normally distributed data. The smaller the p-value, the less trustworthy the prediciton. For highly non-normal X properties (e.g., fingerprints), the MD p-value is wildly inaccurate.

## Model Applicability

Unknown features are fingerprint features in the query molecule, but not found or appearing too infrequently in the training set.

1. All properties and OPS components are within expected ranges.
2. Unknown ECFP_2 feature: 1126642748: [*]OP(=O)(O)O
3. Unknown ECFP_2 feature: 2024329577: [*]P(=[*])([*])O
4. Unknown ECFP_2 feature: -194719409: [*]C1[*][*]C([*])([*])O1
5. Unknown ECFP_2 feature: 1258791451: [*]C1[*][*]O[C@]1(C#[*])[c](:[*]):[*]
6. Unknown ECFP_2 feature: -264833661: [*]C([*])([*])C#N
7. Unknown ECFP_2 feature: -1507082173: [*][c]1:[*]:[*]:[c](:[*]):n:1:n:[*]
8. Unknown ECFP_2 feature: -676555381: [*]:[cH]:n:n(:[*]):[*]
9. Unknown ECFP_2 feature: -66263742: [*]C([*])([*])[c]1:[cH]:[*]:[*]:n:1:[*]

## Feature Contribution

| Top features for positive contribution | | | |
|---|---|---|---|
| Fingerprint | Bit/Smiles | Feature Structure | Score |

| | | | |
|---|---|---|---|
| ECFP_6 | 655739385 | 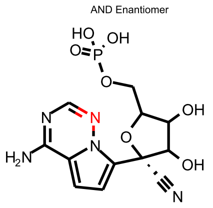  [*]:n:[*] | 0.229 |
| ECFP_6 | 1572579716 | 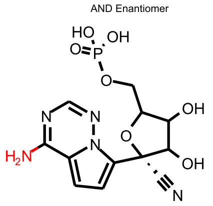  [*]N | 0.225 |
| ECFP_6 | 1559650422 | 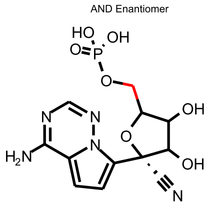  [*]C[*] | 0.203 |

**Top Features for negative contribution**

| Fingerprint | Bit/Smiles | Feature Structure | Score |
|---|---|---|---|
| ECFP_6 | 1996767644 | 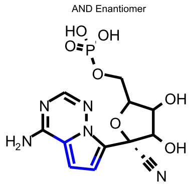  [*]:[c]1:[*]:[*]:[cH]:[cH]:1 | -0.251 |
| ECFP_6 | 642810091 | 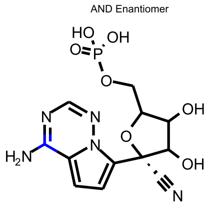  [*][c](:[*]):[*] | -0.247 |

| | | | |
|---|---|---|---|
| ECFP_6 | -182236392 | 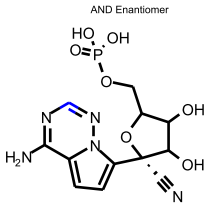 [*]:[cH]:[*] | -0.232 |

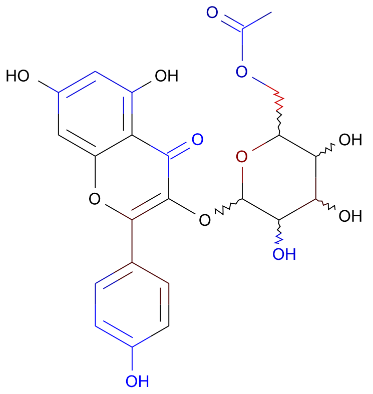

$C_{23}H_{22}O_{12}$
Molecular Weight: 490.41357
ALogP: 0.322
Rotatable Bonds: 6
Acceptors: 12
Donors: 6

## Model Prediction

Prediction: 44.3
Unit: mg/kg_body_weight/day
Mahalanobis Distance: 13.3
Mahalanobis Distance p-value: 1.71e-008

Mahalanobis Distance: The Mahalanobis distance (MD) is a generalization of the Euclidean distance that accounts for correlations among the X properties. It is calculated as the distance to the center of the training data. The larger the MD, the less trustworthy the prediction.
Mahalanobis Distance p-value: The p-value gives the fraction of training data with an MD greater than or equal to the one for the given sample, assuming normally distributed data. The smaller the p-value, the less trustworthy the prediciton. For highly non-normal X properties (e.g., fingerprints), the MD p-value is wildly inaccurate.

## Structural Similar Compounds

| Name | 338 | 532 | Cyclochlorotine |
|---|---|---|---|
| Structure | 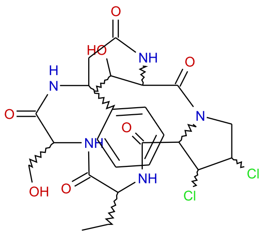 | 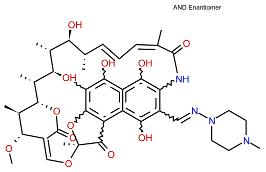 | 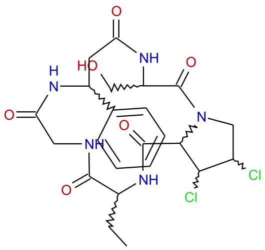 |
| Actual Endpoint (-log C) | 4.39533 | 4.38903 | 4.36142 |
| Predicted Endpoint (-log C) | 4.31268 | 5.60554 | 4.0834 |
| Distance | 0.898 | 0.966 | 0.994 |
| Reference | CPDB | CPDB | CPDB |

## Model Applicability

Unknown features are fingerprint features in the query molecule, but not found or appearing too infrequently in the training set.

1. All properties and OPS components are within expected ranges.
2. Unknown ECFP_2 feature: -2060414325: [*]OC(O[*])C([*])[*]
3. Unknown ECFP_2 feature: 1310940530: [*]C([*])OC(=[*])[*]
4. Unknown ECFP_2 feature: 1795218697: [*]OC(=C([*])[*])C(=[*])[*]
5. Unknown ECFP_2 feature: 1796421070: [*]OC(=C([*])[*])[c](:[*]):[*]

## Feature Contribution

| Top features for positive contribution | | | |
|---|---|---|---|
| Fingerprint | Bit/Smiles | Feature Structure | Score |
| ECFP_6 | 1559650422 | 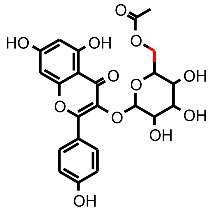 [*]C[*] | 0.203 |

| | | | |
|---|---|---|---|
| ECFP_6 | 683445015 | 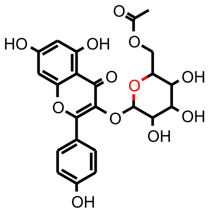 [*]O[*] | 0.136 |
| ECFP_6 | -181568884 | 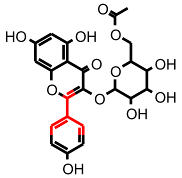 [*]C(=[*])[c](:[cH]:[ *]):[cH]:[*] | 0.0725 |

**Top Features for negative contribution**

| Fingerprint | Bit/Smiles | Feature Structure | Score |
|---|---|---|---|
| ECFP_6 | 2106656448 | 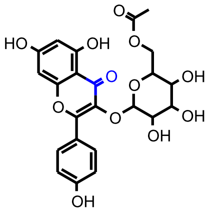 [*]C(=O)[*] | -0.275 |
| ECFP_6 | 2019062761 | 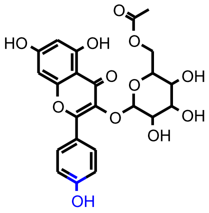 [*]:[c](:[*])O | -0.258 |
| ECFP_6 | 1996767644 | 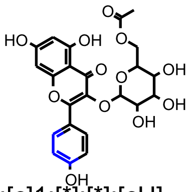 [*]:[c]1:[*]:[*]:[cH]:[cH]:1 | -0.251 |

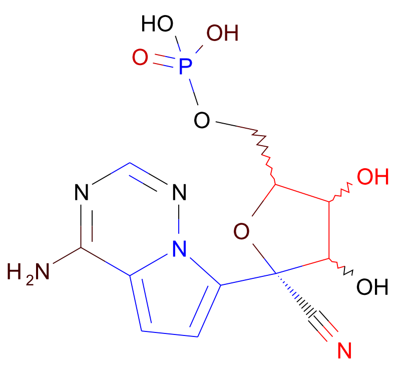


$C_{12}H_{14}N_5O_7P$

Molecular Weight: 371.24262

ALogP: -1.502

Rotatable Bonds: 4

Acceptors: 11

Donors: 5

## Model Prediction

Prediction: 1.01

Unit: mg/kg_body_weight/day

Mahalanobis Distance: 16.2

Mahalanobis Distance p-value: 4.38e-015

Mahalanobis Distance: The Mahalanobis distance (MD) is a generalization of the Euclidean distance that accounts for correlations among the X properties. It is calculated as the distance to the center of the training data. The larger the MD, the less trustworthy the prediction.
Mahalanobis Distance p-value: The p-value gives the fraction of training data with an MD greater than or equal to the one for the given sample, assuming normally distributed data. The smaller the p-value, the less trustworthy the prediciton. For highly non-normal X properties (e.g., fingerprints), the MD p-value is wildly inaccurate.

## Structural Similar Compounds

| Name | b-Thioguanine deoxyriboside | Hexamethylmelamine | 604 |
|---|---|---|---|
| Structure | 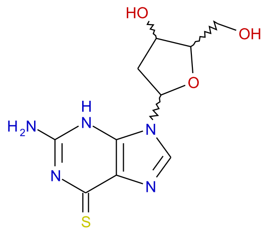 | 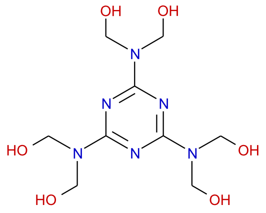 | 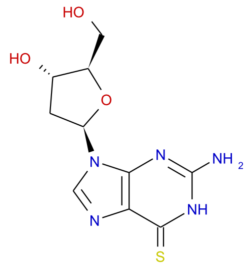 |
| Actual Endpoint (-log C) | 5.13004 | 4.47751 | 5.13004 |
| Predicted Endpoint (-log C) | 4.82552 | 3.76275 | 4.96687 |
| Distance | 0.805 | 0.832 | 0.835 |
| Reference | CPDB | CPDB | CPDB |

## Model Applicability

Unknown features are fingerprint features in the query molecule, but not found or appearing too infrequently in the training set.

1. All properties and OPS components are within expected ranges.
2. Unknown FCFP_2 feature: 472180098: [*]OP(=O)(O)O
3. Unknown FCFP_2 feature: -836603894: [*]C1[*][*]O[C@]1(C#[*])[c](:[*]):[*]

## Feature Contribution

**Top features for positive contribution**

| Fingerprint | Bit/Smiles | Feature Structure | Score |
|---|---|---|---|
| FCFP_6 | -1043250487 | 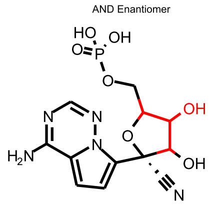 [*]C1[*][*]C([*])C1O | 1.15 |

| | | | |
|---|---|---|---|
| FCFP_6 | 9 | 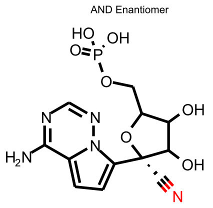 [*]#N | 0.385 |
| FCFP_6 | 1 | 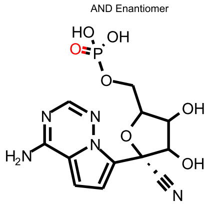 [*]=O | 0.234 |

## Top Features for negative contribution

| **Fingerprint** | **Bit/Smiles** | **Feature Structure** | **Score** |
|---|---|---|---|
| FCFP_6 | -1280036918 | 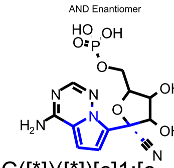 [*]C([*])([*])[c]1:[cH]:[cH]:[c](:[*]):n:1:[*] | -0.363 |
| FCFP_6 | 16 | 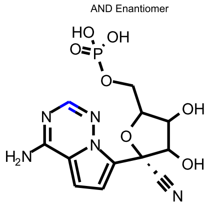 [*]:[cH]:[*] | -0.354 |
| FCFP_6 | 17 | 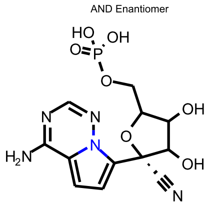 [*]:n(:[*]):[*] | -0.149 |

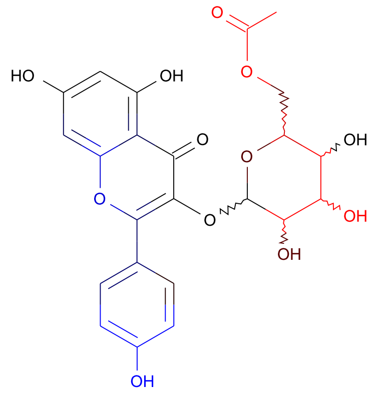


$C_{23}H_{22}O_{12}$

Molecular Weight: 490.41357

ALogP: 0.322

Rotatable Bonds: 6

Acceptors: 12

Donors: 6

## Model Prediction

Prediction: 0.544

Unit: mg/kg_body_weight/day

Mahalanobis Distance: 12.1

Mahalanobis Distance p-value: 0.000325

Mahalanobis Distance: The Mahalanobis distance (MD) is a generalization of the Euclidean distance that accounts for correlations among the X properties. It is calculated as the distance to the center of the training data. The larger the MD, the less trustworthy the prediction.
Mahalanobis Distance p-value: The p-value gives the fraction of training data with an MD greater than or equal to the one for the given sample, assuming normally distributed data. The smaller the p-value, the less trustworthy the prediciton. For highly non-normal X properties (e.g., fingerprints), the MD p-value is wildly inaccurate.

## Structural Similar Compounds

| Name | Quercetin | Hexamethylmelamine | Salicylazosulfapyridine |
|---|---|---|---|
| Structure | 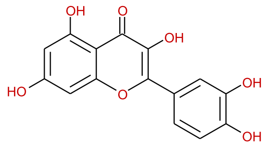 | 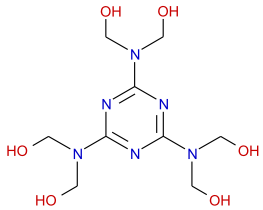 | 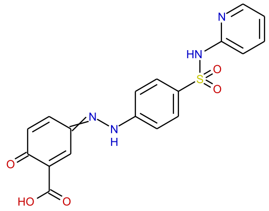 |
| Actual Endpoint (-log C) | 4.47602 | 4.47751 | 2.39891 |
| Predicted Endpoint (-log C) | 3.79194 | 3.76275 | 3.17598 |
| Distance | 0.891 | 0.945 | 0.974 |
| Reference | CPDB | CPDB | CPDB |

## Model Applicability

Unknown features are fingerprint features in the query molecule, but not found or appearing too infrequently in the training set.

1. All properties and OPS components are within expected ranges.

## Feature Contribution

**Top features for positive contribution**

| Fingerprint | Bit/Smiles | Feature Structure | Score |
|---|---|---|---|
| FCFP_6 | -1043250487 | 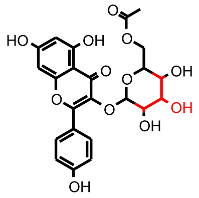 [*]C1[*][*]C([*])C1O | 1.15 |

| | | | |
|---|---|---|---|
| FCFP_6 | 565998553 | 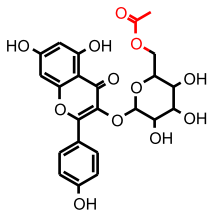 [*]OC(=O)C | 0.357 |
| FCFP_6 | 1 | 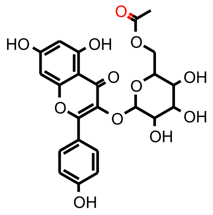 [*]=O | 0.234 |
| **Top Features for negative contribution** | | | |
| **Fingerprint** | **Bit/Smiles** | **Feature Structure** | **Score** |
| FCFP_6 | 7 | 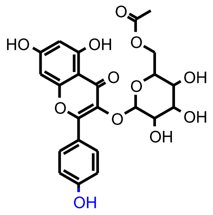 [*]O | -0.372 |
| FCFP_6 | 16 | 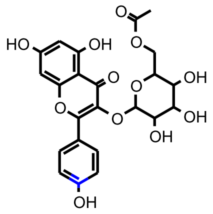 [*]:[cH]:[*] | -0.354 |
| FCFP_6 | 74595001 | 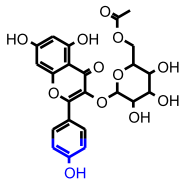 [*]:[cH]:[c](O):[cH]:[*] | -0.267 |

# Remdesivir

# TOPKAT_Chronic_LOAEL

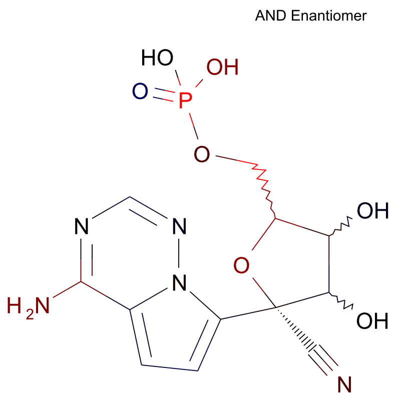


$C_{12}H_{14}N_5O_7P$

Molecular Weight: 371.24262

ALogP: -1.502

Rotatable Bonds: 4

Acceptors: 11

Donors: 5

## Structural Similar Compounds

| Name | TETRACYCLINE .HCL | 4;4'-DIAMINO-2;2'-STILBENEDIS | OXYTETRACYCLINE .HCL |
|---|---|---|---|
| Structure | 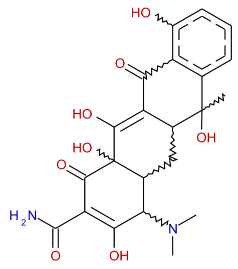 | 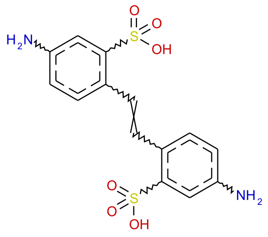 | 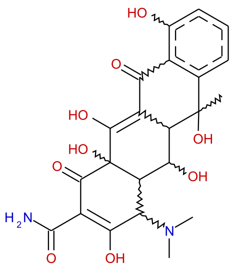 |
| Actual Endpoint (-log C) | 2.85193 | 2.47175 | 2.56626 |
| Predicted Endpoint (-log C) | 3.94748 | 3.53715 | 3.75581 |
| Distance | 0.746 | 0.746 | 0.802 |
| Reference | NTP REPORT # 344 | NTP 412 82 | NTP REPORT # 315 |

## Model Prediction

Prediction: 0.00379

Unit: g/kg_body_weight

Mahalanobis Distance: 47.7

Mahalanobis Distance p-value: 2.93e-054

Mahalanobis Distance: The Mahalanobis distance (MD) is a generalization of the Euclidean distance that accounts for correlations among the X properties. It is calculated as the distance to the center of the training data. The larger the MD, the less trustworthy the prediction.
Mahalanobis Distance p-value:  The p-value gives the fraction of training data with an MD greater than or equal to the one for the given sample, assuming normally distributed data. The smaller the p-value, the less trustworthy the prediciton. For highly non-normal X properties (e.g., fingerprints), the MD p-value is wildly inaccurate.

## Model Applicability

Unknown features are fingerprint features in the query molecule, but not found or appearing too infrequently in the training set.

1. All properties and OPS components are within expected ranges.
2. Unknown FCFP_2 feature: 472180098: [*]OP(=O)(O)O
3. Unknown FCFP_2 feature: -332197802: [*][c]1:[*]:[*]:[c](:[*]):n:1:n:[*]
4. Unknown ECFP_6 feature: -1114776580: [*]C#[*]
5. Unknown ECFP_6 feature: -1101847286: [*]#N
6. Unknown ECFP_6 feature: 672362763: [*]:n(:[*]):[*]
7. Unknown ECFP_6 feature: 1126642748: [*]OP(=O)(O)O
8. Unknown ECFP_6 feature: 2100964382: [*]P(=O)([*])[*]
9. Unknown ECFP_6 feature: 2024329577: [*]P(=[*])([*])O
10. Unknown ECFP_6 feature: -1250439909: [*]COP(=[*])([*])[*]
11. Unknown ECFP_6 feature: -1687549011: [*]OCC([*])[*]
12. Unknown ECFP_6 feature: -194719409: [*]C1[*][*]C([*])([*])O1
13. Unknown ECFP_6 feature: -553149446: [*]CC1O[*][*]C1[*]
14. Unknown ECFP_6 feature: 305695353: [*]C1[*][*]C([*])C1O
15. Unknown ECFP_6 feature: -521596699: [*]C1[*][*]C([*])([*])C1O
16. Unknown ECFP_6 feature: 1258791451: [*]C1[*][*]O[C@]1(C#[*])[c](:[*]):[*]
17. Unknown ECFP_6 feature: 2024749573: [*]C([*])O
18. Unknown ECFP_6 feature: -264833661: [*]C([*])([*])C#N
19. Unknown ECFP_6 feature: 1412053881: [*]C#N

20. Unknown ECFP_6 feature: -1507082173: [*][c]1:[*]:[*]:[c](:[*]):n:1:n:[*]
21. Unknown ECFP_6 feature: -676555381: [*]:[cH]:n:n(:[*]):[*]
22. Unknown ECFP_6 feature: -710237522: [*]:n:[cH]:n:[*]
23. Unknown ECFP_6 feature: -677309799: [*][c](:[*]):n:[cH]:[*]
24. Unknown ECFP_6 feature: -1734834311: [*]:n:[c](N):[c](:[*]):[*]
25. Unknown ECFP_6 feature: 1334415134: [*][c](:[*]):[c]1:[cH]:[*]:[*]:n:1:[*]
26. Unknown ECFP_6 feature: -66263742: [*]C([*])([*])[c]1:[cH]:[*]:[*]:n:1:[*]
27. Unknown ECFP_6 feature: -938530932: [*]:[c](:[*])N

## Feature Contribution

### Top features for positive contribution

| Fingerprint | Bit/Smiles | Feature Structure | Score |
|---|---|---|---|
| ECFP_6 | -167460056 | 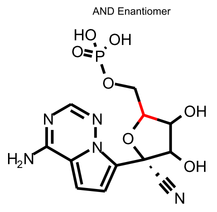 [*]C([*])[*] | 0.136 |
| FCFP_6 | -1143715940 | 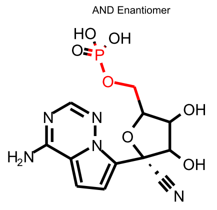 [*]COP(=[*])([*])[*] | 0.13 |
| ECFP_6 | 1559650422 | 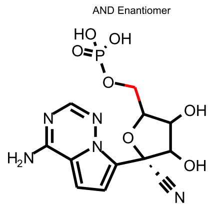 [*]C[*] | 0.129 |

### Top Features for negative contribution

| Fingerprint | Bit/Smiles | Feature Structure | Score |
|---|---|---|---|

| | | | |
|---|---|---|---|
| FCFP_6 | 1 | 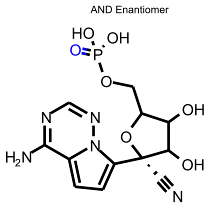 [*]=O | -0.102 |
| ECFP_6 | 1996767644 | 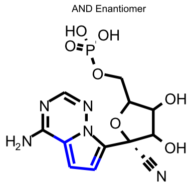 [*]:[c]1:[*]:[*]:[cH]:[cH]:1 | -0.0497 |
| FCFP_6 | 16 | 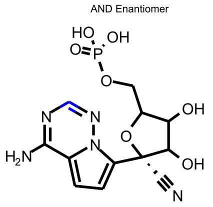 [*]:[cH]:[*] | -0.0462 |

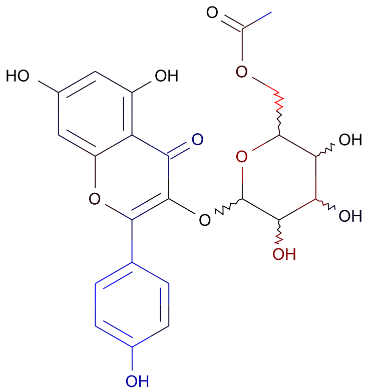


$C_{23}H_{22}O_{12}$

Molecular Weight: 490.41357

ALogP: 0.322

Rotatable Bonds: 6

Acceptors: 12

Donors: 6

## Model Prediction

Prediction: 0.0802

Unit: g/kg_body_weight

Mahalanobis Distance: 29.6

Mahalanobis Distance p-value: 5.79e-024

Mahalanobis Distance: The Mahalanobis distance (MD) is a generalization of the Euclidean distance that accounts for correlations among the X properties. It is calculated as the distance to the center of the training data. The larger the MD, the less trustworthy the prediction.
Mahalanobis Distance p-value: The p-value gives the fraction of training data with an MD greater than or equal to the one for the given sample, assuming normally distributed data. The smaller the p-value, the less trustworthy the prediciton. For highly non-normal X properties (e.g., fingerprints), the MD p-value is wildly inaccurate.

## Structural Similar Compounds

| Name | TETRACYCLINE .HCL | OXYTETRACYCLINE .HCL | CARMINE |
|---|---|---|---|
| Structure | 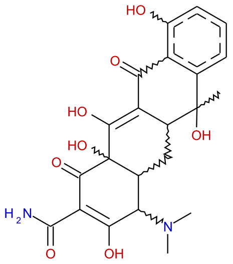 | 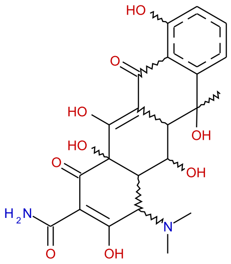 | 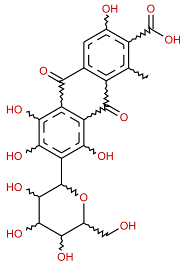 |
| Actual Endpoint (-log C) | 2.85193 | 2.56626 | 3.5162 |
| Predicted Endpoint (-log C) | 3.94748 | 3.75581 | 3.66829 |
| Distance | 0.748 | 0.781 | 0.823 |
| Reference | NTP REPORT # 344 | NTP REPORT # 315 | FOOD.CHEM.TOXICOL.25.897.1987 |

## Model Applicability

Unknown features are fingerprint features in the query molecule, but not found or appearing too infrequently in the training set.

1. All properties and OPS components are within expected ranges.
2. Unknown ECFP_6 feature: -473922720: [*]OC(=O)C
3. Unknown ECFP_6 feature: -1687549011: [*]OCC([*])[*]
4. Unknown ECFP_6 feature: 2024749573: [*]C([*])O
5. Unknown ECFP_6 feature: 305695353: [*]C1[*][*]C([*])C1O
6. Unknown ECFP_6 feature: -2060414325: [*]OC(O[*])C([*])[*]
7. Unknown ECFP_6 feature: -1409796893: [*]C([*])OC([*])[*]
8. Unknown ECFP_6 feature: -553149446: [*]CC1O[*][*]C1[*]
9. Unknown ECFP_6 feature: 2019062761: [*]:[c](:[*])O
10. Unknown ECFP_6 feature: 1310940530: [*]C([*])OC(=[*])[*]
11. Unknown ECFP_6 feature: -177786161: [*]:[cH]:[c](O):[cH]:[*]
12. Unknown ECFP_6 feature: -181568884: [*]C(=[*])[c](:[cH]:[*]):[cH]:[*]
13. Unknown ECFP_6 feature: 1795218697: [*]OC(=C([*])[*])C(=[*])[*]
14. Unknown ECFP_6 feature: 1717462980: [*]C(=[*])C(=O)[c](:[*]):[*]
15. Unknown ECFP_6 feature: 1796421070: [*]OC(=C([*])[*])[c](:[*]):[*]
16. Unknown ECFP_6 feature: -560785749: [*]C(=[*])O[c](:[*]):[*]
17. Unknown ECFP_6 feature: -813997308: [*]C(=[*])[c](:[c]([*]):[*]):[c]([*]):[*]
18. Unknown ECFP_6 feature: -570915357: [*]O[c](:[cH]:[*]):[c]([*]):[*]

## Feature Contribution

| Fingerprint | Bit/Smiles | Feature Structure | Score |
|---|---|---|---|
| **Top features for positive contribution** | | | |
| **Fingerprint** | **Bit/Smiles** | **Feature Structure** | **Score** |
| ECFP_6 | -167460056 | 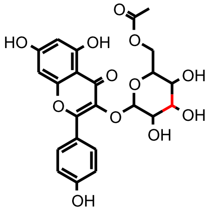 [*]C([*])[*] | 0.136 |
| FCFP_6 | -1143715940 | 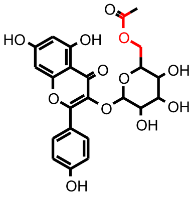 [*]COP(=[*])([*])[*] | 0.13 |
| ECFP_6 | 1559650422 | 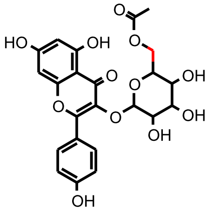 [*]C[*] | 0.129 |
| **Top Features for negative contribution** | | | |
| **Fingerprint** | **Bit/Smiles** | **Feature Structure** | **Score** |
| ECFP_6 | 2106656448 | 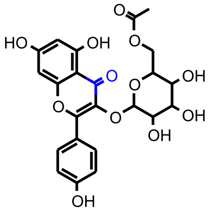 [*]C(=O)[*] | -0.11 |

| | | | |
|---|---|---|---|
| FCFP_6 | 1 | 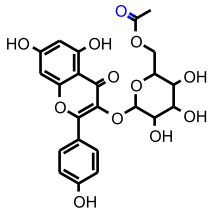<br>[*]=O | -0.102 |
| FCFP_6 | -453677277 | 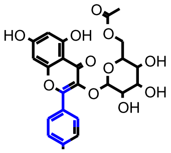<br>[*]C(=[*])[c]1:[cH]:[*]:[c]([*]):[cH]:[cH]:1 | -0.0906 |

# Remdesivir

# TOPKAT_Rat_Maximum_Tolerated_Dose_Feed

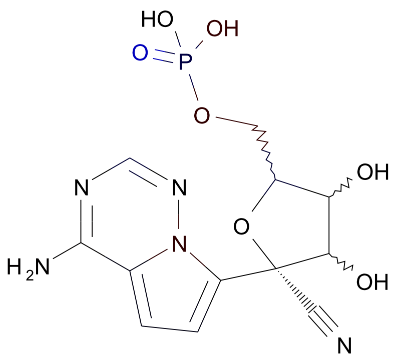


$C_{12}H_{14}N_5O_7P$

Molecular Weight: 371.24262

ALogP: -1.502

Rotatable Bonds: 4

Acceptors: 11

Donors: 5

## Structural Similar Compounds

| Name | 4,4'-DIAMINO-2,2'-STILBENEDISULFONIC ACID.2NaSALT | OXYTETRACYCLINE | 50%1,4,5,8-TETRAAMINOANTHRAQUIONONE + DERIVATIVES |
|---|---|---|---|
| Structure | 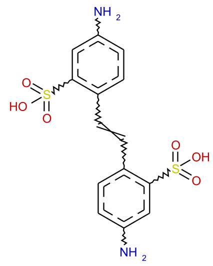 | 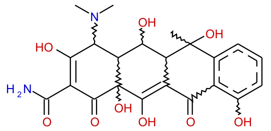 | 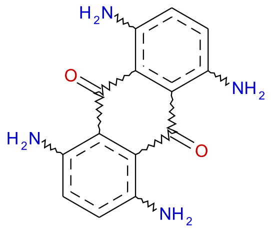 |
| Actual Endpoint (-log C) | 2.50759 | 2.36214 | 3.0764 |
| Predicted Endpoint (-log C) | 3.26068 | 2.77834 | 3.08142 |
| Distance | 0.743 | 0.818 | 0.989 |
| Reference | NCI/NTP TR-412 | NCI/NTP TR-315 | NCI/NTP TR-299 |

## Model Prediction

Prediction: 0.235

Unit: g/kg_body_weight

Mahalanobis Distance: 9.52

Mahalanobis Distance p-value: 0.000247

Mahalanobis Distance: The Mahalanobis distance (MD) is a generalization of the Euclidean distance that accounts for correlations among the X properties. It is calculated as the distance to the center of the training data. The larger the MD, the less trustworthy the prediction.

Mahalanobis Distance p-value: The p-value gives the fraction of training data with an MD greater than or equal to the one for the given sample, assuming normally distributed data. The smaller the p-value, the less trustworthy the prediciton. For highly non-normal X properties (e.g., fingerprints), the MD p-value is wildly inaccurate.

## Model Applicability

Unknown features are fingerprint features in the query molecule, but not found or appearing too infrequently in the training set.

1. Molecular_PolarSurfaceArea out of range. Value: 206.26. Training min, max, mean, SD: 0, 201.84, 63.052, 40.7.
2. Unknown FCFP_2 feature: 472180098: [*]OP(=O)(O)O
3. Unknown FCFP_2 feature: -836603894: [*]C1[*][*]O[C@]1(C#[*])[c](:[*]):[*]
4. Unknown FCFP_2 feature: -1277879912: [*]C([*])([*])C#N
5. Unknown FCFP_2 feature: -332197802: [*][c]1:[*]:[*]:[c](:[*]):n:1:n:[*]
6. Unknown FCFP_2 feature: -124685461: [*]:n:[cH]:n:[*]

## Feature Contribution

### Top features for positive contribution

| Fingerprint | Bit/Smiles | Feature Structure | Score |
|---|---|---|---|

| | | | |
|---|---|---|---|
| FCFP_2 | -1143715940 | 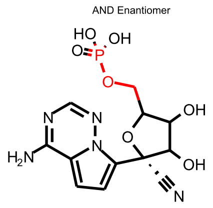 [*]COP(=[*])([*])[*] | 0.095 |
| FCFP_2 | 3 | 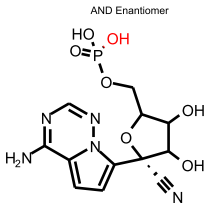 [*]O | 0.0737 |
| FCFP_2 | 17 | 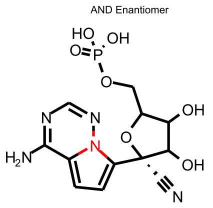 [*]:n(:[*]):[*] | 0.0441 |
| **Top Features for negative contribution** | | | |
| **Fingerprint** | **Bit/Smiles** | **Feature Structure** | **Score** |
| FCFP_2 | 1872154524 | 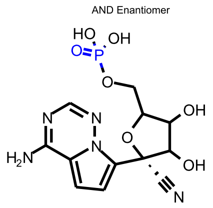 [*]P(=O)([*])[*] | -0.105 |
| FCFP_2 | 1 | 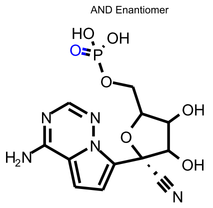 [*]=O | -0.0796 |

| | | | |
|---|---|---|---|
| FCFP_2 | 16 | AND Enantiomer 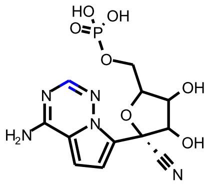 [*]:[cH]:[*] | -0.0512 |

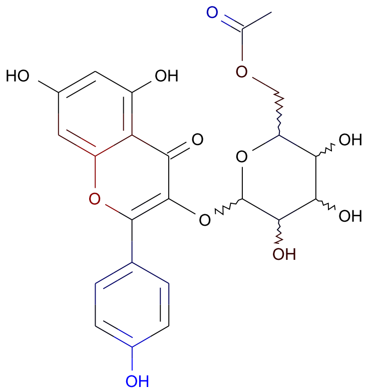


$C_{23}H_{22}O_{12}$
Molecular Weight: 490.41357
ALogP: 0.322
Rotatable Bonds: 6
Acceptors: 12
Donors: 6

## Model Prediction

Prediction: 0.718
Unit: g/kg_body_weight
Mahalanobis Distance: 10
Mahalanobis Distance p-value: 3.81e-005

Mahalanobis Distance: The Mahalanobis distance (MD) is a generalization of the Euclidean distance that accounts for correlations among the X properties. It is calculated as the distance to the center of the training data. The larger the MD, the less trustworthy the prediction.
Mahalanobis Distance p-value: The p-value gives the fraction of training data with an MD greater than or equal to the one for the given sample, assuming normally distributed data. The smaller the p-value, the less trustworthy the prediciton. For highly non-normal X properties (e.g., fingerprints), the MD p-value is wildly inaccurate.

## Structural Similar Compounds

| Name | OXYTETRACYCLINE | 4,4'-DIAMINO-2,2'-STILBENEDISULFONIC ACID.2NaSALT | QUERCETIN |
|---|---|---|---|
| Structure | 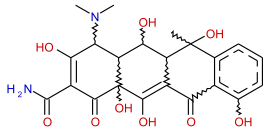 | 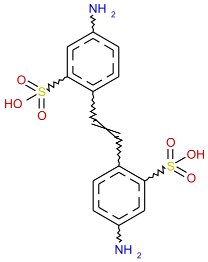 | 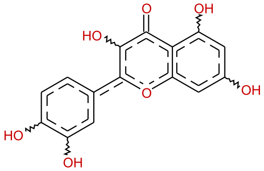 |
| Actual Endpoint (-log C) | 2.36214 | 2.50759 | 2.2016 |
| Predicted Endpoint (-log C) | 2.77834 | 3.26068 | 2.27782 |
| Distance | 0.750 | 0.853 | 0.947 |
| Reference | NCI/NTP TR-315 | NCI/NTP TR-412 | NCI/NTP TR-409 |

## Model Applicability

Unknown features are fingerprint features in the query molecule, but not found or appearing too infrequently in the training set.

1. All properties and OPS components are within expected ranges.

## Feature Contribution

### Top features for positive contribution

| Fingerprint | Bit/Smiles | Feature Structure | Score |
|---|---|---|---|
| FCFP_2 | -1143715940 | 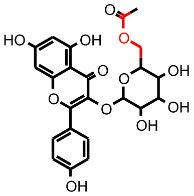 [*]COP(=[*])([*])[*] | 0.095 |

| | | | |
|---|---|---|---|
| FCFP_2 | 1036089772 | 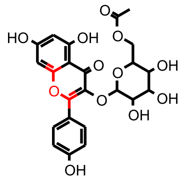<br>[*]C(=[*])O[c](:[*]):<br>[*] | 0.0749 |
| FCFP_2 | 3 | 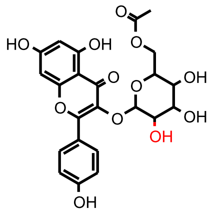<br>[*]O | 0.0737 |

## Top Features for negative contribution

| Fingerprint | Bit/Smiles | Feature Structure | Score |
|---|---|---|---|
| FCFP_2 | 7 | 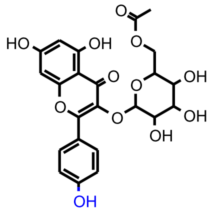<br>[*]O | -0.214 |
| FCFP_2 | -549108873 | 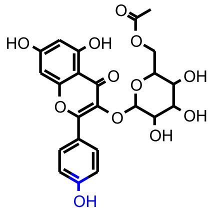<br>[*]:[c](:[*])O | -0.127 |
| FCFP_2 | 1872154524 | 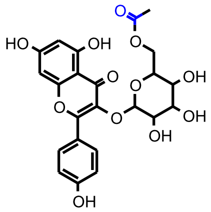<br>[*]P(=O)([*])[*] | -0.105 |

# Remdesivir

# TOPKAT_Rat_Maximum_Tolerated_Dose_Gavage

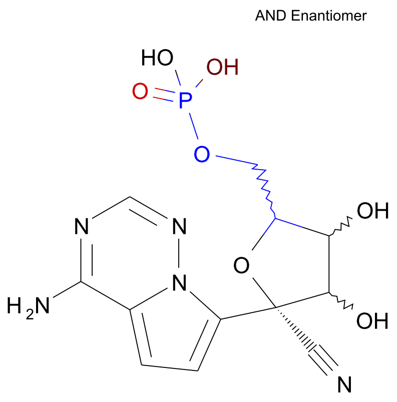


$C_{12}H_{14}N_5O_7P$

Molecular Weight: 371.24262

ALogP: -1.502

Rotatable Bonds: 4

Acceptors: 11

Donors: 5

## Model Prediction

Prediction: 0.000298

Unit: g/kg_body_weight

Mahalanobis Distance: 17.2

Mahalanobis Distance p-value: 5.05e-016

Mahalanobis Distance: The Mahalanobis distance (MD) is a generalization of the Euclidean distance that accounts for correlations among the X properties. It is calculated as the distance to the center of the training data. The larger the MD, the less trustworthy the prediction.
Mahalanobis Distance p-value: The p-value gives the fraction of training data with an MD greater than or equal to the one for the given sample, assuming normally distributed data. The smaller the p-value, the less trustworthy the prediciton. For highly non-normal X properties (e.g., fingerprints), the MD p-value is wildly inaccurate.

## Structural Similar Compounds

| Name | AMPICILLIN TRIHYDRATE | OCHRATOXIN | PENICILLIN VK |
|---|---|---|---|
| Structure | 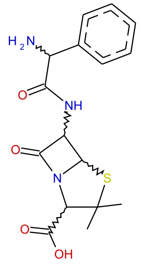 | 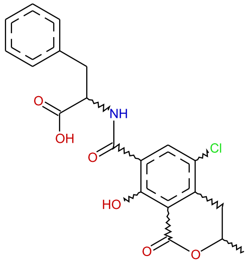 | 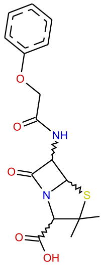 |
| Actual Endpoint (-log C) | 2.36724 | 6.28396 | 2.54455 |
| Predicted Endpoint (-log C) | 2.27651 | 5.12358 | 3.9702 |
| Distance | 1.255 | 1.482 | 1.498 |
| Reference | NCI/NTP TR-318 | NCI/NTP TR-358 | NCI/NTP TR-336 |

## Model Applicability

Unknown features are fingerprint features in the query molecule, but not found or appearing too infrequently in the training set.

1. Num_H_Donors out of range. Value: 5. Training min, max, mean, SD: 0, 3, 0.4375, 0.8311.
2. Num_H_Acceptors out of range. Value: 11. Training min, max, mean, SD: 0, 6, 1.6146, 1.644.
3. Molecular_PolarSASA out of range. Value: 321.97. Training min, max, mean, SD: 0, 223.97, 50.816, 55.15.
4. Molecular_PolarSurfaceArea out of range. Value: 206.26. Training min, max, mean, SD: 0, 138.03, 28.978, 32.1.
5. OPS PC1 out of range. Value: 9.0116. Training min, max, SD, explained variance: -4.0008, 7.9165, 2.861, 0.2531.
6. OPS PC5 out of range. Value: -4.1876. Training min, max, SD, explained variance: -3.4, 4.1587, 1.489, 0.0686.
7. OPS PC9 out of range. Value: -2.7276. Training min, max, SD, explained variance: -2.7086, 2.9267, 1.019, 0.0321.
8. Unknown FCFP_2 feature: 472180098: [*]OP(=O)(O)O
9. Unknown FCFP_2 feature: -836603894: [*]C1[*][*]O[C@]1(C#[*])[c](:[*]):[*]
10. Unknown FCFP_2 feature: -1277879912: [*]C([*])([*])C#N
11. Unknown FCFP_2 feature: -1362791977: [*]C#N
12. Unknown FCFP_2 feature: -332197802: [*][c]1:[*]:[*]:[c](:[*]):n:1:n:[*]
13. Unknown FCFP_2 feature: -124685461: [*]:n:[cH]:n:[*]

## Feature Contribution

## Top features for positive contribution

| Fingerprint | Bit/Smiles | Feature Structure | Score |
|---|---|---|---|
| FCFP_2 | 1 | 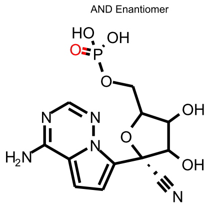  [*]=O | 0.511 |
| FCFP_2 | 3 | 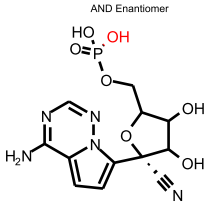  [*]O | 0.104 |

## Top Features for negative contribution

| Fingerprint | Bit/Smiles | Feature Structure | Score |
|---|---|---|---|
| FCFP_2 | 1872154524 | 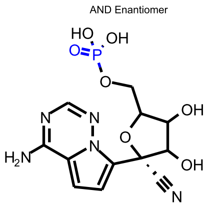  [*]P(=O)([*])[*] | -0.307 |
| FCFP_2 | 0 | 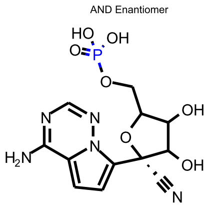  [*]P(=[*])([*])[*] | -0.29 |

| | | | |
|---|---|---|---|
| FCFP_2 | -1272768868 | 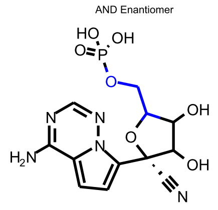 [*]OCC([*])[*] | -0.271 |

# Flavonoid

# TOPKAT_Rat_Maximum_Tolerated_Dose_Gavage

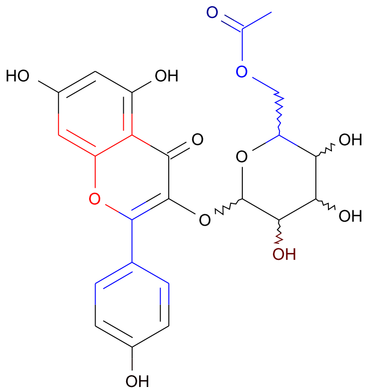

$C_{23}H_{22}O_{12}$

Molecular Weight: 490.41357

ALogP: 0.322

Rotatable Bonds: 6

Acceptors: 12

Donors: 6

## Model Prediction

Prediction: 0.000133

Unit: g/kg_body_weight

Mahalanobis Distance: 15.8

Mahalanobis Distance p-value: 4.48e-014

Mahalanobis Distance: The Mahalanobis distance (MD) is a generalization of the Euclidean distance that accounts for correlations among the X properties. It is calculated as the distance to the center of the training data. The larger the MD, the less trustworthy the prediction.
Mahalanobis Distance p-value: The p-value gives the fraction of training data with an MD greater than or equal to the one for the given sample, assuming normally distributed data. The smaller the p-value, the less trustworthy the prediciton. For highly non-normal X properties (e.g., fingerprints), the MD p-value is wildly inaccurate.

## Structural Similar Compounds

| Name | AMPICILLIN TRIHYDRATE | OCHRATOXIN | PENICILLIN VK |
|---|---|---|---|
| Structure | 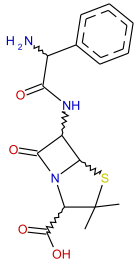 | 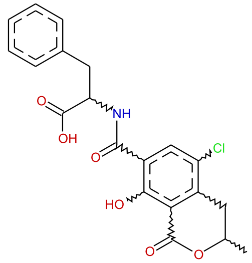 | 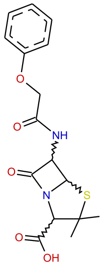 |
| Actual Endpoint (-log C) | 2.36724 | 6.28396 | 2.54455 |
| Predicted Endpoint (-log C) | 2.27651 | 5.12358 | 3.9702 |
| Distance | 1.366 | 1.426 | 1.506 |
| Reference | NCI/NTP TR-318 | NCI/NTP TR-358 | NCI/NTP TR-336 |

## Model Applicability

Unknown features are fingerprint features in the query molecule, but not found or appearing too infrequently in the training set.

1. Molecular_Weight out of range. Value: 490.41. Training min, max, mean, SD: 68.074, 434.63, 171.13, 85.06.
2. Num_H_Donors out of range. Value: 6. Training min, max, mean, SD: 0, 3, 0.4375, 0.8311.
3. Num_H_Acceptors out of range. Value: 12. Training min, max, mean, SD: 0, 6, 1.6146, 1.644.
4. Molecular_PolarSASA out of range. Value: 307.46. Training min, max, mean, SD: 0, 223.97, 50.816, 55.15.
5. Molecular_PolarSurfaceArea out of range. Value: 192.43. Training min, max, mean, SD: 0, 138.03, 28.978, 32.1.
6. OPS PC1 out of range. Value: 12.535. Training min, max, SD, explained variance: -4.0008, 7.9165, 2.861, 0.2531.
7. OPS PC10 out of range. Value: 2.6747. Training min, max, SD, explained variance: -3.9696, 2.3971, 0.982, 0.0298.
8. Unknown FCFP_2 feature: 436915834: [*]OC(=C([*])[*])C(=[*])[*]
9. Unknown FCFP_2 feature: -1549192822: [*]C(=[*])C(=O)[c](:[*]):[*]
10. Unknown FCFP_2 feature: -1678245750: [*]OC(=C([*])[*])[c](:[*]):[*]

## Feature Contribution

| Top features for positive contribution | | | |
|---|---|---|---|
| Fingerprint | Bit/Smiles | Feature Structure | Score |

| | | | |
|---|---|---|---|
| FCFP_2 | 332760439 | 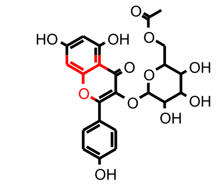 [*]O[c](:[cH]:[*]):[c]([*]):[*] | 0.672 |
| FCFP_2 | 1 | 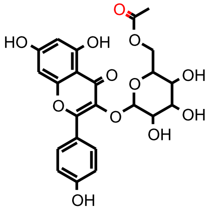 [*]=O | 0.511 |
| FCFP_2 | 3 | 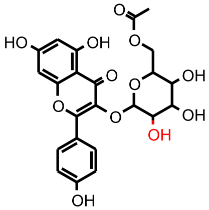 [*]O | 0.104 |

**Top Features for negative contribution**

| Fingerprint | Bit/Smiles | Feature Structure | Score |
|---|---|---|---|
| FCFP_2 | 136597326 | 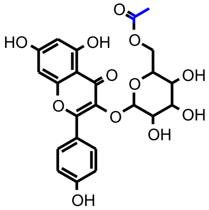 [*]C(=[*])C | -0.489 |
| FCFP_2 | 203677720 | 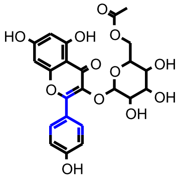 [*]C(=[*])[c](:[cH]:[*]):[cH]:[*] | -0.406 |

| | | | |
|---|---|---|---|
| FCFP_2 | 565998553 | 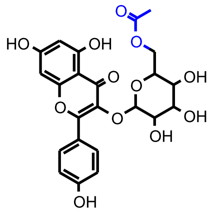 [*]OC(=O)C | -0.348 |

# Remdesivir



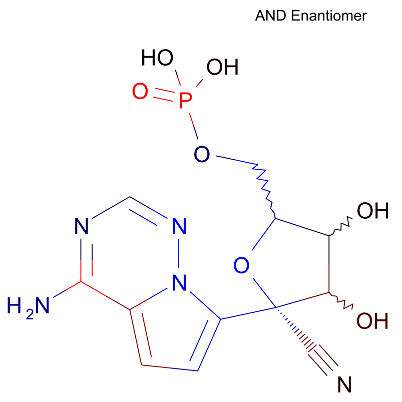


$C_{12}H_{14}N_5O_7P$
Molecular Weight: 371.24262
ALogP: -1.502
Rotatable Bonds: 4
Acceptors: 11
Donors: 5

## Structural Similar Compounds

| Name | 5'-ADENYLIC ACID; POTASSIUM SALT (K STRIPPED) | INOSINATE; DISODIUM SALT (Na STRIPPED) | INOSINE-5'-PHOSPHORIC ACID |
|---|---|---|---|
| Structure | 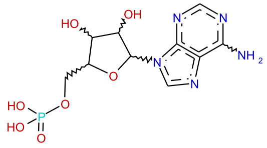 | 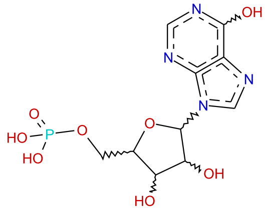 | 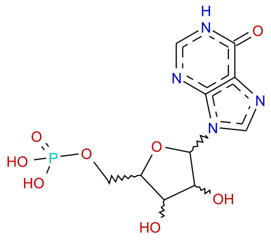 |
| Actual Endpoint (-log C) | 1.49 | 1.34 | 1.338 |
| Predicted Endpoint (-log C) | 2.45569 | 2.92201 | 1.35922 |
| Distance | 0.361 | 0.428 | 0.592 |
| Reference | OYYAA2 4;689;70 | AJINO* -;-;73 | ARTODN 47;77;81 |

## Model Prediction

Prediction: 0.309
Unit: g/kg_body_weight
Mahalanobis Distance: 29.4
Mahalanobis Distance p-value: 1.72e-059

Mahalanobis Distance: The Mahalanobis distance (MD) is a generalization of the Euclidean distance that accounts for correlations among the X properties. It is calculated as the distance to the center of the training data. The larger the MD, the less trustworthy the prediction.
Mahalanobis Distance p-value: The p-value gives the fraction of training data with an MD greater than or equal to the one for the given sample, assuming normally distributed data. The smaller the p-value, the less trustworthy the prediciton. For highly non-normal X properties (e.g., fingerprints), the MD p-value is wildly inaccurate.

## Model Applicability

Unknown features are fingerprint features in the query molecule, but not found or appearing too infrequently in the training set.

1. OPS PC10 out of range. Value: 15.526. Training min, max, SD, explained variance: -6.0395, 14.892, 2.468, 0.0220.
2. Unknown ECFP_2 feature: 1258791451: [*]C1[*][*]O[C@]1(C#[*])[c](:[*]):[*]
3. Unknown ECFP_2 feature: -264833661: [*]C([*])([*])C#N
4. Unknown ECFP_2 feature: -66263742: [*]C([*])([*])[c]1:[cH]:[*]:[*]:n:1:[*]
5. Unknown FCFP_6 feature: 16: [*]:[cH]:[*]
6. Unknown FCFP_6 feature: 472180098: [*]OP(=O)(O)O
7. Unknown FCFP_6 feature: -836603894: [*]C1[*][*]O[C@]1(C#[*])[c](:[*]):[*]
8. Unknown FCFP_6 feature: -332197802: [*][c]1:[*]:[*]:[c](:[*]):n:1:n:[*]
9. Unknown FCFP_6 feature: 4427049: [*]:[cH]:n:n(:[*]):[*]
10. Unknown FCFP_6 feature: -124685461: [*]:n:[cH]:n:[*]
11. Unknown FCFP_6 feature: 1747237384: [*][c](:[*]):n:[cH]:[*]
12. Unknown FCFP_6 feature: -1151884458: [*]:n:[c](N):[c](:[*]):[*]
13. Unknown FCFP_6 feature: 1618154665: [*]:[c]1:[*]:[*]:[cH]:[cH]:1
14. Unknown FCFP_6 feature: 1069584379: [*]:[c](:[*])N

## Feature Contribution

### Top features for positive contribution

| Fingerprint | Bit/Smiles | Feature Structure | Score |
|---|---|---|---|
| ECFP_6 | 642810091 | 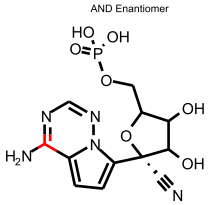 [*][c](:[*]):[*] | 0.281 |
| ECFP_6 | -826638028 | 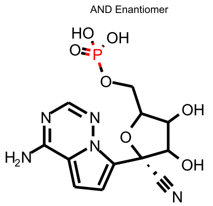 [*]P(=[*])([*])[*] | 0.225 |
| ECFP_6 | 2100964382 | 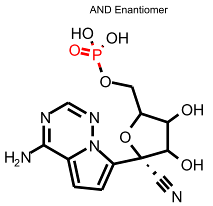 [*]P(=O)([*])[*] | 0.166 |
| **Top Features for negative contribution** | | | |
| **Fingerprint** | **Bit/Smiles** | **Feature Structure** | **Score** |
| ECFP_6 | 683445015 | 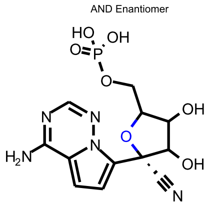 [*]O[*] | -0.266 |

| | | | |
|---|---|---|---|
| ECFP_6 | 655739385 | 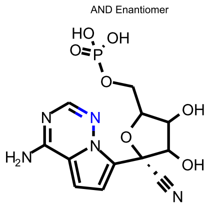<br>[*]:n:[*] | -0.239 |
| FCFP_6 | -1539132615 | 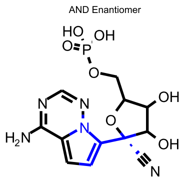<br>[*]C([*])([*])[c]1:[cH]:[*]:[*]:n:1:[*] | -0.2 |

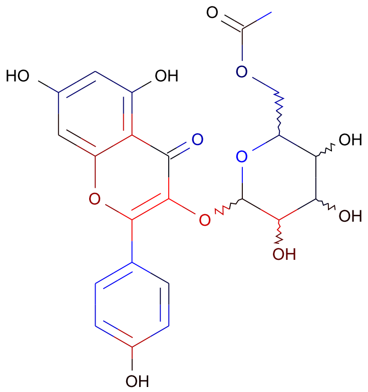

$C_{23}H_{22}O_{12}$
Molecular Weight: 490.41357
ALogP: 0.322
Rotatable Bonds: 6
Acceptors: 12
Donors: 6

## Model Prediction

Prediction: 1.04
Unit: g/kg_body_weight
Mahalanobis Distance: 24.3
Mahalanobis Distance p-value: 3.94e-027

Mahalanobis Distance: The Mahalanobis distance (MD) is a generalization of the Euclidean distance that accounts for correlations among the X properties. It is calculated as the distance to the center of the training data. The larger the MD, the less trustworthy the prediction.
Mahalanobis Distance p-value: The p-value gives the fraction of training data with an MD greater than or equal to the one for the given sample, assuming normally distributed data. The smaller the p-value, the less trustworthy the prediciton. For highly non-normal X properties (e.g., fingerprints), the MD p-value is wildly inaccurate.

## Structural Similar Compounds

| Name | DAUNOMYCIN | AMYGDALIN (D) | METHOTREXATE |
|---|---|---|---|
| Structure | 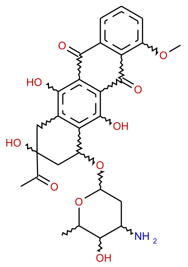 | 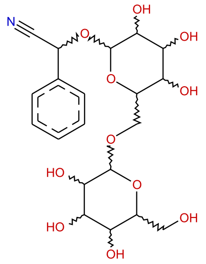 | 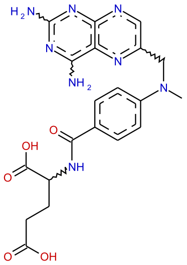 |
| Actual Endpoint (-log C) | 3.196 | 2.943 | 3.527 |
| Predicted Endpoint (-log C) | 3.6117 | 2.51605 | 2.39978 |
| Distance | 0.588 | 0.728 | 0.765 |
| Reference | YKYUA6 25;573;74 | WJMDA2 134;97;81 | NIIRDN 6;841;82 |

## Model Applicability

Unknown features are fingerprint features in the query molecule, but not found or appearing too infrequently in the training set.

1. All properties and OPS components are within expected ranges.
2. Unknown FCFP_6 feature: 16: [*]:[cH]:[*]
3. Unknown FCFP_6 feature: -549108873: [*]:[c](:[*])O
4. Unknown FCFP_6 feature: 74595001: [*]:[cH]:[c](O):[cH]:[*]
5. Unknown FCFP_6 feature: 1618154665: [*]:[c]1:[*]:[*]:[cH]:[cH]:1
6. Unknown FCFP_6 feature: -1678245750: [*]OC(=C([*])[*])[c](:[*]):[*]

## Feature Contribution

| Top features for positive contribution | | | |
|---|---|---|---|
| **Fingerprint** | **Bit/Smiles** | **Feature Structure** | **Score** |
| ECFP_6 | 642810091 | 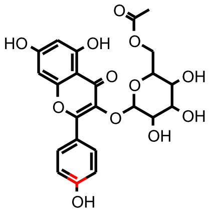 [*][c](:[*]):[*] | 0.281 |

| | | | |
|---|---|---|---|
| ECFP_6 | -560785749 | 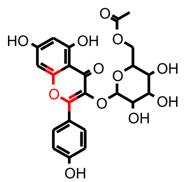 [*]C(=[*])O[c](:[*]):[*] | 0.259 |
| FCFP_6 | 436915834 | 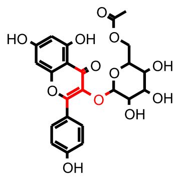 [*]OC(=C([*])[*])C(=[*])[*] | 0.184 |

## Top Features for negative contribution

| Fingerprint | Bit/Smiles | Feature Structure | Score |
|---|---|---|---|
| ECFP_6 | 2106656448 | 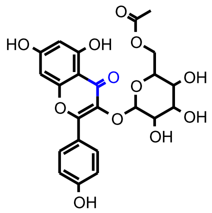 [*]C(=O)[*] | -0.352 |
| ECFP_6 | 683445015 | 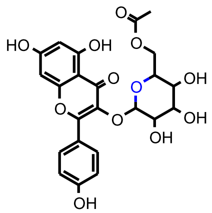 [*]O[*] | -0.266 |
| FCFP_6 | 946589555 | 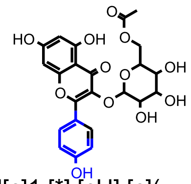 [*][c]1:[*]:[cH]:[c](O):[cH]:[cH]:1 | -0.204 |
